# Synthesis of Tetrahydroazepines through Silyl Aza-Prins Cyclization Mediated by Iron(III) Salts

**DOI:** 10.1021/acs.joc.2c01396

**Published:** 2022-08-17

**Authors:** Victoria Sinka, Israel Fernández, Juan I. Padrón

**Affiliations:** † Instituto de Productos Naturales y Agrobiología (IPNA), CSIC, 38206, Avda. Astrofísico Fco. Sánchez 3, 38206 La Laguna, Tenerife (Spain); ‡ Departamento de Química Orgánica and Centro de Innovación en Química Avanzada (ORFEO-CINQA), Facultad de Ciencias Químicas, Universidad Complutense de Madrid, 28040 Madrid, Spain

## Abstract

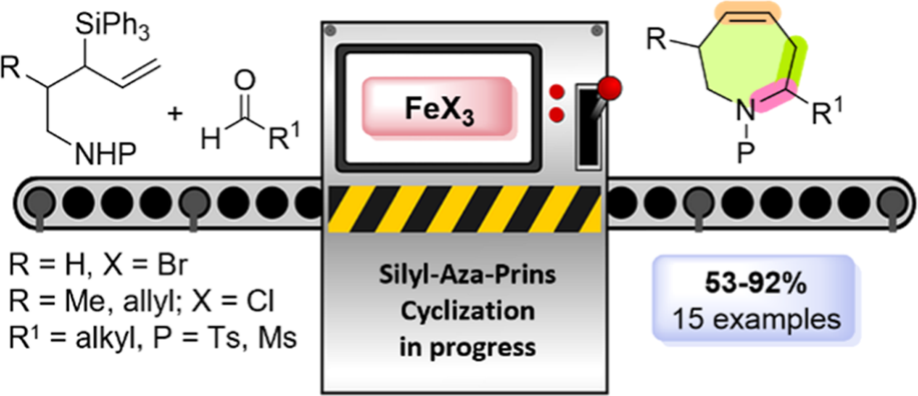

A new methodology for the synthesis of seven-membered unsaturated azacycles (tetrahydroazepines) was developed. It is based on the powerful aza-Prins cyclization in combination with the Peterson-type elimination reaction. In a single reaction step, a C–N, C–C bond and an endocyclic double bond are formed. Under mild reaction conditions and using iron(III) salts as sustainable catalysts, tetrahydroazepines with different degrees of substitution are obtained directly and efficiently. DFT calculations supported the proposed mechanism.

## Introduction

**Figure 1:**
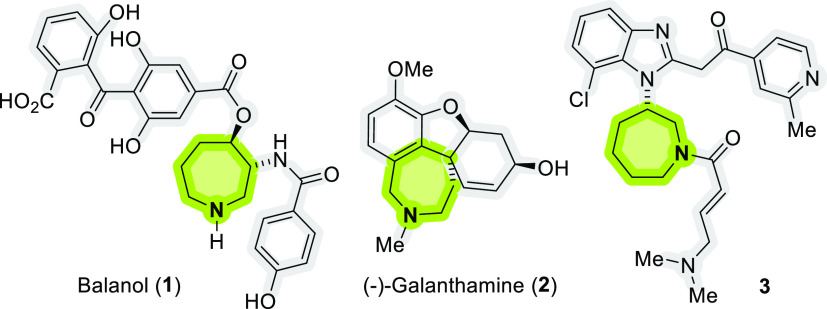
Representative bioactive hydroazepines/tetrahydroazepines.

Unsaturated seven-membered ring azacycles (tetrahydroazepines) are found in numerous natural and non-natural products with remarkable pharmaceutical activity.^1−3^ Also, they serve as substructures of more complex molecules or as precursors of hydroazepines with different biological properties.^4,5^ Most of these natural products come from terrestrial sources such as balanol (**1**), an unusual metabolite isolated from fungus *Verticillium balanoides*, which is a potent inhibitor of PKC^1^ or (−)-galanthamine (**2**), commercially known as Reminyl and used for the symptomatic treatment of Alzheimer’s (Figure 1).^6^ The unnatural azepane **3** proved to be an excellent agent against lung cancer, where the heterocyclic nitrogenous core is essential for its bioactivity (IC_50_ of 4.18 nM) (Figure 1).^2^

**Scheme 1:**
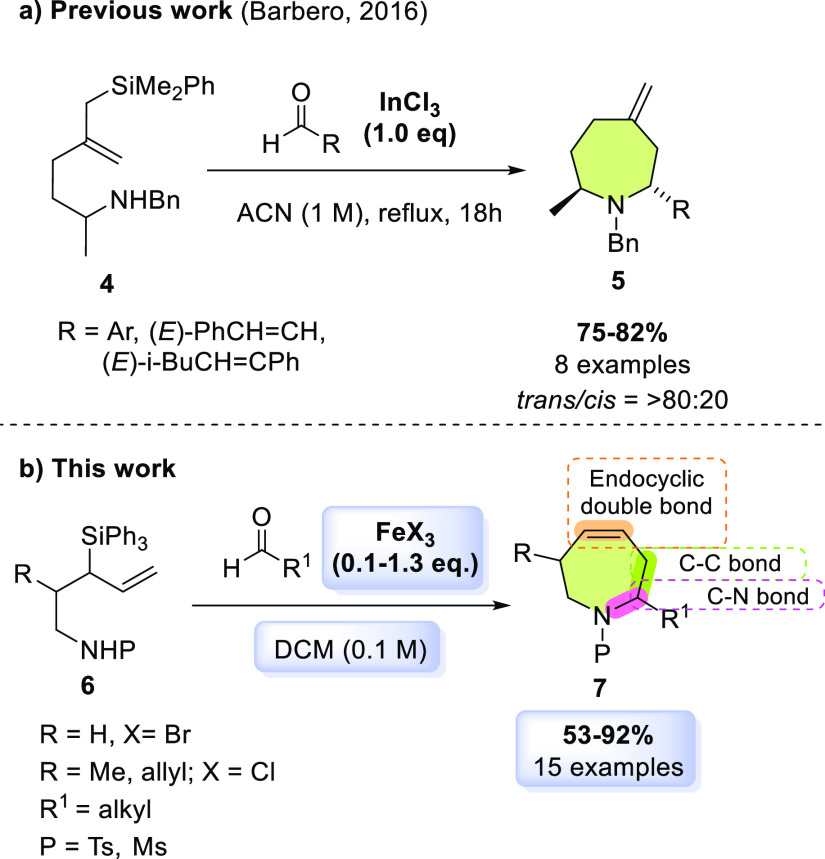
Previous Reports on Silyl Aza-Prins Cyclization and Our Work to Synthesize Unsaturated Seven-Membered Ring Azacycles

Classical methods to synthesize this type of heterocycles include Brønsted or Lewis acid-mediated cyclizations,^7,8^ atom-transfer radical cyclization (ATRC),^9^ cycloadditions,^10,11^ conjugate addition cyclizations,^12,13^ ring expansions (cyclopropanes,^14^ aziridines,^15^ azetidines,^16−18^ 2-cyano-6-oxazolopiperidine^19^), and ring-closing metathesis.^20−25^ Among the different types of acid-mediated cyclizations, the aza-Prins cyclization is a powerful tool for obtaining nitrogenated heterocycles. It has been widely used for the synthesis of piperidines and pyrrolidines.^26−33^ However, there are few examples of synthesis of tetrahydroazepines through this methodology.^7,34^ In 2016, Barbero and co-workers achieved the synthesis of azepane rings, with an exocyclic double bond, through a diastereoselective silyl aza-Prins cyclization mediated by InCl_3_ (Scheme 1).^7^ Nevertheless, this reaction, inspired by the work of the Dobbs’ group, is a relatively high-energy demanding reaction.^30^ Therefore, new methods for the synthesis of seven-membered azacycles via Prins cyclizations remain challenging and highly desirable.

## Results and Discussion

Based on our previous work, which allowed the diastereoselective synthesis of oxepenes through the Prins-Peterson cyclization (PPC) strategy,^35^ we decided to approach the nitrogen version and thus synthesize different tetrahydroazepines. If successful, it would constitute a direct and straightforward method that, using a sustainable iron(III) catalyst, builds a C–N, C–C bond and an endocyclic double bond in a single reaction step (Scheme 1).

We started with the simplest tetrahydroazepines, that is, bearing a single substituent that comes from the corresponding aldehyde. 1-Amino-3-triphenylsilyl-4-pentenes **6a**-**b**, precursors of the silyl aza-Prins cyclization (SAPC), could be accessed in three reaction steps (see Supporting Information). Next, the SAPC assays were performed with different sustainable metal catalysts, based on our previous experience for oxepene synthesis, affording the desired tetrahydroazepine contaminated with variable amounts of the corresponding pyrrolidine. The best results were obtained using substoichiometric amounts (0.1 equiv) of FeBr_3_ and FeCl_3_, the yield and ratio being slightly higher when the former was used (see Supporting Information).

Once FeBr_3_ was set as the best catalyst, we focused on optimizing the reaction conditions to avoid the formation of pyrrolidine **8** resulting from the intramolecular hydroamination side reaction, whose relative stereochemistry was determined by ^1^H-GOESY NMR experiments (Table 1).^36^

**Table 1:**
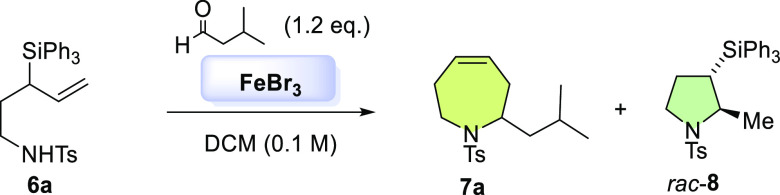
Optimization of Reaction Conditions for SAPC Reaction with FeBr_3_^a^

entry	FeBr_3_ (equiv)	*T* (°C)	time (min)	yield **7a**:**8** (%)
1	0.20	r.t.	15	58:35
2	0.10	r.t.	35	67:10
3	0.05	r.t.	35	82:13
4	0.20	0	30	83:13
**5**	**0.10**	**0**	**35**	**90:0**
6	0.05	0	300	89:7
7	0.20	–20	60	85:0
8	0.10	–20	300	85:0
9	0.05	–20	120	52:35

a Reaction conditions: **6a** (0.20 mmol), isovaleraldehyde (0.24 mmol), FeBr_3_ (0.01–0.04 mmol), dry DCM (0.1 M). Isolated yield. The conversion in all cases was 100%.

**Scheme 2:**
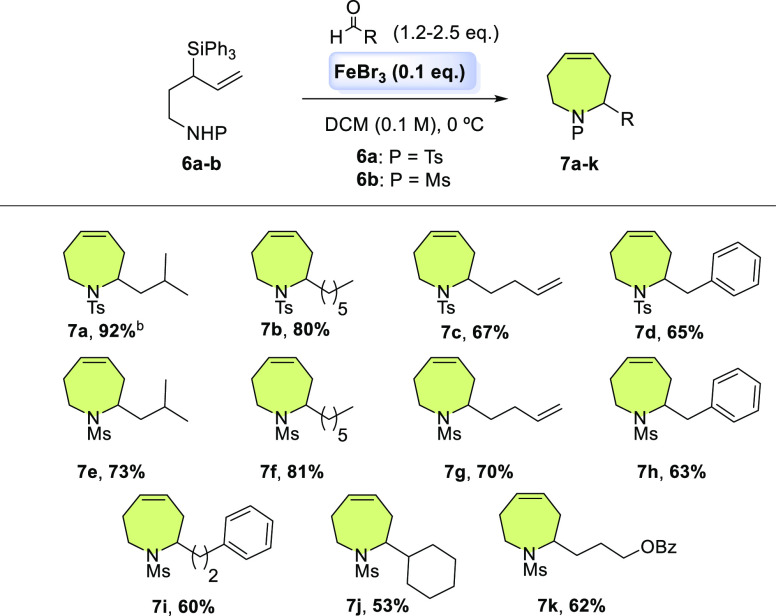
Scope of Monosubstituted Tetrahydroazepines **7** Synthesis From SAPC Reaction conditions: **6a–6b** (0.20 mmol), aldehyde (0.24–0.40 mmol), FeBr_3_ (0.02, mmol), dry DCM (0.1 M). Isolated yield. The conversion in all cases was 100%. The pyrrolidinic byproduct ***rac-8*** was not observed in any example made. ^b^2 g scale of **6a** (4.0 mmol) afforded 60% yield of **7a** (741 mg, 2.41 mmol).

The increase in catalyst loading at room temperature, with respect to that initially tested, favored the undesired azacycle **8** (Table 1, entry 1), whereas the use of 0.05 equiv of FeBr_3_ increased the yield of tetrahydroazepine **7a** (Table 1, entries 2 and 3). We observed that by decreasing the temperature, the formation of the pyrrolidine was reduced (Table 1, entries 4 and 7). However, lower catalyst loading at low temperatures led to long reaction times, which did not avoid the presence of azacycle **8** (Table 1, entries 6 and 9). The best result involved a compromise between 0 °C and 0.10 equiv of FeBr_3_, suppressing the formation of azacycle **8** and increasing the yield of tetrahydroazepine **7a** up to 90% (Table 1, entry 5). With the optimized reaction conditions in hand, we set out to explore the scope of this SAPC. The reaction conditions could be applied to tosylated and mesylated amines **6a-b**, respectively, and to a variety of aldehydes (Scheme 2).

Systems with aliphatic chains such as isovaleraldehyde and heptanal, with both tosylated and mesylated 1-amino-3-triphenylsilyl-4-pentenes **6a–b**, respectively, gave yields of 92, 80, 73, and 81% (tetrahydroazepines **7a**, **7b**, **7e**, and **7f**) (Scheme 2). Likewise, good results were observed with 4-pentenal, which led to tetrahydroazepines **7c** and **7g** with an endocyclic and an exocyclic olefin. Reaction with phenylacetaldehyde and hydrocinnamaldehyde allowed a phenyl substituent to be incorporated into the heterocycles **7d**, **7h**, and **7i** in 65, 63, and 60% yield, respectively. Our SAPC protocol also worked when using an aldehyde with a benzoate carrier chain, so future derivatizations of tetrahydroazepine **7k** are feasible. However, there was no reactivity with aromatic aldehydes.

Subsequent efforts were devoted to extend this methodology to the synthesis of disubstituted tetrahydroazepine **10**. Once we synthetized the precursor amines **9** substituted at the β position with methyl and allyl groups (see Supporting Information), we made the preliminary SAPC assays. On this occasion, FeCl_3_, which also gave good yields in the synthesis of monosubstituted tetrahydroazepines, was the catalyst that provided the best yields for the corresponding disubstituted tetrahydroazepines (see Supporting Information). The next optimization step consisted of adjusting the amount of FeCl_3_, the concentration of the solvent, and the temperature (Table 2).

**Table 2:**
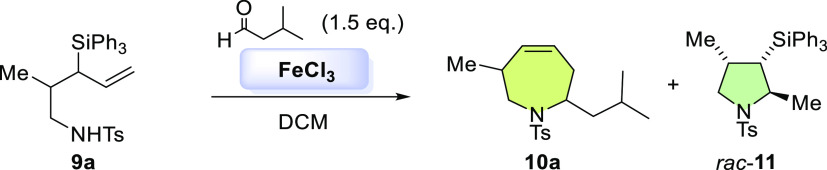
Optimization of Reaction Conditions for SAPC Reaction with FeCl_3_^a^

entry	FeCl_3_ (equiv)	DCM (M)	*T* (°C)	time (min)	yield^b^^,^^g^**10a**:**11** (%)
1^c^	0.1	0.1	10	360	13:8
2	0.3	0.1	10	140	45:18
3	0.5	0.1	10	120	42:15
4^d^	0.3	0.1	0	360	28:18
5^e^	0.3	0.1	0 → r.t.	300	40:24
6	0.3	0.1	r.t.	240	40:22
7	0.6	0.2	r.t.	100	53:18
8	0.3	0.3	r.t.	120	47:22
9^f^	1.0 + 0.3	0.1	–20 → r.t.	220	72:4

a Reaction conditions: **9a** (0.098 mmol), isovaleraldehyde (0.15 mmol), FeCl_3_ (0.098–0.13 mmol), dry DCM.

b Isolated yield.

c Conversion of 63%.

d Conversion of 54%.

e The order of addition was changed: DCM, aldehyde, FeCl_3_, and amine **9a** in portions.

f Initial FeCl_3_ load was 1.0 equiv at −20 °C. After 3 h of reaction, another 0.3 equiv of catalyst was added at −20 °C, and after 10 min the bath was removed, leaving the reaction at room temperature.

g **10a** is obtained as an inseparable mixture of *cis*/*trans* diastereomers by flash chromatography. The *trans* isomer was identified as the major one through NMR studies see Supporting Information.

Because the 1-amino-3-triphenylsilyl-4-pentene **9a** showed a different reactivity than **6a–b** (see above), we decided to adjust the SAPC conditions again (Table 2). The amount of FeCl_3_ was varied between 0.1 and 0.5 equiv, maintaining the temperature at 10 °C, the best result being 30 mol % of the catalyst (Table 2, entries 1–3). Dropping to 0 °C prevented complete consumption of starting material, and lower yield of tetrahydroazepine **10a** was observed (Table 2, entries 4 and 5). In addition, at room temperature, the concentration was increased to favor the intermolecular reaction to generate the desired disubstituted tetrahydroazepine **10a**. However, seven-membered azacycle **10a** and pyrrolidine byproduct **11** were obtained in 50 and 20% yield on average, respectively (Table 2, entries 6–8). Therefore, we decided to increase the catalyst loading up to 1.0 equiv at low temperatures, to avoid the formation of pyrrolidine **11**. The starting material was completely consumed, adding an extra 0.3 equiv of FeCl_3_ at −20 °C and allowing the reaction to reach the room temperature. Thus, a conversion of 100% and the predominant formation (95%) of tetrahydroazepine **10a** was observed with 72% yield.

**Scheme 3:**
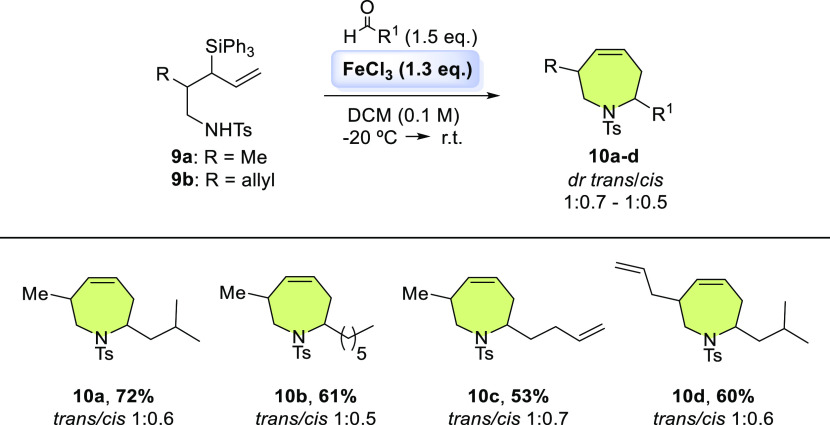
Scope of Disubstituted Tetrahydroazepines Synthesis from Silyl Aza-Prins Cyclization Reaction conditions: **9a–b** (0.098 mmol), aldehyde (0.15 mmol), FeCl_3_ (0.13, mmol), dry DCM (0.1 M). Isolated yield. The conversion in all cases was 100%. The pyrrolidinic byproduct **rac-11** was observed in small amounts (4% yield) in each example.

The scope of the SAPC reaction with amines **9a–b** was then tested (Scheme 3). On the one hand, the 1-amino-3-triphenylsilyl-4-pentene **9a** bearing a methyl unit was reactive with aliphatic aldehydes such as isovaleraldehyde and heptanal, giving tetrahydroazepines **10a** and **10b** in 72 and 61% yield, respectively. Likewise, it was possible to obtain tetrahydroazepine **10c** through SAPC with 4-pentenal (Scheme 3). On the other hand, the precursor amine **9b** bearing an allyl moiety at β position showed lower reactivity than the amine **9a**. Only SAPC with isovaleraldehyde was possible generating tetrahydroazepine **10d** in 60% yield (Scheme 3).

**Scheme 4:**
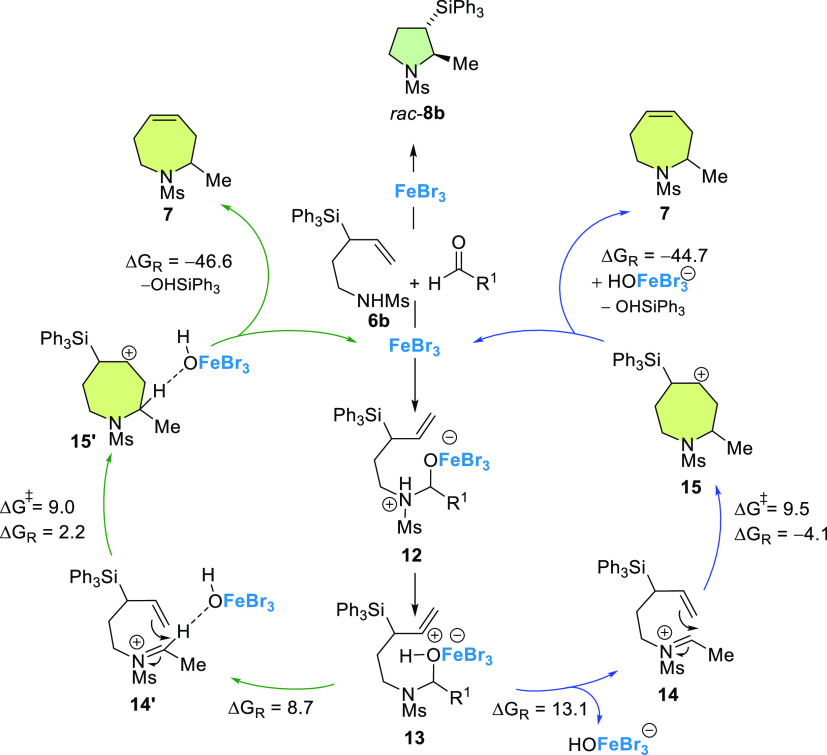
Mechanistic Proposal for SAPC Supported by DFT Calculations

The mechanistic proposal for this transformation is based on that reported for the analogous process forming oxepenes^33^ (Scheme 4). First, a condensation occurs between the 1-amino-3-triphenylsilyl-4-pentene **6a** and the Lewis acid–activated aldehyde. The zwitterionic species **12** leads to the amino-alcohol **13** and evolves to the iminium ion **14**. This last species is intramolecularly trapped by the double bond, generating the carbocation **15**, stabilized by the presence of the silyl group (β effect). Intermediate **15** undergoes a Peterson-type elimination, leading to tetrahydroazepines **7** and triphenylsilanol as a byproduct (Scheme 4).

Density functional theory (DFT) calculations were carried out [PCM(CH2Cl2)–B3LYP-D3/def2-TZVPP//PCM (CH2Cl2)–B3LYP-D3/def2-SVP level] to gain more insights into the proposed reaction mechanism. In particular, we focused on the key intramolecular cyclization step involving **13**, where the tosyl group was replaced by a mesyl group and R^1^ = Me. Our calculations indicate that the release of the anion FeBr_3_OH^–^, thus forming **14**, which is endergonic (Δ*G*_R_ = 13.1 kcal/mol), and the subsequent nucleophilic cyclization possesses a barrier of only 9.5 kcal/mol. Therefore, from **13**, this S_N_1-type reaction is compatible with the reaction conditions used in the experiments. Alternatively, **13** can evolve into an intimate ion pair **14′**^**=,**^ where the FeBr_3_OH^–^ fragment is tightly bonded to the cation **14** in a less endergonic reaction (Δ*G*_R_ = 8.7 kcal/mol). Then, a similar nucleophilic cyclization occurs with a rather similar, low activation barrier of 9.0 kcal/mol. The initial slightly endergonic C–O(H)FeBr_3_ bond rupture in **15** is efficiently compensated by the final Peterson elimination, which is strongly exergonic (Δ*G*_R_ > −45 kcal/mol) therefore driving the complete catalytic cycle forward (see computed profiles in the Supporting Information).

## Conclusions

In conclusion, we have developed an efficient and straightforward approach for the formation of mono- and disubstituted seven-membered Δ^4^-unsaturated azacycles. A variety of mono- and disubstituted tetrahydroazepines can be accessed through SAPC, and in a single reaction step, C–C, C–N, and endocyclic C=C bonds are formed. In addition, it has been shown that the functionalization of the precursor amines determines their reactivity. Increasing the substitution of tetrahydroazepine up to three substituents is currently ongoing in our laboratory.

## Experimental Section

### General Information

Reagents were obtained from commercial sources (Sigma-Aldrich, Merck, Alfa Aesar), without further purification. Solvents (DCM, Et_2_O, THF, and DMF) were used from the PureSolv system. The dispensing system allows easy access to the anhydrous solvents. EtOH was purified by distillation and dried following the procedure in the literature.^35^ Chemical reactions and the separation of the crudes were monitored by thin-layer chromatography (TLC). TLC was performed on aluminum foil sheets 60 F254 manufactured by MERCK. The solvent or solvent mixture was *n*-hexane/ethyl acetate (EtOAc) in different ratios. Flash column chromatography was performed using silica gel (0.015–0.04 mm) and *n*-hexane/EtOAc solvent systems. Automated flash column chromatography was performed using the Biotage Isolera System (Isolera Prime). It includes simultaneous UV detection on all wavelengths and baseline correction, which enable detection of poor UV absorbing compounds. NMR spectra were recorded on Bruker Avance instruments. ^1^H NMR spectra were recorded at 400, 500, and 600 MHz, and ^13^C NMR spectra were recorded at 100, 125, and 150 MHz, VTU 298.0 K. The residual solvent peak was used as an internal reference (CDCl_3_: δ_H_ 7.26, δ_C_ 77.0).^36^ High-resolution mass spectra were recorded on an LCT Premier XE mass spectrometer. It is provided with different ionization sources: electrospray (ESI), an atmospheric pressure chemical ionization (APCI) source, and an orthogonal acceleration time of flight (oa-TOF) analyzer that provides high sensitivity, resolution, and accurate mass measurement.

#### General Procedure (1) for Silyl Aza-Prins Cyclization of 1-Amino-3-triphenylsilyl-4-pentenes **6a–b**

To a solution of amines **6a–b** (0.30–0.12 mmol, 1.0 equiv) in dry DCM (3.0–1.2 mL, 0.1 M) at 0 °C were added the aldehyde (0.45–0.18 mmol, 1.5 equiv) and the catalyst (0.030–0.012 mmol, 0.1 equiv). Once the reaction was complete, checked by TLC, it was quenched with water. The layers were separated, and the aqueous phase was extracted three times with DCM. The combined organic layers were dried over anhydrous MgSO_4_, filtrated, and concentrated under reduced pressure. The residue was purified by flash silica gel column chromatography (*n*-hexane/EtOAc solvent system).

#### General Procedure (2) for Silyl Aza-Prins Cyclization of 1-Amino-3-triphenylsilyl-4-pentene **9a–b**

To a solution of amines **9a–b** (0.098–0.074 mmol, 1.0 equiv) in DCM (1.0–0.7 mL, 0.1 M) at −20 °C under inert atmosphere were added the aldehyde (0.15–0.11 mmol, 1.5 equiv) and FeCl_3_ (0.098–0.074 mmol, 1.0 equiv). After stirring the mixture for 2 h at −20 °C, an extra amount of FeCl_3_ (0.029–0.022 mmol, 0.3 equiv) was added, and then, the bath was removed. The reaction mixture was stirred at room temperature for 30 min. Once the reaction was completed, it was quenched with water. The phases were separated, and the aqueous layer was extracted with 3× DCM. The combined organic phases were dried over anhydrous MgSO_4_, filtered, and concentrated under reduced pressure. The residue was purified by flash silica gel column chromatography (*n*-hexane/DCM/MeOH 69:29:2 solvent system).

#### 2-Isobutyl-1-tosyl-2,3,6,7-tetrahydro-1*H*-azepine (**7a**)

Following the general procedure (1), to a solution of amine **6a** (80 mg, 0.16 mmol, 1.0 equiv) in 1.6 mL of dry DCM (0.1 M) at 0 °C were added isovaleraldehyde (21 μL, 0.19 mmol, 1.2 equiv) and FeBr_3_ (4.8 mg, 0.019 mmol, 0.1 equiv) to obtain 45 mg of tetrahydroazepine **7a** as a pale yellow oil (0.147 mmol, 92%). *R*_f_ = 0.63 (*n*-hexane/EtOAc 80:20), ^1^H NMR (CDCl_3_, 400 MHz): δ = 7.69 (d, *J* = 8.1 Hz, 2H), 7.26 (d, *J* = 7.3 Hz, 2H), 5.70 (m, 1H), 5.55 (m, 1H), 4.13 (m, 1H), 3.66 (brddd, *J* = 14.5, 4.8 & 3.4 Hz, 1H), 3.14 (brddd, *J* = 14.0, 11.0 & 2.2 Hz, 1H), 2.40 (s, 3H), 2.38–2.27 (m, 2H), 2.15 (m, 2H), 1.37 (m, 3H), 0.82 (d, *J* = 6.4 Hz, 3H), 0.80 (d, *J* = 6.3 Hz, 3H); ^13^C{^1^H}-NMR (CDCl_3_, 125 MHz): δ = 142.8 (C), 138.6 (C), 130.7 (CH), 129.5 (2× CH), 127.2 (CH), 127.1 (2× CH), 53.4 (CH), 41.2 (CH_2_), 40.2 (CH_2_), 32.5 (CH_2_), 30.3 (CH_2_), 24.6 (CH), 22.9 (CH_3_), 22.3 (CH_3_), 21.5 (CH_3_); HRMS (ESI^+^): *m*/*z* [M + Na]^+^ calcd for C_17_H_25_NO_2_NaS, 330.1505; found, 330.1504.

#### 2,3-Trans-2-methyl-1-tosyl-3-(triphenylsilyl) pyrrolidine (**rac-8**)

A solution of 60 mg of amine **6a** (0.12 mmol, 1.0 equiv) in 1.2 mL of dry DCM (0.1 M) at room temperature in the presence of 59 mg of FeBr_3_ (0.024 mmol, 0.20 equiv) gave 21 mg of pyrrolidine ***rac-8*** (0.042 mmol, 35% yield) as a white amorphous solid. *R*_f_ = 0.52 (*n*-hexane/EtOAc 80:20); ^1^H NMR (CDCl_3_, 400 MHz): δ 7.50 (d, *J* = 8.1 Hz, 2H), 7.47–7.39 (m, 9H), 7.37–7.30 (m, 6H), 7.14 (d, *J* = 8.1 Hz, 2H), 3.77 (m, 1H), 3.40 (m, 1H), 3.29 (m, 1H), 2.42 (s, 3H), 1.95 (m, 2H), 1.29 (m, 4H); ^13^C{^1^H}-NMR (CDCl_3_, 100 MHz): δ 142.7 (C), 135.9 (6× CH), 135.7 (C), 135.4 (C), 133.0 (2× C), 129.7 (3× CH), 129.5 (2× CH), 128.0 (6× CH), 127.3 (2× CH), 59.4 (CH), 49.3 (CH_2_), 33.5 (CH), 28.8 (CH_2_), 24.0 (CH_3_), 21.5 (CH_3_); HRMS (ESI^+^): *m*/*z* [M + Na]^+^ calcd for C_30_H_31_NO_2_NaSSi, 520.1742; found, 520.1745.

#### 2-Hexyl-1-tosyl-2,3,6,7-tetrahydro-1*H*-azepine (**7b**)

Following the general procedure (1), to a solution of amine **6a** (0.150 mg, 0.30 mmol, 1.0 equiv) in 3.0 mL of dry DCM (0.1 M) at 0 °C were added heptanal (51 μL, 0.36 mmol, 1.2 equiv) and FeBr_3_ (9.0 mg, 0.030 mmol, 0.1 equiv) to obtain 81 mg of tetrahydroazepine **7b** as a pale yellow oil (0.24 mmol, 80%). *R*_f_ = 0.57 (*n*-hexane/EtOAc 80:20); ^1^H NMR (CDCl_3_, 400 MHz): δ = 7.70 (d, *J* = 8.2 Hz, 2H), 7.26 (d, *J* = 8.3 Hz, 2H), 5.69 (m, 1H), 5.56 (m, 1H), 4.03 (m, 1H), 3.68 (brddd, *J* = 14.5, 5.1 & 3.1 Hz, 1H), 3.14 (brddd, *J* = 13.3, 10.9 & 2.1 Hz, 1H), 2.41 (s, 3H), 2.38–2.28 (m, 2H), 2.19 (m, 2H), 1.46 (m, 2H), 1.29–1.11 (m, 6H), 1.10–0.98 (m, 2H), 0.85 (t, *J* = 7.0 Hz, 3H); ^13^C{^1^H}-NMR (CDCl_3_, 100 MHz): δ = 142.7 (C), 138.5 (C), 130.6 (CH), 129.4 (2× CH), 127.0 (CH), 126.9 (2× CH), 55.4 (CH), 41.3 (CH_2_), 32.3 (CH_2_), 31.6 (CH_2_), 31.2 (CH_2_), 30.5 (CH_2_), 29.0 (CH_2_), 26.1 (CH_2_), 22.5 (CH_2_), 21.4 (CH_3_), 14.0 (CH_3_); HRMS (ESI^+^): *m*/*z* [M + Na]^+^ calcd for C_19_H_29_NO_2_NaS, 358.1817; found, 358.1821.

#### 2-(But-3-en-1-yl)-1-tosyl-2,3,6,7-tetrahydro-1*H*-azepine (**7c**)

Following the general procedure (1), to a solution of amine **6a** (0.150 mg, 0.30 mmol, 1.0 equiv) in 3.0 mL of dry DCM (0.1 M) at 0 °C were added 4-pentenal (61 μL, 0.60 mmol, 2.0 equiv) and FeBr_3_ (9.0 mg, 0.030 mmol, 0.1 equiv) to obtain 61 mg of tetrahydroazepine **7c** as a pale yellow oil (0.20 mmol, 67%). *R*_f_ = 0.54 (*n*-hexane/EtOAc 80:20); ^1^H NMR (CDCl_3_, 400 MHz): δ = 7.68 (d, *J* = 8.4 Hz, 2H), 7.26 (d, *J* = 7.8 Hz, 2H), 5.71 (m, 2H), 5.54 (m, 1H), 4.94 (m, 2H), 4.06 (m, 1H), 3.65 (ddd, *J* = 14.5, 5.4 & 3.3 Hz, 1H), 3.18 (ddd, *J* = 14.6, 10.6 & 2.4 Hz, 1H), 2.40 (s, 3H), 2.37–2.26 (m, 2H), 2.18 (m, 2H), 1.90 (m, 2H), 1.59 (m, 2H); ^13^C{^1^H}-NMR (CDCl_3_, 100 MHz): δ = 142.8 (C), 138.3 (C), 137.8 (CH), 130.8 (CH), 129.5 (2× CH), 127.0 (2× CH), 126.7 (CH), 114.8 (CH_2_), 55.2 (CH), 41.5 (CH_2_), 31.8 (CH_2_), 30.7 (CH_2_), 30.3 (CH_2_), 30.2 (CH_2_), 21.4 (CH_3_); HRMS (ESI^+^): *m*/*z* [*M* + Na]^+^ calcd for C_17_H_23_NO_2_NaS, 328.1347; found, 328.1349.

#### 2-Benzyl-1-tosyl-2,3,6,7-tetrahydro-1*H*-azepine (**7d**)

Following the general procedure (1), to a solution of amine **6a** (0.150 g, 0.30 mmol, 1.0 equiv) in 3.0 mL of dry DCM (0.1 M) at 0 °C were added phenylacetaldehyde (43 μL, 0.36 mmol, 1.2 equiv) and FeBr_3_ (9.0 mg, 0.030 mmol, 0.1 equiv) to obtain 67 mg of tetrahydroazepine **7d** as a pale yellow oil (0.195 mmol, 65%). *R*_f_ = 0.46 (*n*-hexane/EtOAc 80:20); ^1^H NMR (CDCl_3_, 400 MHz): δ = 7.62 (m, 2H), 7.23 (m, 5H), 7.12 (m, 2H), 5.73 (m, 1H), 5.55 (m, 1H), 4.30 (m, 1H), 3.64 (ddd, *J* = 14.4, 5.7 & 3.4 Hz, 1H), 3.33 (ddd, *J* = 14.4, 10.4 & 2.6 Hz, 1H), 2.84 (m, 2H), 2.47–2.34 (m, 4H), 2.26 (m, 2H), 2.09 (m, 1H); ^13^C{^1^H}-NMR (CDCl_3_, 100 MHz): δ = 142.8 (C), 138.5 (C), 137.9 (C), 130.8 (CH), 129.5 (2× CH), 129.2 (2× CH), 128.4 (2× CH), 127.0 (2× CH), 126.5 (CH), 126.4 (CH), 58.1 (CH), 41.9 (CH_2_), 38.8 (CH_2_), 30.6 (CH_2_), 30.5 (CH_2_), 21.4 (CH_3_); HRMS (ESI^+^): *m*/*z* [M + Na]^+^ calcd for C_20_H_23_NO_2_NaS, 364.1347; found, 364.1349.

#### 2-Isobutyl-1-(methylsulfonyl)-2,3,6,7-tetrahydro-1*H*-azepine (**7e**)

Following the general procedure (1), to a solution of amine **6b** (0.500 g, 1.19 mmol, 1.0 equiv) in 12 mL of dry DCM (0.1 M) at 0 °C were added isovaleraldehyde (0.19 mL, 1.79 mmol, 1.5 equiv) and FeBr_3_ (35 mg, 0.12 mmol, 0.1 equiv) to obtain 0.201 g of tetrahydroazepine **7e** as a pale yellow oil (0.87 mmol, 73%). *R*_f_ = 0.49 (*n*-hexane/EtOAc 70:30); ^1^H NMR (CDCl_3_, 400 MHz): δ = 5.78 (m, 1H), 5.68 (m, 1H), 4.07 (dq, *J* = 6.9 & 4.0 Hz, 1H), 3.66 (dt, *J* = 14.9 & 4.0 Hz, 1H), 3.21 (ddd, *J* = 14.6, 11.5 & 2.9 Hz, 1H), 2.87 (s, 3H), 2.48 (m, 2H), 2.28 (m, 2H), 1.53 (m, 2H), 1.38 (m, 1H), 0.93 (brd, *J* = 1.6 Hz, 3H), 0.91 (brd, *J* = 1.6 Hz, 3H); ^13^C{^1^H}-NMR (CDCl_3_, 100 MHz): δ = 130.5 (CH), 127.1 (CH), 54.1 (CH), 41.3 (CH_2_), 40.6 (CH_3_), 40.5 (CH_2_), 33.1 (CH_2_), 30.7 (CH_2_), 24.7 (CH), 22.9 (CH_3_), 22.3 (CH_3_); HRMS (ESI^+^): *m*/*z* [*M* + Na]^+^ calcd for C_11_H_21_NO_2_NaS, 254.1191; found, 254.1191.

#### 2-Hexyl-1-(methylsulfonyl)-2,3,6,7-tetrahydro-1*H*-azepine (**7f**)

Following the general procedure (1), to a solution of amine **6b** (50 mg, 0.12 mmol, 1.0 equiv) in 1.2 mL of dry DCM (0.1 M) at 0 °C were added heptanal (25 μL, 0.18 mmol, 1.5 equiv) and FeBr_3_ (3.6 mg, 0.012 mmol, 0.1 equiv) to obtain 25 mg of tetrahydroazepine **7f** as a pale yellow oil (0.097 mmol, 81%). *R*_f_ = 0.62 (*n*-hexane/EtOAc 70:30); ^1^H NMR (CDCl_3_, 400 MHz): δ = 5.76 (m, 1H), 5.68 (m, 1H), 3.95 (m, 1H), 3.68 (dt, *J* = 14.9 & 4.1 Hz, 1H), 3.22 (ddd, *J* = 14.7, 11.5 & 3.0 Hz, 1H), 2.86 (s, 3H), 2.47 (m, 2H), 2.31 (m, 2H), 1.66 (m, 1H), 1.52 (m, 1H), 1.27 (m, 8H), 0.89 (t, *J* = 6.8 Hz, 3H); ^13^C{^1^H}-NMR (CDCl_3_, 125 MHz): δ = 130.5 (CH), 126.9 (CH), 56.3 (CH), 40.7 (CH_2_), 40.4 (CH_3_), 32.7 (CH_2_), 32.5 (CH_2_), 31.8 (CH_2_), 30.8 (CH_2_), 29.2 (CH_2_), 26.2 (CH_2_), 22.6 (CH_2_), 14.1 (CH_3_); HRMS (ESI^+^): *m*/*z* [*M* + Na]^+^ calcd for C_13_H_25_NO_2_NaS: 282.1504; found, 282.1506.

#### 2-(But-3-en-1-yl)-1-(methylsulfonyl)-2,3,6,7-tetrahydro-1*H*-azepine (**7g**)

Following the general procedure (1), to a solution of amine **6b** (50 mg, 0.12 mmol, 1.0 equiv) in 1.2 mL of dry DCM (0.1 M) at 0 °C were added 4-pentenal (18 μL, 0.18 mmol, 1.5 equiv) and FeBr_3_ (3.6 mg, 0.012 mmol, 0.1 equiv) to obtain 19 mg of tetrahydroazepine **7g** as a pale yellow oil (0.084 mmol, 70%). *R*_f_ = 0.51 (*n*-hexane/EtOAc 70:30); ^1^H NMR (CDCl_3_, 400 MHz): δ = 5.79 (m, 2H), 5.68 (m, 1H), 5.04 (dq, *J* = 17.1 & 1.6 Hz, 1H), 4.98 (dd, *J* = 10.3 & 1.6 Hz, 1H), 3.98 (dq, *J* = 7.0 & 4.2 Hz, 1H), 3.70 (dt, *J* = 15.0 & 4.3 Hz, 1H), 3.25 (ddd, *J* = 14.6, 11.4 & 3.0 Hz, 1H), 2.87 (s, 3H), 2.48 (m, 2H), 2.33 (m, 2H), 2.04 (m, 2H), 1.79 (m, 1H), 1.63 (m, 1H); ^13^C{^1^H}-NMR (CDCl_3_, 125 MHz): δ = 137.7 (CH), 130.6 (CH), 126.7 (CH), 115.1 (CH_2_), 55.9 (CH), 40.8 (CH_2_), 40.4 (CH_3_), 32.4 (CH_2_), 31.8 (CH_2_), 30.7 (CH_2_), 30.3 (CH_2_); HRMS (ESI^+^): *m*/*z* [*M* + Na]^+^ calcd for C_11_H_19_NO_2_NaS, 252.1034; found, 252.1035.

#### 2-Benzyl-1-(methylsulfonyl)-2,3,6,7-tetrahydro-1*H*-azepine (**7h**)

Following the general procedure (1), to a solution of amine **6b** (50 mg, 0.12 mmol, 1.0 equiv) in 1.2 mL of dry DCM (0.1 M) at 0 °C were added phenylacetaldehyde (20 μL, 0.18 mmol, 1.5 equiv) and FeBr_3_ (3.6 mg, 0.012 mmol, 0.1 equiv) to obtain 20 mg of tetrahydroazepine **7h** as a pale yellow oil (0.076 mmol, 63%). *R*_f_ = 0.44 (*n*-hexane/EtOAc 70:30); ^1^H NMR (CDCl_3_, 400 MHz): δ = 7.33–7.27 (m, 2H), 7.25–7.19 (m, 3H), 5.81 (m, 1H), 5.69 (m, 1H), 4.25 (dq, *J* = 7.0 & 4.2 Hz, 1H), 3.64 (dt, *J* = 15.0 & 4.1 Hz, 1H), 3.28 (ddd, *J* = 14.5, 11.4 & 2.9 Hz, 1H), 3.03 (dd, *J* = 13.4 & 7.0 Hz, 1H), 2.82 (dd, *J* = 13.5 & 7.5 Hz, 1H), 2.56–2.44 (m, 4H), 2.43–2.26 (m, 3H); ^13^C{^1^H}-NMR (CDCl_3_, 125 MHz): δ = 138.4 (C), 130.7 (CH), 129.3 (2× CH), 128.5 (2× CH), 126.7 (CH), 126.6 (CH), 58.5 (CH), 41.0 (CH_2_), 39.3 (CH_3_), 39.1 (CH_2_), 32.4 (CH_2_), 30.9 (CH_2_); HRMS (ESI^+^): *m*/*z* [M + Na]^+^ calcd for C_14_H_19_NO_2_NaS, 288.1034; found, 288.1036.

#### 1-(Methylsulfonyl)-2-phenethyl-2,3,6,7-tetrahydro-1*H*-azepine (**7i**)

Following the general procedure (1), to a solution of amine **6b** (50 mg, 0.12 mmol, 1.0 equiv) in 1.2 mL of dry DCM (0.1 M) at 0 °C were added hydrocinnamaldehyde (26 μL, 0.18 mmol, 1.5 equiv) and FeBr_3_ (3.6 mg, 0.012 mmol, 0.1 equiv) to obtain 20 mg of tetrahydroazepine **7i** as a pale yellow (0.072 mmol, 60%). *R*_f_ = 0.39 (*n*-hexane/EtOAc 70:30); ^1^H NMR (CDCl_3_, 500 MHz): δ = 7.28 (m, 2H), 7.19 (m, 3H), 5.79 (m, 1H), 5.70 (m, 1H), 4.04 (dq, *J* = 6.9 & 4.1 Hz, 1H), 3.74 (dt, *J* = 14.8 & 4.2 Hz, 1 Hz), 3.29 (ddd, *J* = 14.7, 11.5 & 3.0 Hz, 1H), 2.86 (s, 3H), 2.61 (m, 2H), 2.51 (m, 2H), 2.39 (m, 1H), 2.36–2.28 (m, 1H), 2.01 (m, 1H), 1.86 (m, 1H); ^13^C{^1^H}-NMR (CDCl_3_, 125 MHz): δ = 141.4 (C), 130.7 (CH), 128.4 (2× CH), 128.2 (2× CH), 126.6 (CH), 126.0 (CH), 56.0 (CH), 40.9 (CH_2_), 40.4 (CH_3_), 34.3 (CH_2_), 32.5 (CH_2_), 30.6 (CH_2_); HRMS (ESI^+^): *m*/*z* [*M* + Na]^+^ calcd for C_15_H_21_NO_2_NaS, 302.1191; found, 302.1193.

#### 2-Cyclohexyl-1-(methylsulfonyl)-2,3,6,7-tetrahydro-1*H*-azepine (**7j**)

Following the general procedure (1), to a solution of amine **6b** (50 mg, 0.12 mmol, 1.0 equiv) in 1.2 mL of dry DCM (0.1 M) at 0 °C were added cyclohexanecarboxaldehyde (36 μL, 0.30 mmol, 2.5 equiv) and FeBr_3_ (3.6 mg, 0.012 mmol, 0.1 equiv) to obtain 16 mg of tetrahydroazepine **7j** as a pale yellow oil (0.064 mmol, 53%). *R*_f_ = 0.56 (*n*-hexane/EtOAc 70:30); ^1^H NMR (CDCl_3_, 500 MHz): δ = 5.82 (m, 1H), 5.72 (m, 1H), 3.72–3.66 (dt, *J* = 15.1 & 3.9 Hz, 1H), 3.65–3.60 (m, 1H), 3.09 (ddd, *J* = 14.6, 11.9 & 2.5 Hz, 1H), 2.88 (s, 3H), 2.53–2.44 (m, 2H), 2.44–2.38 (m, 1H), 2.25 (m, 1H), 1.78–1.63 (m, 6H), 1.16 (m, 3H), 0.92 (m, 2H); ^13^C{^1^H}-NMR (CDCl_3_, 125 MHz): δ = 131.1 (CH), 127.5 (CH), 60.8 (CH), 41.3 (CH_2_), 40.7 (CH_3_), 37.2 (CH), 30.8 (CH_2_), 30.6 (CH_2_), 30.1 (CH_2_), 29.3 (CH_2_), 26.2 (CH_2_), 26.1 (CH_2_), 26.0 (CH_2_); HRMS (ESI^+^): *m*/*z* [M + Na]^+^ calcd for C_13_H_23_NO_2_NaS, 280.1347; found, 280.1348.

#### 3-(1-(Methylsulfonyl)-2,3,6,7-tetrahydro-1*H*-azepine-2-yl)propyl benzoate (**7k**)

Following the general procedure (1), to a solution of amine **6b** (50 mg, 0.12 mmol, 1.0 equiv) in 1.2 mL of dry DCM (0.1 M) at 0 °C were added aldehyde 4-oxobutyl benzoate (34 mg, 0.18 mmol, 1.5 equiv), synthesized following the procedure described in the literature,^35^ and FeBr_3_ (3.6 mg, 0.012 mmol, 0.1 equiv) to obtain 25 mg of tetrahydroazepine **7k** as a pale yellow oil (0.074 mmol, 62%). *R*_f_ = 0.29 (*n*-hexane/EtOAc 70:30); ^1^H NMR (CDCl_3_, 400 MHz): δ = 8.03 (m, 2H), 7.56 (m, 1H), 7.44 (m, 2H), 5.77 (m, 1H), 5.68 (m, 1H), 4.33 (m, 2H), 4.03 (m, 1H), 3.72 (dt, *J* = 15.0 & 4.3 Hz, 1H), 3.23 (ddd, *J* = 14.8, 11.6 & 3.2 Hz, 1H), 2.88 (s, 3H), 2.55–2.43 (m, 2H), 2.41–2.27 (m, 2H), 1.89–1.73 (m, 3H), 1.71–1.60 (m, 1H); ^13^C{^1^H}-NMR (CDCl_3_, 100 MHz): δ = 166.5 (C), 132.9 (CH), 130.6 (CH), 130.2 (C), 129.5 (2× CH), 128.3 (2× CH), 126.5 (CH), 64.5 (CH_2_), 56.0 (CH), 40.8 (CH_2_), 40.4 (CH_3_), 32.5 (CH_2_), 30.4 (CH_2_), 29.2 (CH_2_), 25.5 (CH_2_); HRMS (ESI^+^): *m*/*z* [M + Na]^+^ calcd for C_17_H_23_NO_4_NaS, 360.1245; found, 360.1241.

#### 2-Isobutyl-6-methyl-1-tosyl-2,3,6,7-tetrahydro-1*H*-azepine (**10a**)

Following the general procedure (2), to a solution of amine **9a** (50 mg, 0.098 mmol, 1.0 equiv) in 1.0 mL of dry DCM (0.1 M) were added isovaleraldehyde (16 μL, 0.15 mmol, 1.5 equiv) and FeCl_3_ (21 mg, 0.13 mmol, 1.3 equiv) to afford 22 mg of tetrahydroazepine **10a** as a pale yellow oil (0.071 mmol, 72%). *R*_f_ = 0.53 (*n*-hexane/EtOAc 90:10); ^1^H NMR (CDCl_3_, 400 MHz) (*trans*/*cis* diastereomeric mixture 1:0.6): δ = 7.67 (m, 3H), 7.26 (m, 3H), 5.59–5.33 (m, 3H), 4.10 (m, 0.6H), 3.98 (m, 1H), 3.59 (dd, *J* = 14.6 & 2.5 Hz, 0.6H), 3.42 (dd, *J* = 13.3 & 2.0 Hz, 1H), 3.27 (dd, *J* = 13.3 & 7.3 Hz, 1H), 2.82 (dd, *J* = 14.7 & 10.8 Hz, 0.6H), 2.51 (m, 2H), 2.40 (s, 5H), 2.28 (m, 1H), 2.16–2.09 (m, 0.6H), 2.08–1.99 (ddd, *J* = 16.7, 8.4 & 4.8 Hz, 1H), 1.57 (m, 2H), 1.38 (m, 2H), 1.29 (m, 1H), 1.22 (m, 1H), 1.05 (d, *J* = 7.2 Hz, 3H), 0.96 (d, *J* = 7.2 Hz, 2H), 0.80 (m, 9H); ^13^C{^1^H}-NMR (CDCl_3_, 100 MHz) (diastereomeric mixture 1:0.6): δ = 142.8 (C), 142.7 (C), 139.0 (C), 138.1 (CH), 137.2 (C), 136.3 (CH), 129.5 (2× CH), 129.4 (2× CH), 127.1 (4× CH), 125.6 (CH), 124.2 (CH), 54.2 (CH), 52.5 (CH), 48.9 (CH_2_), 46.9 (CH_2_), 41.4 (CH_2_), 39.4 (CH_2_), 35.6 (CH), 35.2 (CH), 33.1 (CH_2_), 29.6 (CH_2_), 24.8 (CH), 24.5 (CH), 23.5 (2× CH_3_), 22.8 (CH_3_), 22.3 (CH_3_), 21.6 (CH_3_), 21.4 (CH_3_), 19.1 (CH_3_), 18.8 (CH_3_); HRMS (ESI^+^): *m*/*z* [M + Na]^+^ calcd for C_18_H_27_NO_2_NaS, 344.1660; found, 344.1656.

#### 2,3-Trans-3,4-cis-2,4-dimethyl-1-tosyl-3-(triphenylsilyl) pyrrolidine (**rac-11**)

A solution of 50 mg of amine **9a** (0.098 mmol, 1.0 equiv) in 1.0 mL of dry DCM (0.1 M) at 10 °C in the presence of 4.7 mg of FeCl_3_ (0.029 mmol, 0.30 equiv) afforded 9.2 mg of pyrrolidine ***rac-11*** (0.018 mmol, 18% yield) as a pale yellow oil. *R*_f_ = 0.36 (*n*-hexane/EtOAc 80:20); ^1^H NMR (CDCl_3_, 500 MHz): δ = 7.57 (d, *J* = 8.2 Hz, 2H), 7.49 (m, 6H), 7.41 (m, 3H), 7.34 (m, 6H), 7.17 (d, *J* = 7.9 Hz, 2H), 3.81 (dt, *J* = 10.6 & 5.9 Hz, 1H), 3.52 (dd, *J* = 11.0 & 5.4 Hz, 1H), 3.17 (d, *J* = 10.8 Hz, 1H), 2.53 (m, 1H), 2.38 (s, 3H), 2.19 (dd, *J* = 10.4 & 5.8 Hz, 1H), 1.28 (d, *J* = 6.0 Hz, 3H), 0.19 (d, *J* = 7.1 Hz, 3H); ^13^C{^1^H}-NMR (CDCl_3_, 100 MHz): δ = 142.8 (C), 135.9 (6× CH), 135.8 (C), 134.1 (3× C), 129.6 (2× CH), 129.4 (2× CH), 128.0 (7× CH), 127.3 (2× CH), 58.2 (CH), 57.1 (CH_2_), 38.7 (CH), 35.4 (CH), 23.4 (CH_3_), 21.5 (CH_3_), 17.7 (CH_3_); HRMS (ESI^+^): *m*/*z* [M + Na]^+^ calcd for C_31_H_33_NO_2_NaSSi, 534.1899; found, 534.1901.

#### 2-Hexyl-6-methyl-1-tosyl-2,3,6,7-tetrahydro-1*H*-azepine (**10b**)

Following the general procedure (2), to a solution of amine **9a** (50 mg, 0.098 mmol, 1.0 equiv) in 1.0 mL of dry DCM (0.1 M) were added heptanal (21 μL, 0.15 mmol, 1.5 equiv) and FeCl_3_ (21 mg, 0.13 mmol, 1.3 equiv) to afford 21 mg of tetrahydroazepine **10b** as a pale yellow oil (0.060 mmol, 61%). *R*_f_ = 0.71 (*n*-hexane/EtOAc 80:20); ^1^H NMR (CDCl_3_, 500 MHz) (*trans*/*cis* diastereomeric mixture 1:0.5): δ = 7.62 (dd, *J* = 10.3 & 8.3 Hz, 3H), 7.27 (m, 3 H), 5.57 (ddd, *J* = 11.2, 4.4 & 2.8 Hz, 1H), 5.50 (m, 0.5H), 5.40 (m, 1.5H), 4.00 (m, 0.5H), 3.90 (m, 1H), 3.61 (dd, *J* = 14.7 & 3.5 Hz, 0.5H), 3.40 (dd, *J* = 13.4 & 2.5 Hz, 1H), 3.28 (dd, *J* = 13.4 & 7.5 Hz, 1H), 2.81 (dd, *J* = 14.6 & 10.6 Hz, 0.5H), 2.53 (m, 1.5H), 2.41 (m, 5.5H), 2.28 (dt, *J* = 16.7 & 2.9 Hz, 1H), 2.18 (ddd, *J* = 15.3, 7.8 & 5.9 Hz 0.5H), 2.08 (ddd, *J* = 16.7, 8.4 & 4.6 Hz 1H), 1.56–1.38 (m, 4H), 1.28–1.10 (m, 11H), 1.06 (d, *J* = 7.2 Hz, 3H), 1.04–0.99 (m, 2H), 0.98 (d, *J* = 7.2 Hz, 1.5H), 0.85 (dt, *J* = 7.1 & 2.3 Hz, 4.5H); ^13^C{^1^H}-NMR (CDCl_3_, 100 MHz) (diastereomeric mixture 1:0.5): δ = 142.8 (C), 142.7 (C), 139.0 (CH), 138.0 (C), 137.4 (CH), 136.4 (CH), 129.5 (2× CH), 129.4 (2× CH), 127.0 (4× CH), 125.5 (CH), 124.1 (CH), 56.4 (CH), 54.7 (CH), 48.9 (CH_2_), 47.1 (CH_2_), 35.9 (CH), 35.2 (CH), 33.1 (CH_2_), 32.6 (CH_2_), 31.7 (2× CH_2_), 30.6 (CH_2_), 29.8 (CH_2_), 29.1 (CH_2_), 29.0 (CH_2_), 26.4 (CH_2_), 26.0 (CH_2_), 22.5 (2× CH_2_), 21.4 (2× CH_3_), 19.1 (CH_3_), 18.9 (CH_3_), 14.0 (2× CH_3_); HRMS (ESI^+^): *m*/*z* [*M* + Na]^+^ calcd for C_20_H_31_NO_2_NaS: 372.1973, found, 372.1974.

#### 2-(But-3-en-1-yl)-6-methyl-1-tosyl-2,3,6,7-tetrahydro-1*H*-azepine (**10c**)

Following the general procedure (2), to a solution of amine **9a** (50 mg, 0.098 mmol, 1.0 equiv) in 1.0 mL of dry DCM (0.1 M) were added 4-pentenal (21 μL, 0.15 mmol, 1.5 equiv) and FeCl_3_ (21 mg, 0.13 mmol, 1.3 equiv) to afford 17 mg of tetrahydroazepine **10c** as a pale yellow oil (0.052 mmol, 53%). *R*_f_ = 0.70 (*n*-hexane/EtOAc 80:20); ^1^H NMR (CDCl_3_, 500 MHz) (*trans*/*cis* diastereomeric mixture 1:0.7): δ = 7.69 (dd, *J* = 13.0 & 8.2 Hz, 3H), 7.31–7.26 (m, 3H), 5.70 (m, 1.7H), 5.58–5.53 (dt, *J* = 7.7 & 3.5 Hz, 0.7H), 5.52–5.47 (m, 1H), 5.44–5.36 (m, 1.6H), 5.00–4.90 (m, 3H), 4.06 (m, 1H), 3.94 (m, 0.7H), 3.63 (dd, *J* = 14.9 & 3.5 Hz, 1H), 3.45 (dd, *J* = 13.2 & 2.4 Hz, 0.7H), 3.23 (dd, *J* = 13.3 & 7.9 Hz, 0.7H), 2.84 (dd, *J* = 14.9 & 10.8 Hz, 1H), 2.53 (m, 1H), 2.45–2.37 (m, 5H), 2.24–2.17 (m, 1H), 2.07 (ddd, *J* = 16.9, 8.5 & 4.6 Hz, 1H), 1.95–1.84 (m, 3H), 1.70–1.59 (m, 2H), 1.58–1.54 (m, 2H), 1.27 (m, 3H), 1.05 (d, *J* = 7.4 Hz, 2H), 0.98 (d, *J* = 7.4 Hz, 3H); ^13^C{^1^H}-NMR (CDCl_3_, 125 MHz) (diastereomeric mixture 1:0.7): δ = 142.9 (C), 142.8 (C), 138.9 (C), 138.1 (CH), 137.9 (CH), 137.8 (CH), 137.0 (C), 136.6 (CH), 129.6 (2× CH), 129.4 (2× CH), 127.0 (4× CH), 125.2 (CH), 123.8 (CH), 114.9 (CH_2_), 114.8 (CH_2_), 56.0 (CH), 54.3 (CH), 49.4 (CH_2_), 47.1 (CH_2_), 35.7 (CH), 35.1 (CH), 32.6 (CH_2_), 32.1 (CH_2_), 30.6 (CH_2_), 30.1 (CH_2_), 29.9 (CH_2_), 29.1 (CH_2_), 21.5 (2× CH_3_), 19.2 (CH_3_), 19.1 (CH_3_); HRMS (ESI^+^): *m*/*z* [M + Na]^+^ calcd for C_18_H_25_NO_2_NaS, 342.1504; found, 342.1510.

#### 6-Allyl-2-isobutyl-1-tosyl-2,3,6,7-tetrahydro-1*H*-azepine (**10d**)

Following the general procedure 3.6, to a solution of amine **9b** (40 mg, 0.074 mmol, 1.0 equiv) in 0.7 mL of dry DCM (0.1 M) were added isovaleraldehyde (12 μL, 0.11 mmol, 1.5 equiv) and FeCl_3_ (16 mg, 0.096 mmol, 1.3 equiv) to afford 15 mg of tetrahydroazepine **10d** as a pale yellow oil (0.044 mmol, 60%). *R*_f_ = 0.58 (*n*-hexane/EtOAc 90:10); ^1^H NMR (CDCl_3_, 500 MHz) (*trans*/*cis* diastereomeric mixture 1:0.6): δ = 7.68 (dd, *J* = 11.7 & 8.2 Hz, 3H), 7.27 (m, 3H), 5.75 (m, 1.6H), 5.62–5.43 (m, 3H), 5.05 (m, 3H), 4.14 (m, 1H), 4.00 (m, 0.6H), 3.66 (dd, *J* = 14.8 & 3.2 Hz, 1H), 3.47 (dd, *J* = 13.4 & 2.7 Hz, 0.6H), 3.33 (dd, *J* = 13.5 & 7.4 Hz, 0.6H), 2.83 (dd, *J* = 14.9 & 10.8 Hz, 1H), 2.48 (m, 1.6H), 2.42 (m, 5H), 2.21 (m, 1H), 2.18–2.10 (m, 1H), 2.10–2.07 (m, 2H), 1.41–1.20 (m, 9H), 0.81 (brt, *J* = 6.6 Hz, 9H); ^13^C{^1^H}-NMR (CDCl_3_, 100 MHz) (diastereomeric mixture 1:0.6): δ = 142.8 (C), 138.9 (C), 137.3 (C), 136.2 (C), 136.0 (CH), 135.7 (2× CH), 134.6 (CH), 129.5 (2× CH),129.4 (2× CH), 127.2 (2× CH), 127.1 (2× CH), 126.3 (CH), 125.2 (CH), 117.1 (CH_2_), 116.7 (CH_2_), 54.2 (CH), 52.5 (CH), 47.3 (CH_2_), 45.0 (CH_2_), 41.5 (CH_2_), 40.0 (CH), 39.9 (CH), 39.6 (CH_2_) 37.9 (CH_2_), 37.7 (CH_2_), 33.0 (CH_2_), 29.7 (CH_2_), 24.8 (CH), 24.5 (CH), 23.6 (CH_3_), 22.8 (2× CH_3_), 22.3 (CH_3_), 21.6 (CH_3_), 21.5 (CH_3_); HRMS (ESI^+^): *m*/*z* [M + Na]^+^ calcd for C_20_H_29_NO_2_NaS, 370.1817; found, 370.1818.

## Supporting Information Available

Experimental details and procedures, compound characterization data, and NMR spectra (PDF)
