# Supplementary material for: Synthesis of Tetrahydroazepines through Silyl Aza-Prins Cyclization Mediated by Iron(III) Salts
Source: J Org Chem. 2022 Aug 17;87(17):11735–42. doi: 10.1021/acs.joc.2c01396 (PMC9442639; doi:10.1021/acs.joc.2c01396)
Supplement: Supplementary file 1 — jo2c01396_si_001.pdf [file jo2c01396_si_001.pdf]

Supporting Information

Synthesis of Tetrahydroazepines through Silyl Aza-Prins Cyclization Mediated by Iron(III) Salts

Victoria Sinka,*[a] Israel Fernández,[b] and Juan I. Padrón*[a]

[a] Instituto de Productos Naturales y Agrobiología (IPNA), CSIC, 38206. Avda. Astrofísico Fco. Sánchez 3, 38206 La Laguna, Tenerife (Spain). [b] Departamento de Química Orgánica and Centro de Innovación en Química Avanzada (ORFEO-CINQA), Facultad de Ciencias Químicas, Universidad Complutense de Madrid, 28040-Madrid (Spain).

Table of contents

1. Supporting schemes and tables S2
1.1. Synthesis of 1-amino-3-triphenylsilyl-4-pentenes **6a-b**. S2
1.2. Catalyst optimization for SAPC of 1-amino-3-triphenylsilyl-4-pentene **6a**. S2
1.3. Synthesis of 1-amino-3-triphenylsilyl-4-pentenes 9a-b substituted at β position S2
1.4. Catalyst optimization for SAPC of 1-amino-3-triphenylsilyl-4-pentene **9a**. S3
1.5. NMR study for the diastereoselectivity determination S3
2. Experimental Procedures and Compound Data S4
2.1. General procedure for tosylation of alcohols S4
2.2. General procedure for substitution of tosylate by sulfonylamide S4
2.3. General procedure for α-alkylation of esters S4
2.4. General procedure for reduction of esters to alcohols S4
2.5. Compound data S4
3. NMR spectra S11
4. Computational details S44
4.1. Cartesian coordinates S44
5. References S52

1. Supporting schemes and tables

1.1. Synthesis of 1-amino-3-triphenylsilyl-4-pentenes **6a-b**.

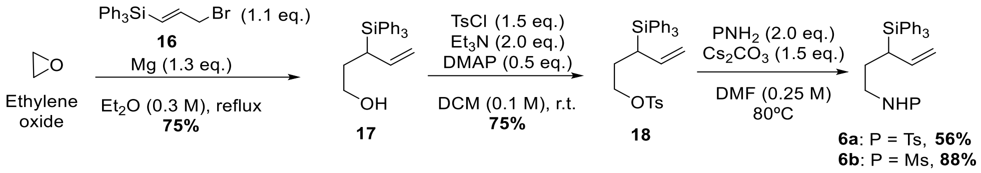

Scheme S1. Synthetic route for 1-amino-3-triphenylsilyl-4-pentenes **6a-b**.

1.2. Catalyst optimization for SAPC of 1-amino-3-triphenylsilyl-4-pentene **6a**.

Table S1. Catalyst optimization reaction conditions.

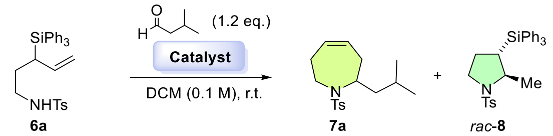

Entry	Catalyst (equiv.)	Time (min)	Yield 7a:8 (%)[a]	Conversion (%)
1	$FeBr_3$ (0.3)	1	67:10	100
2	$FeCl_3$ (0.1)	50	55:19	100
3	$Fe(acac)_3$ (0.1)/ TMSCl (1.0)	30	58:21	100
4	$Fe(OTf)_3$ (0.1)	55	48:32	100
5	$FeBr_2$ (0.1)	240	20:0	53
6	$FeBr_2$ (1.0)	270	33:14	78
7	CuCl (0.1)	200	N.R.	N.R.
8	$InBr_3$ (0.1)	240	48:10	86
9	$InCl_3$ (0.1)	240	13:0	23

[a] Reaction conditions: **6a** (0.10 mmol), isovaleraldehyde (0.12 mmol), DCM (0.1 M). Isolated yield.

1.3. Synthesis of 1-amino-3-triphenylsilyl-4-pentenes **9a-b** substituted at β position

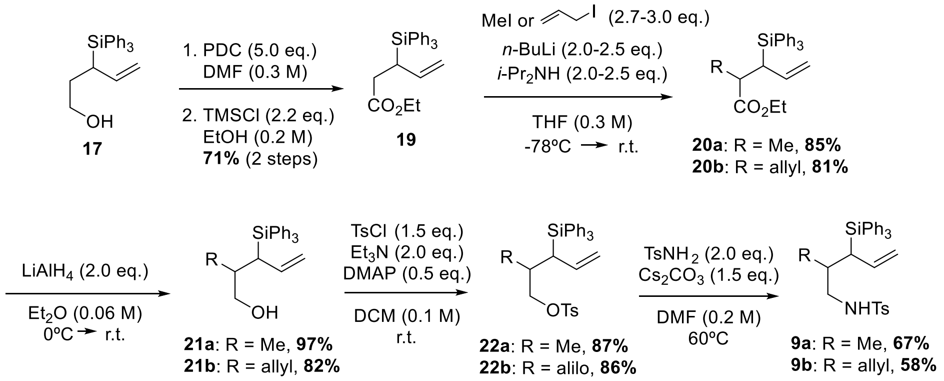

Scheme S2. Synthetic route for 1-amino-3-triphenylsilyl-4-pentenes **9a-b**.

1.4. Catalyst optimization for SAPC of 1-amino-3-triphenylsilyl-4-pentene **9a**.

Table S2. Catalyst optimization reaction conditions.

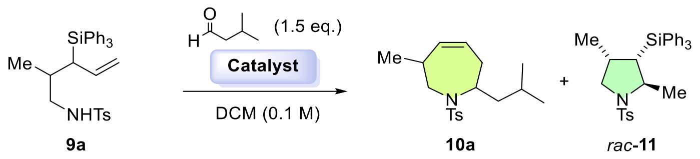

Entry	Catalyst (equiv.)	Temperature(ºC)	Yield 10a:11 (%)[a]	Conversion (%)
1	$FeBr_3$ (0.3)[b]	-20	N. R.	N.R.
2	$FeBr_3$ (0.3)[b]	0	22:21	69
3	$FeBr_3$ (0.3)	10	43:11	93
4	$FeCl_3$ (0.3)	10	45:18	100
5	$InCl_3$ (0.3)	10	7:0	27
6	$InBr_3$ (0.3)	10	11:6	38
7	$Fe(acac)_3$ (0.3)/ TMSCl (1.0)	10	33:17	100
8	$Fe(OTf)_3$ (0.3)	10	0:50	100

[a] Reaction conditions: **9a** (0.098 mmol), isovaleraldehyde (0.15 mmol), DCM (0.1 M). Isolated yield. [b]After 3 hours of reaction, another 0.3 equivalents of $FeBr_3$ were added.

1.5. NMR study for the diastereoselectivity determination

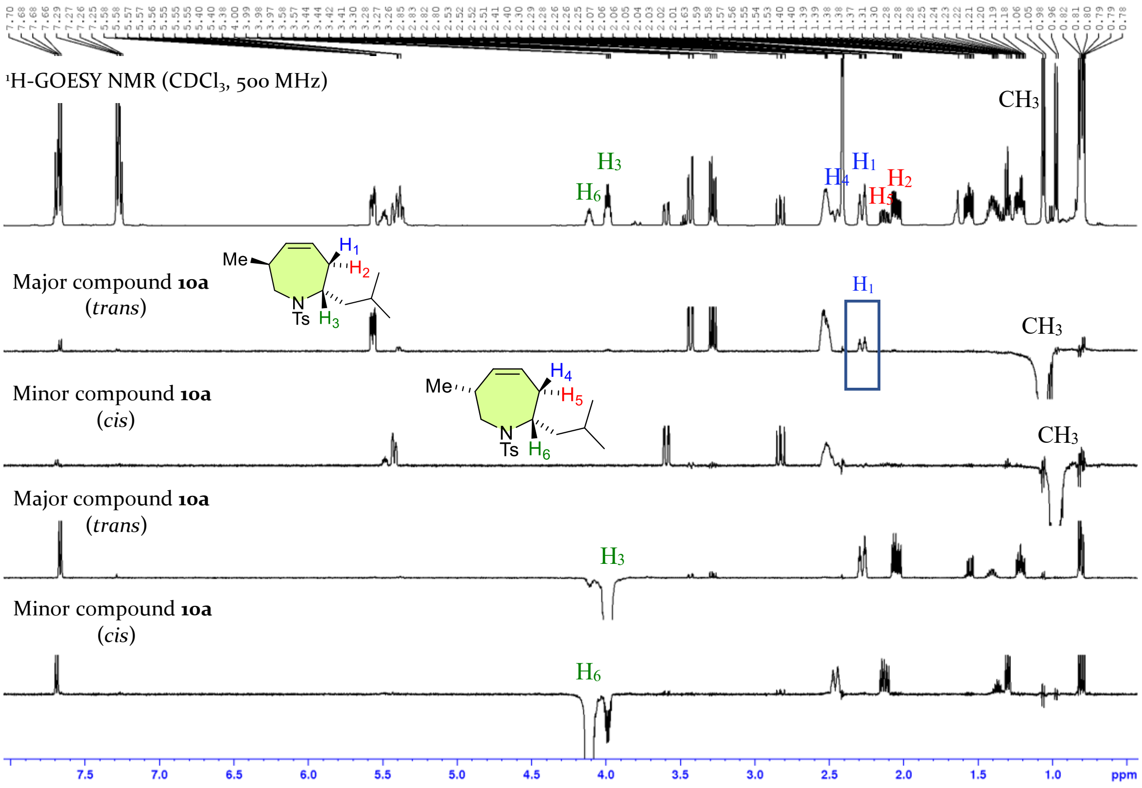

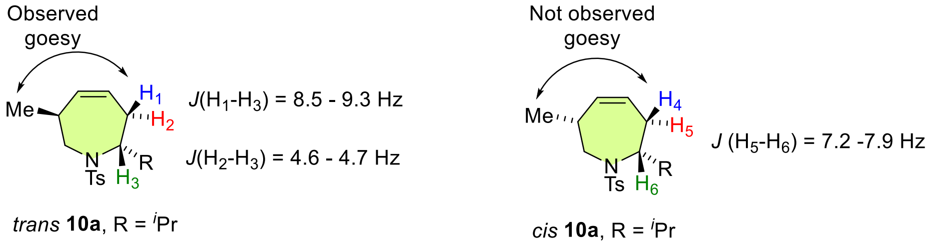

2. Experimental Procedures and Compound Data

2.1. General procedure for tosylation of alcohols

To a solution of corresponding alcohol (1.0 equiv.) in dry DCM (0.1 M) at room temperature under inert atmosphere were added Et_3N (2.0 equiv.), TsCl (1.5 equiv.) and a small amount of DMAP. Once the reaction was complete, the solvent was removed under reduced pressure and the residue was purified by flash silica gel column chromatography (*n*-hexane/EtOAc solvent system).

2.2. General procedure for substitution of tosylate by sulfonylamide

To a solution of corresponding tosylate (1.0 equiv.) in dry DMF (0.25 M) at room temperature under inert atmosphere were added $TsNH_2$ or $MsNH_2$ (2.0 equiv.) and Cs_2CO_3 (1.5 equiv.). The reaction mixture was warmed to 80 ºC until the starting material was completely consumed. Then, it was allowed to reach room temperature and the reaction mixture was filtered through a pad of silica gel. The solvent was removed under reduced pressure and the residue was purified by flash silica gel column chromatography (*n*-hexane/EtOAc solvent system).

2.3. General procedure for α-alkylation of esters

Following the procedure described by Panek and coworkers,[1] to a solution of *i*-Pr_2NH (2.0 equiv.) in dry THF (0.94 M *vs* *i*-Pr_2NH) at -78 ºC under inert atmosphere was added *n*-BuLi (2.0 equiv.). The mixture was stirred at 0 ºC for 10 min. and then cooled to -78 ºC. Next, a solution of the ester (1.0 equiv.) in dry THF (2.35 M) was added dropwise. This mixture was stirred at -78 ºC for 30 min. and alkyl iodide (2.7 equiv.) in dry THF (4.7 M) was added. The reaction mixture was allowed to reach room temperature and stirred overnight. It was quenched with saturated aqueous NH_4Cl. The phases were separated and the aqueous layer was extracted with 3 x EtOAc. The combined organic phases were dried over anhydrous $MgSO_4$, filtrated, and concentrated under reduced pressure. The residue was purified by flash silica gel column chromatography (*n*-hexane/EtOAc solvent system).

2.4. General procedure for reduction of esters to alcohols

To a solution of ester (1.0 equiv.) in dry Et_2O (0.06 M) at 0 ºC under inert atmosphere was added $LiAlH_4$ (2.0 equiv). Then, the ice-bath was removed and the mixture was stirred at room temperature. Once the reaction was completed, saturated aqueous K_2CO_3 solution and water were added. The solid was filtrated and the organic phase was concentrated under reduced pressure. The residue was purified by flash silica gel column chromatography (*n*-hexane/EtOAc solvent system).

2.5. Compound data

(*E*)-(3-bromoprop-1-en-1-yl)triphenylsilane (16)

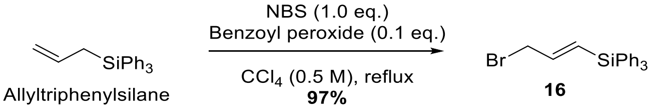

Following the procedure described by Corriu,[2,3] to a solution of commercially available allyltriphenylsilane (6.00 g, 20.0 mmol, 1.0 equiv.) in tetrachloromethane (CCl_4, 40.0 mL, 0.5 M) at

room temperature, were added *N*-bromosuccinimide (NBS, 3.56 g, 20.0 mmol, 1.0 equiv.) and benzoyl peroxide (0.485 g, 2.00 mmol, 0.1 equiv.). The reaction mixture was heated to reflux and monitored by TLC (Heat-On™ Block System). Then, it was allowed to cool to room temperature, and filtered through a short plug of Celite. The solvent was removed under reduced pressure and the reaction mixture was purified by flash silica gel column chromatography (*n*-hexane/EtOAc 95:5 solvent system) to afford 7.36 g of allyl bromide **16** as a white amorphous solid (19.4 mmol, 97% yield).

R$_f$ = 0.55 (*n*-hexane/EtOAc 95:5); **^{1}H-NMR ($CDCl_3$, 400 MHz)** δ = 7.51 (m, 6H), 7.46-7.35 (m, 9H), 6.50 (d, *J* = 18.1 Hz, 1H), 6.27 (dt, *J* = 18.2 & 6.7 Hz, 1H), 4.06 (dd, *J* = 6.7 & 0.6 Hz, 2H); **$^{13}C\{^1H\}$-NMR ($CDCl_3$, 125 MHz)** δ = 146.0 (CH), 135.9 (6 x CH), 133.7 (3 x C), 129.7 (3 x CH), 129.6 (CH), 128.0 (6 x CH), 34.7 (CH_2); **HRMS (ESI+):** *m/z* [*M*+Na]$^+$ calcd. for $C_{21}H_{19}BrNaSi$: 401.0337; found: 401.0341.

3-(triphenylsilyl)pent-4-en-1-ol (17)

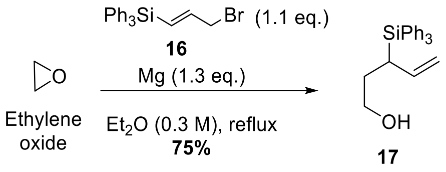

To a solution of Mg (0.380 g, 15.6 mmol, 1.3 equiv.) in 40 mL of dry Et_2O (0.3 M) at room temperature was added allyl-TPS bromide **16** (5.00 g, 13.2 mmol, 1.1 equiv.) dissolved in 13 mL of dry Et_2O (1.0 M). The reaction mixture was heated to reflux for 2 h (Heat-On™ Block System). Then, ethylene oxide 2.5-3.3 M in THF (4.8 mL, 12.0 mmol, 1.0 equiv.) was added dropwise at the refluxing temperature. At the same time, 8 mL of dry DCM was added dropwise, to dissolve the alcohol and avoid the formation of an insoluble gel to afford 3.10 g of *bis*-homoallylic alcohol **17** as pale yellow oil (9.0 mmol, 75% yield).

R$_f$ = 0.40 (*n*-hexane/EtOAc 80:20); **^{1}H-NMR ($CDCl_3$, 500 MHz)** δ = 7.57 (m, 6H), 7.44-7.39 (m, 3H), 7.39-7.33 (m, 6H), 5.77 (dt, *J* = 10.0 & 17.0 Hz, 1H), 4.99 (m, 2H), 3.72 (m, 1H), 3.67 (m, 1H), 2.71 (m, 1H), 2.01 (m, 1H), 1.74 (m, 1H), 1.29 (brt, *J* = 5.5 Hz, 1H); **$^{13}C\{^1H\}$-NMR ($CDCl_3$, 125 MHz)** δ = 138.4 (CH), 136.1 (6 x CH), 133.6 (3 x C), 129.5 (3 x CH), 127.8 (6 x CH), 115.2 (CH_2), 62.1 (CH_2), 32.0 (CH_2), 28.5 (CH); **HRMS (ESI$^+$)**: *m/z* [*M*+Na]$^+$ calcd. for $C_{23}H_{24}ONaSi$: 367.1494; found: 367.1501.

3-(triphenylsilyl)pent-4-en-1-yl 4-methylbenzenesulfonate (18)

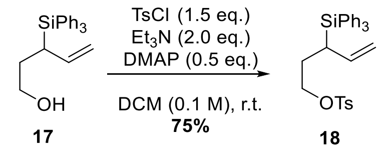

Following the general procedure 2.1, alcohol **17** (2.00 g, 5.81 mmol, 1.0 equiv.) was dissolved in 58 mL of dry DCM (0.1 M) and Et_3N (1.6 mL, 11.6 mmol, 2.0 equiv.), TsCl (1.66 g, 8.72 mmol, 1.5 equiv.) and DMAP (0.355 g, 2.90 mmol, 0.5 equiv.) were added to give 2.17 g of tosylate **18** as a white amorphous solid (4.36 mmol, 75% yield).

R$_f$ = 0.51 (*n*-hexane/EtOAc 80:20); **^{1}H-NMR ($CDCl_3$, 400 MHz)** δ = 7.75 (m, 2H), 7.52 (m, 6H), 7.44-7.39 (m, 3H), 7.39-7.32 (m, 6H), 7.32-7.28 (m, 2H), 5.56 (m, 1H), 4.92 (d, *J* = 10.3 Hz, 1H), 4.80 (d, *J* = 16.9 Hz, 1H), 4.05 (m, 2H), 2.60 (brt, *J* = 10.9 Hz, 1H), 2.44 (s, 3H), 2.14 (m, 1H), 1.67 (m, 1H); **$^{13}C\{^1H\}$-NMR ($CDCl_3$, 100 MHz)** δ = 144.5 (C), 136.6 (CH), 136.0 (6 x CH), 133.2 (C), 133.1 (3 x C), 129.7 (3 x CH), 129.6 (4 x CH), 127.8 (6 x CH), 116.1 (CH_2), 69.2 (CH_2), 28.4 (CH_2), 27.5 (CH), 21.6 (CH_3). **HRMS (ESI$^+$):** *m/z* [*M*+Na]$^+$ calcd. for $C_{30}H_{30}O_3NaSiS$: 521.1583; found: 521.1585.

4-methyl-*N*-(3-(triphenylsilyl)pent-4-en-1-yl)benzenesulfonamide (6a)

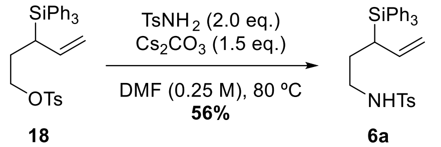

Following the general procedure 2.2, to a solution of tosylate **18** (1.00 g, 2.00 mmol, 1.0 equiv.) in 8.0 mL of dry DMF (0.25 M) were added $TsNH_2$ (0.685 g, 4.00 mmol, 2.0 equiv.) and Cs_2CO_3 (0.977 g, 3.00 mmol, 1.5 equiv.) to obtain 0.627 g of tosylamide **6a** as a white amorphous solid (1.26 mmol, 73% yield).

R_f = 0.34 (*n*-hexane/EtOAc 80:20); **^{1}H-NMR ($CDCl_3$, 400 MHz)** δ = 7.67 (brd, *J* = 8.3 Hz, 2H), 7.49 (m, 6H), 7.41 (m, 3H), 7.35 (m, 6H), 7.24 (brd, *J* = 7.9 Hz, 2H), 5.61 (dt, *J* = 10.0 & 17.1 Hz, 1H), 4.95 (dd, *J* = 1.2 & 10.2 Hz, 1H), 4.86 (brd, *J* = 17.1 Hz, 1H), 4.25 (t, *J* = 6.3 Hz, 1H), 3.04 (m, 1H), 2.95 (m, 1H), 2.50 (m, 1H), 2.41 (s, 3H), 1.87 (m, 1H), 1.56 (m, 1H); **^{13}C{^{1}H}-NMR ($CDCl_3$, 100 MHz)** δ = 143.3 (C), 137.5 (CH), 137.1 (C), 136.1 (6 x CH), 133.3 (3 x C), 129.6 (5 x CH), 127.9 (6 x CH), 127.1 (2 x CH), 115.8 (CH_2), 42.6 (CH_2), 29.4 (CH), 29.2 (CH_2), 21.5 (CH_3); **HRMS (ESI$^+$):** *m/z* [*M*+Na]$^+$ calcd. for $C_{30}H_{31}NO_2NaSiS$: 520.1742; found: 520.1744.

N-(3-(triphenylsilyl)pent-4-en-1-yl)methanesulfonamide (6b)

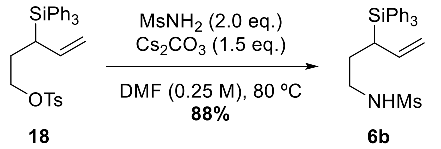

Following the general procedure 2.2, to a solution of tosylate **18** (0.500 g, 1.00 mmol, 1.0 equiv.) in 4.0 mL of dry DMF (0.25 M) were added $MsNH_2$ (0.190 g, 2.00 mmol, 2.0 equiv.) and Cs_2CO_3 (0.488 g, 1.50 mmol, 1.5 equiv.) to obtain 0.371 g of mesylamide **6a** as a white amorphous solid (0.88 mmol, 88% yield).

R_f = 0.51 (*n*-hexane/EtOAc 60:40); **^{1}H-NMR ($CDCl_3$, 400 MHz)** δ = 7.57 (m, 6H), 7.46-7.34 (m, 9H), 5.73 (dt, *J* = 9.9 & 17.1 Hz, 1H), 5.03 (m, 2H), 4.36 (brt, *J* = 6.2 Hz, 1H), 3.24 (m, 1H), 3.10 (m, 1H), 2.86 (s, 3H), 2.63 (m, 1H), 2.00 (m, 1H), 1.74 (m, 1H); **^{13}C{^{1}H}-NMR ($CDCl_3$, 125 MHz)** δ = 137.6 (CH), 136.1 (6 x CH), 133.2 (3 x C), 129.7 (3 x CH), 127.9 (6 x CH), 116.0 (CH_2), 42.6 (CH_2), 40.3 (CH_3), 29.6 (CH_2), 29.5 (CH); **HRMS (ESI$^+$):** *m/z* [M+Na]$^+$ calcd. for $C_{24}H_{27}NO_2NaSiS$: 444.1429; found: 444.1429.

3-(triphenylsilyl)pent-4-enoic acid (precursor of 19)

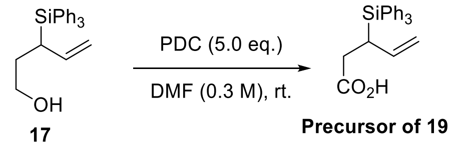

To a solution of *bis*-homoallylic alcohol **17** (0.250 g, 0.73 mmol, 1.0 equiv.) in 2.4 mL of dry DMF (0.3 M) at room temperature under inert atmosphere was added PDC (1.37 g, 3.65 mmol, 5.0 equiv.) and a small amount of $MgSO_4$. The reaction mixture was stirred overnight. Then, it was filtered through a pad of Celite. Water was added and aqueous phase was extracted with 3 x EtOAc. The combined organic layers were dried over anhydrous $MgSO_4$, filtered through a pad of silica, and concentrated under reduced pressure. This crude reaction was used in the next step without further purification.

Ethyl 3-(triphenylsilyl)pent-4-enoate (19)

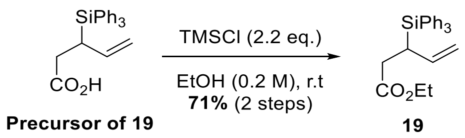

To a solution of the previous carboxylic acid crude (0.56 mmol, 1.0 equiv.) in 2.8 mL of dry EtOH (0.2 M) at room temperature was added TMSCl (0.16 mL, 1.23 mmol, 2.2 equiv.) to afford 155 mg of *bis*-homoallylic ester **19** as a pale yellow oil (0.40 mmol, 71% yield in two steps).

R$_f$ = 0.64 (*n*-hexane/EtOAc 90:10); **^{1}H-NMR (CDCl$_3$, 400 MHz)** δ = 7.58 (dd, *J* = 7.7 & 1.3 Hz, 6H), 7.39 (m, 9H), 5.86 (ddd, *J* = 17.3, 10.5 & 8.1 Hz, 1H), 4.95 (m, 2H), 4.08 (q, *J* = 7.1 Hz, 2H), 3.14 (m, 1H), 2.68 (dd, *J* = 15.7 & 2.8 Hz, 1H), 2.49 (dd, *J* = 15.7 & 12.2 Hz, 1H), 1.20 (t, *J* = 7.1 Hz, 3H); **^{13}C{^{1}H}-NMR (CDCl$_3$, 100 MHz)** δ = 173.0 (C), 137.2 (CH), 136.1 (6 x CH), 133.1 (3 x C), 129.7 (3 x CH), 127.9 (6 x CH), 114.7 (CH$_2$), 60.4 (CH$_2$), 34.5 (CH$_2$), 28.0 (CH), 14.2 (CH$_3$); **HRMS (ESI$^+$):** *m/z* [*M*+Na]$^+$ calcd. for $C_{25}H_{26}O_2NaSi$: 409.1600; found: 409.1603.

Ethyl 2-methyl-3-(triphenylsilyl)pent-4-enoate (20a)

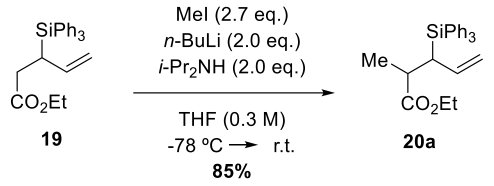

Following the general procedure 2.3, to a solution of *i*-Pr$_2$NH (0.77 mL, 5.46 mmol, 2.0 equiv.) in 6.1 mL of dry THF (0.94 M), were added *n*-BuLi 2.5 M in hexane (2.2 mL, 5.46 mmol, 2.0 equiv.), ester **19** (1.06 g, 2.73 mmol, 1.0 equiv.) in 1.2 mL of dry THF and iodomethane (0.45 mL, 7.37 mmol, 2.7 equiv.) in 1.6 mL of dry THF to obtain 0.941 g of α-methyl ester **20a** as a white amorphous solid (2.32 mmol, 85% yield).

R$_f$ = 0.71 (*n*-hexane/EtOAc 90:10); **^{1}H-NMR (CDCl$_3$, 500 MHz)** δ = 7.61 (m, 6H), 7.44-7.39 (m, 3H), 7.39-7.33 (m, 6H), 5.82 (dt, *J* = 16.9 & 10.4 Hz, 1H), 5.00 (m, 2H), 3.89 (m, 1H), 3.78 (m, 1H), 3.11 (dd, *J* = 10.8 & 5.4 Hz, 1H), 2.93 (m, 1H), 1.15 (t, *J* = 7.1 Hz, 3H), 1.06 (d, *J* = 7.1 Hz, 3H); **^{13}C{^{1}H}-NMR (CDCl$_3$, 125 MHz)** δ = 175.8 (C), 136.4 (6 x CH), 134.9 (CH), 133.7 (3 x C), 129.5 (3 x CH), 127.8 (6 x CH), 117.4 (CH$_2$), 60.2 (CH$_2$), 39.5 (CH), 35.4 (CH), 15.1 (CH$_3$), 14.2 (CH$_3$); **HRMS (ESI$^+$):** *m/z* [*M*+Na]$^+$ calcd. for $C_{26}H_{28}O_2NaSi$: 423.1756; found: 423.1729.

Ethyl 2-allyl-3-(triphenylsilyl)pent-4-enoate (20b)

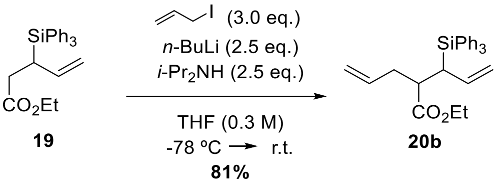

Following the general procedure 2.3, to a solution of *i*-Pr$_2$NH (0.46 mL, 3.23 mmol, 2.5 equiv.) in 3.4 mL of dry THF (0.94 M), were added *n*-BuLi 2.5 M in hexane (1.3 mL, 3.23 mmol, 2.5 equiv.), ester **19** (0.500 g, 1.29 mmol, 1.0 equiv.) in 0.6 mL of dry THF and allyl iodide (0.36 mL, 3.87 mmol, 3.0 equiv.) in 0.8 mL of dry THF to afford 0.443 g of α-allyl ester **20b** as a pale yellow oil (1.04 mmol, 81% yield).

R$_f$ = 0.55 (*n*-hexane/EtOAc 90:10); **^{1}H-NMR (CDCl$_3$, 400 MHz)** δ 7.62 (dd, *J* = 7.8 & 1.3 Hz, 6H), 7.38 (m, 9H), 5.75 (dt, *J* = 16.9 & 10.5 Hz, 1H), 5.60 (ddt, *J* = 17.0, 10.2 & 6.9 Hz, 1H), 5.03 (dd, *J* = 10.1 & 1.5 Hz, 1H), 4.97 (dd, *J* = 17.0 & 1.0 Hz, 1H), 4.90-4.80 (m, 2H), 3.75 (dq, *J* = 10.8 & 7.1 Hz, 1H), 3.56 (dq, *J* = 14.3 & 7.1 Hz, 1H), 3.00 (dd, *J* = 10.8 & 6.7 Hz, 1H), 2.84 (ddd, *J* = 10.9, 6.9 & 3.2 Hz, 1H), 2.33 (m, 1H), 2.16 (m, 1H), 1.09 (t, J = 7.1 Hz, 3H); **^{13}C{^{1}H}-NMR (CDCl$_3$, 100 MHz)** δ 174.3 (C), 136.4 (6 x CH), 135.8 (CH), 135.3 (CH), 133.5 (3 x C), 129.5 (3 x CH), 127.7 (6 x CH), 117.5 (CH$_2$), 116.3 (CH$_2$), 60.0 (CH$_2$), 45.4 (CH), 35.2 (CH), 34.8 (CH$_2$), 14.1 (CH$_3$); **HRMS (ESI$^+$):** *m/z* [M+Na]$^+$ calcd. for $C_{28}H_{30}O_2NaSi$: 449.1913; found: 449.1913.

2-methyl-3-(triphenylsilyl)pent-4-en-1-ol (21a)

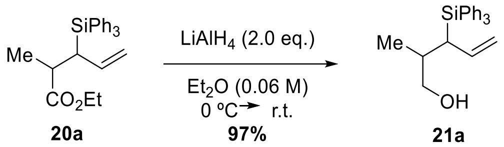

Following the general procedure 2.4, to the solution of α-methyl ester **20a** (1.01 g, 2.53 mmol, 1.0 equiv.) in 42 mL of dry Et_2O (0.06M) was added $LiAlH_4$ (0.192 g, 5.06 mmol, 2.0 equiv.) to give 0.878 g of alcohol **21a** as a white amorphous solid (2.45 mmol, 97% yield).

R_f = 0.37 (*n*-hexane/EtOAc 80:20); **^{1}H-NMR ($CDCl_3$, 500 MHz)** δ = 7.60 (m, 6H), 7.44-7.33 (m, 9H), 5.91 (dt, *J* = 10.7 & 16.5 Hz, 1H), 5.04 (m, 1H), 5.01 (m, 1H), 3.48 (m, 2H), 2.93 (dd, *J* = 2.8 & 11.0 Hz, 1H), 2.22 (m, 1H), 1.32 (brs, 1H), 0.75 (d, *J* = 7.2 Hz, 3H); **^{13}C{^{1}H}-NMR ($CDCl_3$, 125 MHz)** δ = 136.3 (6 x CH), 135.1 (CH), 134.3 (3 x C), 129.4 (3 x CH), 127.8 (6 x CH), 117.2 (CH_2), 67.7 (CH_2), 35.2 (CH), 33.9 (CH), 14.0 (CH_3); **HRMS (ESI$^+$):** *m/z* [*M*+Na]$^+$ calcd. for $C_{24}H_{26}ONaSi$:381.1651; found: 381.1658.

2-allyl-3-(triphenylsilyl)pent-4-en-1-ol (21b)

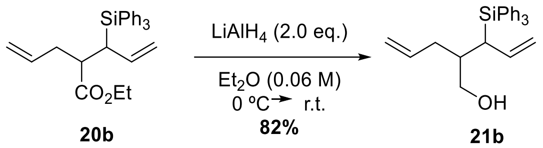

Following the general procedure 2.4, to the solution of α-allyl ester **20b** (0.890 g, 2.09 mmol, 1.0 equiv.) in 35 mL of dry Et_2O (0.06M) was added $LiAlH_4$ (0.158 g, 4.17 mmol, 2.0 equiv.) to obtain 0.657 g of alcohol **21b** as a colorless oil (1.71 mmol, 82% yield).

R_f = 0.38 (*n*-hexane/EtOAc 80:20); **^{1}H-NMR ($CDCl_3$, 400 MHz)** δ = 7.61 (dd, *J* = 7.6 & 1.5 Hz, 6H), 7.38 (m, 9H), 5.89 (m, 1H), 5.59 (m, 1H), 5.05 (m, 2H), 4.88 (d, *J* = 10.1 Hz, 1H), 4.81 (dd, *J* = 17.0 & 1.0 Hz, 1H), 363 (dt, *J* = 10.9 & 4.5 Hz, 1H), 3.48 (dt, *J* = 10.8 & 7.4 Hz, 1H), 3.04 (dd, *J* = 11.1 & 3.1 Hz, 1H), 2.13 (m, 1H), 2.04 (m, 1H), 1.83 (m, 1H), 1.33 (dd, *J* = 7.0 & 4.8 Hz, 1H); **^{13}C{^{1}H}-NMR ($CDCl_3$, 100 MHz)** δ = 137.2 (CH), 136.3 (6 x CH), 135.8 (CH), 134.2 (3 x C), 129.5 (3 x CH), 127.8 (6 x CH), 117.4 (CH_2), 116.0 (CH_2), 65.0 (CH_2), 40.5 (CH), 33.8 (CH), 33.5 (CH_2); **HRMS (ESI$^+$):** *m/z* [*M*+Na]$^+$ calcd. for $C_{26}H_{28}ONaSi$: 407.1807; found: 407.1815.

2-methyl-3-(triphenylsilyl)pent-4-en-1-yl 4-methylbenzenesulfonate (22a)

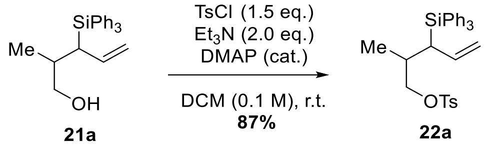

Following the general procedure 2.1, to the solution of alcohol **21a** (0.880 g, 2.45 mmol, 1.0 equiv.) in 25 mL of dry DCM (0.1 M) were added Et_3N (0.68 mL, 4.90 mmol, 2.0 equiv.), TsCl (0.701 g, 3.68 mmol, 1.5 equiv.) and a small amount of DMAP to give 1.09 g of tosylate **22a** as a white amorphous solid (2.13 mmol, 87% yield).

R_f = 0.67 (*n*-hexane/EtOAc 90:10); **^{1}H-NMR ($CDCl_3$, 500 MHz)** δ = 7.34 (d, *J* = 8.3 Hz, 2H), 7.54 (m, 6H), 7.41 (m, 3H), 7.34-7.28 (m, 8H), 5.72 (dt, *J* = 17.2 & 10.6 Hz, 1H), 4.97 (dd, *J* = 10.1 & 1.7 Hz, 1H), 4.86 (dd, *J* = 16.8 & 1.7 Hz, 1H), 3.86 (m, 1H), 3.74 97 (dd, *J* = 9.2 & 6.2 Hz, 1H), 2.75 97 (dd, *J* = 11.1 & 2.4 Hz, 1H), 2.44 (s, 3H), 2.40 (m, 1H), 0.66 (d, *J* = 7.0 Hz, 3H); **^{13}C{^{1}H}-NMR ($CDCl_3$, 125 MHz)** δ = 144.6 (C), 136.2 (6 x CH), 133.8 (3 x C), 133.3 (CH), 133.1 (C), 129.8 (2 x CH), 129.6 (3 x CH), 127.9 (2 x CH), 127.8 (6 x CH), 118.5 (CH_2), 74.1 (CH_2), 33.2 (CH), 32.4 (CH), 21.7 (CH_3), 13.7 (CH_3); **HRMS (ESI$^+$):** *m/z* [*M*+Na]$^+$ calcd. for $C_{31}H_{32}O_3NaSSi$:535.1739; found: 535.1745.

2-allyl-3-(triphenylsilyl)pent-4-en-1-yl 4-methylbenzenesulfonate (22b)

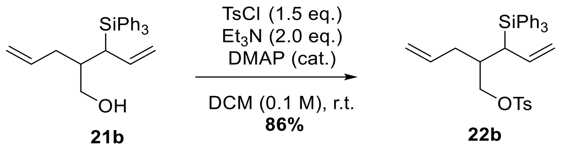

Following the general procedure 2.1, to the solution of alcohol **21b** (0.625 g, 1.63 mmol, 1.0 equiv.) in 16 mL of dry DCM (0.1 M) were added Et_3N (0.57 mL, 4.08 mmol, 2.5 equiv.), TsCl (0.465 g, 2.44 mmol, 1.5 equiv.) and a small amount of DMAP to afford 0.754 g of tosylate **22b** as a white amorphous solid (1.40 mmol, 86% yield).

R_f = 0.50 (*n*-hexane/EtOAc 80:20); **^{1}H-NMR ($CDCl_3$, 400 MHz)** δ = 7.74 (d, *J* = 8.2 Hz, 2H), 7.56 (m, 6H), 7.44-7.29 (m, 11H), 5.67 (dt, *J* = 17.0 & 10.6 Hz, 1H), 5.38 (m, 1H), 4.99 (dd, *J* = 10.0 & 1.6 Hz, 1H), 4.84 (m, 2H), 4.70 (dd, *J* = 17.0 & 0.8 Hz, 1H), 3.93 (dd, *J* = 9.4 & 4.2 Hz, 1H), 3.85 (t, *J* = 9.2 Hz, 1H), 2.88 (dd, *J* = 11.2 & 2.6 Hz, 1H), 2.45 (s, 3H), 2.29 (m, 1H), 1.95 (m, 1H), 1.60 (m, 1H); **^{13}C{^{1}H}-NMR ($CDCl_3$, 100 MHz)** δ = 144.5 (C), 136.2 (6 x CH), 135.4 (CH), 133.7 (CH), 133.6 (3 x C), 133.1 (C), 129.7 (2 x CH), 129.6 (3 x CH), 127.9 (2 x CH), 127.8 (6 x CH), 118.8 (CH_2), 116.9 (CH_2), 71.1 (CH_2), 37.5 (CH), 32.4 (CH_2), 32.3 (CH), 21.6 (CH_3); **HRMS (ESI$^+$):** *m/z* [*M*+Na]$^+$ calcd. for $C_{33}H_{34}O_3NaSSi$: 561.1896; found: 561.1900.

4-methyl-*N*-(2-methyl-3-(triphenylsilyl)pent-4-en-1-yl)benzenesulfonamide (9a)

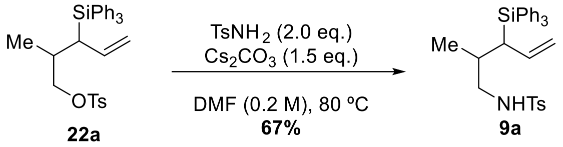

Following the general procedure 2.2, to a solution of tosylate **22a** (1.09 g, 2.13 mmol, 1.0 equiv.) in 11 mL of dry DMF (0.2 M) were added $TsNH_2$ (0.729 g, 4.26 mmol, 2.0 equiv.) and Cs_2CO_3 (1.04 g, 3.20 mmol, 1.5 equiv.) to obtain 0.732 g of tosylamine **9a** as a white amorphous solid (1.43 mmol, 67% yield).

R_f = 0.55 (*n*-hexane/EtOAc 90:10); **^{1}H-NMR ($CDCl_3$, 400 MHz)** δ = 7.65 (d, *J* = 8.1 Hz, 2H), 7.52 (m, 6H), 7.43-7.38 (m, 3H), 7.37-7.31 (m, 6H), 7.23 (d, *J* = 8.0 Hz, 2H), 5.80 (dt, *J* = 17.1 & 10.6 Hz, 1H), 5.00 (dd, *J* = 10.0 & 1.7 Hz, 1H), 4.92 (dd, *J* = 16.9 & 1.4 Hz, 1H), 4.27 (brt, *J* = 6.5 Hz, 1H), 2.85 (m, 1H), 2.7-2.72 (dd, *J* = 12.5 & 6.3 Hz, 1H), 2.72-2.68 (dd, *J* = 11.1 & 2.7 Hz, 1H), 2.40 (s, 3H), 2.13 (m, 1H), 0.72 (d, *J* = 6.9 Hz, 3H); **^{13}C{^{1}H}-NMR ($CDCl_3$, 100 MHz)** δ = 143.2 (C), 137.1 (C), 136.2 (6 x CH), 134.2 (CH), 133.9 (3 x C), 129.6 (2 x CH), 129.5 (3 x CH), 127.8 (6 x CH), 127.0 (2 x CH), 117.9 (CH_2), 49.0 (CH_2), 34.8 (CH), 32.9 (CH), 21.5 (CH_3), 15.4 (CH_3); **HRMS (ESI$^+$):** *m/z* [*M*-H]$^+$ calcd. for $C_{31}H_{32}NO_2SSi$: 510.1923; found: 510.1925.

***N*-(2-allyl-3-(triphenylsilyl)pent-4-en-1-yl)-4-methylbenzenesulfonamide (9b)**

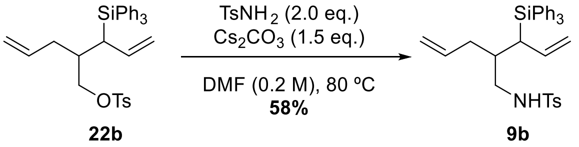

Following the general procedure 2.2, to a solution of tosylate **22b** (0.400 g, 0.74 mmol, 1.0 equiv.) in 3.7 mL of dry DMF (0.2 M) were added $TsNH_2$ (0.254 g, 1.48 mmol, 2.0 equiv.) and Cs_2CO_3 (0.362 g, 1.11 mmol, 1.5 equiv.) to give 0.231 g of tosylamine **9b** as a white amorphous solid (0.43 mmol, 58% yield).

R_f = 0.45 (*n*-hexane/EtOAc 80:20); **^{1}H-NMR ($CDCl_3$, 500 MHz)** δ = 7.63 (d, *J* = 8.2 Hz, 2H), 7.54 (m, 5H), 7.41 (m, 3H), 7.35 (m, 7H), 7.23 (d, *J* = 8.0 Hz, 2H), 5.77 (dt, *J* = 16.9 & 10.5 Hz, 1H), 5.45 (m, 1H), 5.02 (dd, *J* = 9.9 & 1.4 Hz, 1H), 4.95 (d, *J* = 16.9 Hz, 1H), 4.87 (d, *J* = 10.1 Hz, 1H), 4.75 (d, *J* = 17.1 Hz, 1H), 4.35 (dd, *J* = 7.1 & 6.0 Hz, 1H), 2.86 (m, 3H), 2.40 (s, 3H), 2.03 (m, 2H), 1.73 (m, 1H); **^{13}C{^{1}H}-NMR ($CDCl_3$, 100 MHz)** δ = 143.2 (C), 136.9 (CH), 136.4 (5 x CH), 136.2 (C), 134.9 (CH), 133.7 (3 x C),

129.6 (5 x CH), 127.9 (7 x CH), 127.0 (2 x CH), 118.1 (CH_2), 116.8 (CH_2), 45.8 (CH_2), 38.0 (CH), 34.3 (CH), 34.2 (CH_2), 21.5 (CH_3); **HRMS (ESI$^+$):** *m/z* [*M*+Na]$^+$ calcd. for $C_{33}H_{35}NO_2NaSSi$: 560.2055; found: 560.2056.

3. NMR spectra

^{1}H-NMR ($CDCl_3$, 400 MHz)

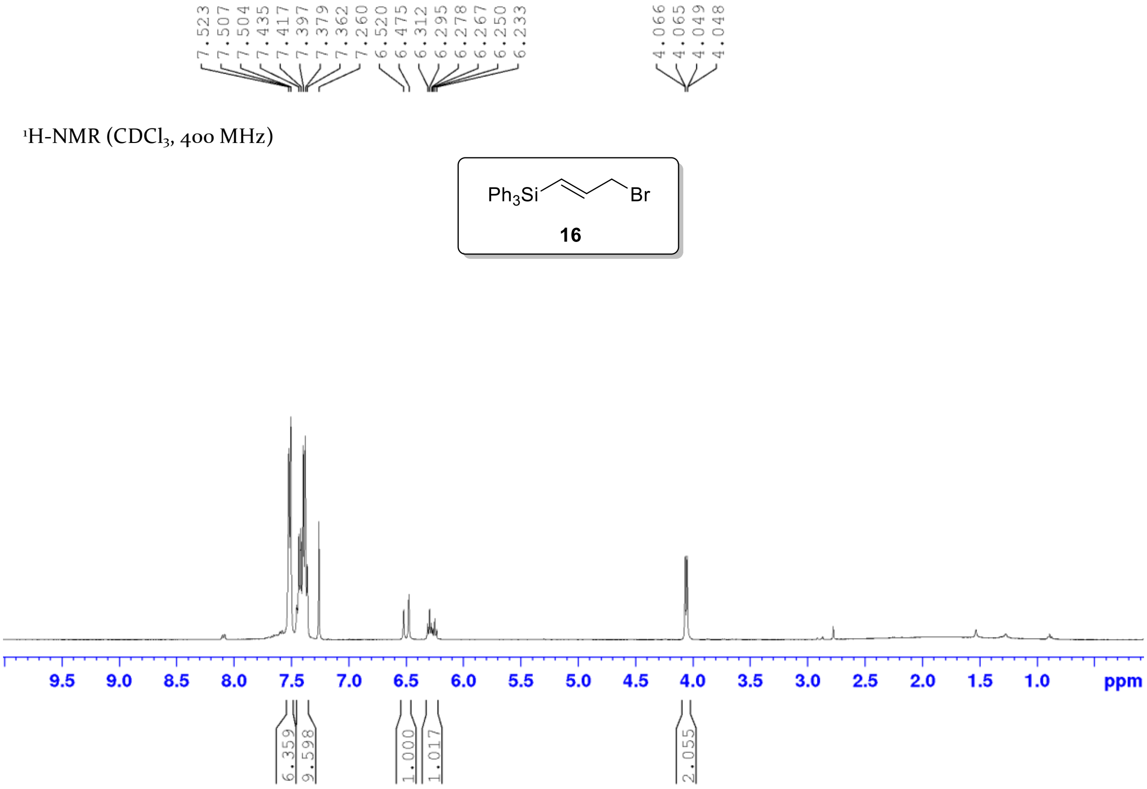

^{13}C{^{1}H}-NMR ($CDCl_3$, 125 MHz)

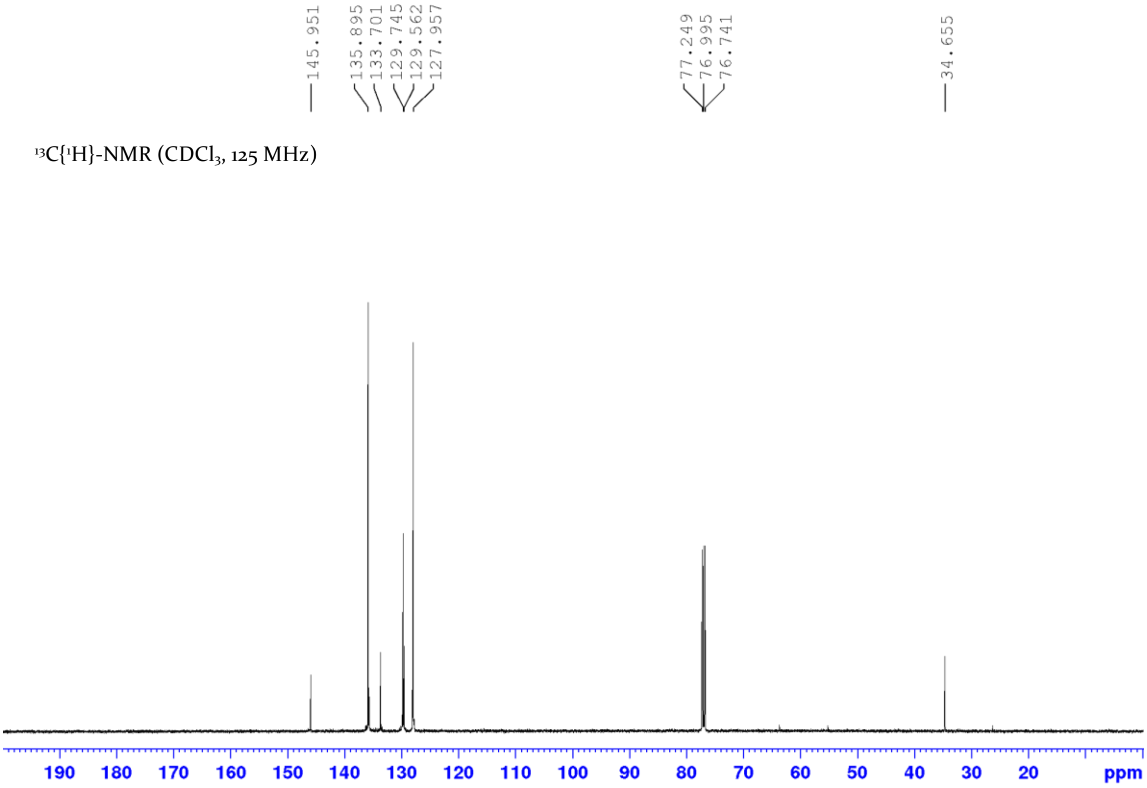

¹H-NMR (CDCl₃, 500 MHz)

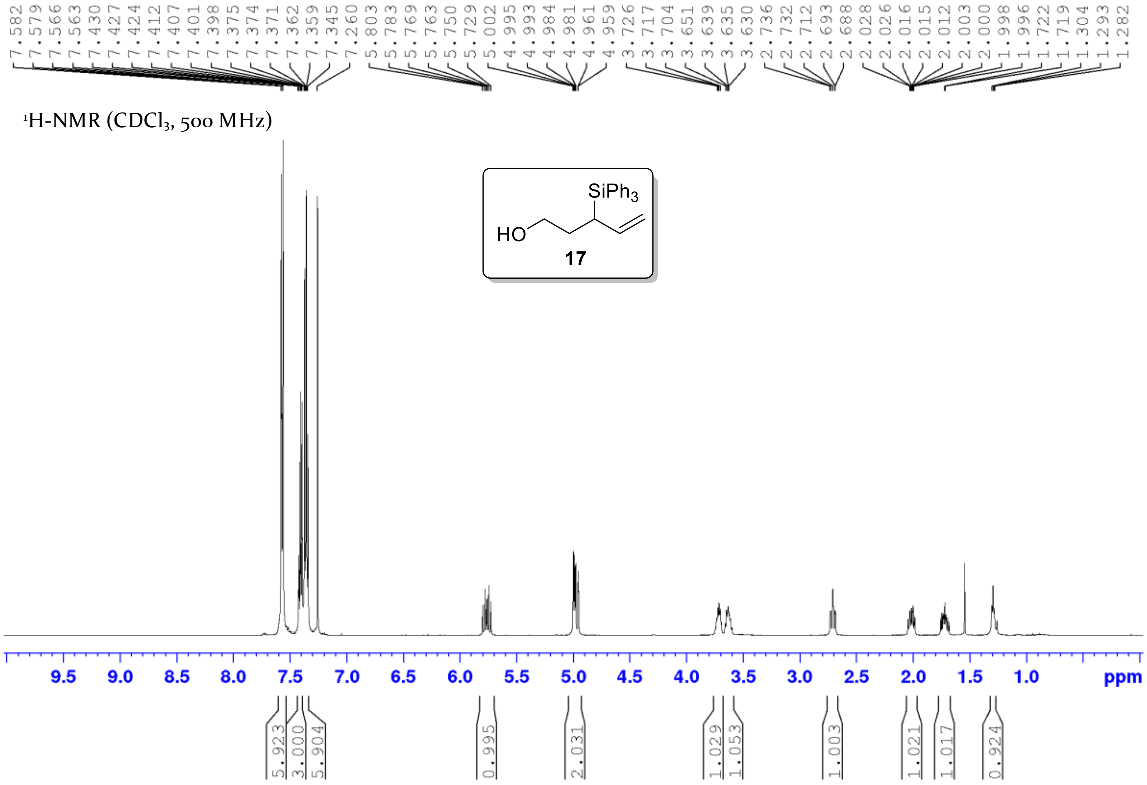

¹³C{¹H}-NMR (CDCl₃, 125 MHz)

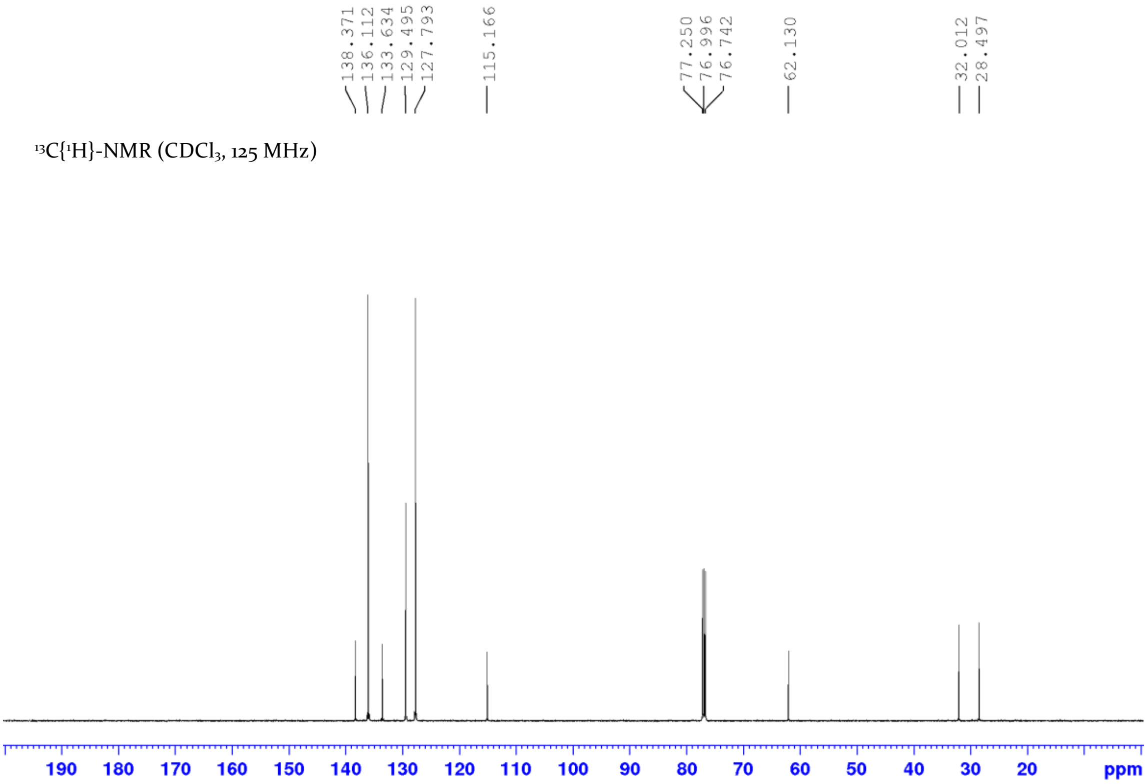

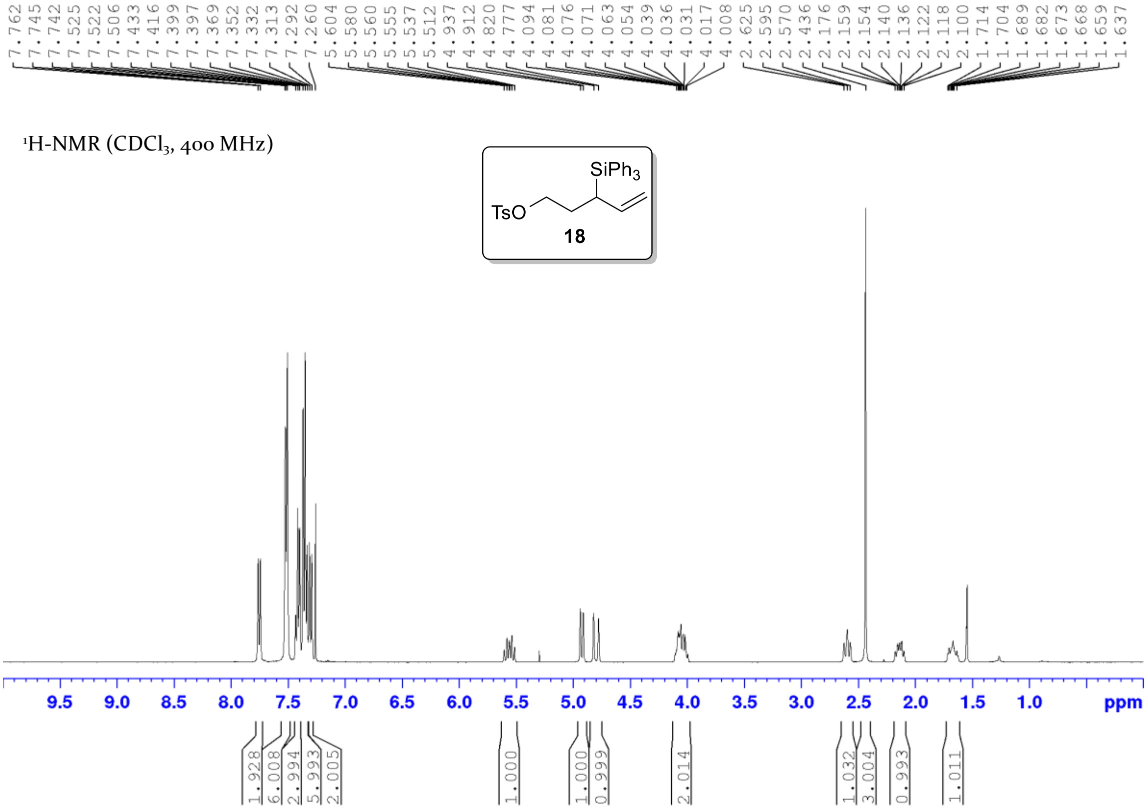

^{1}H-NMR ($CDCl_3$, 400 MHz)

18

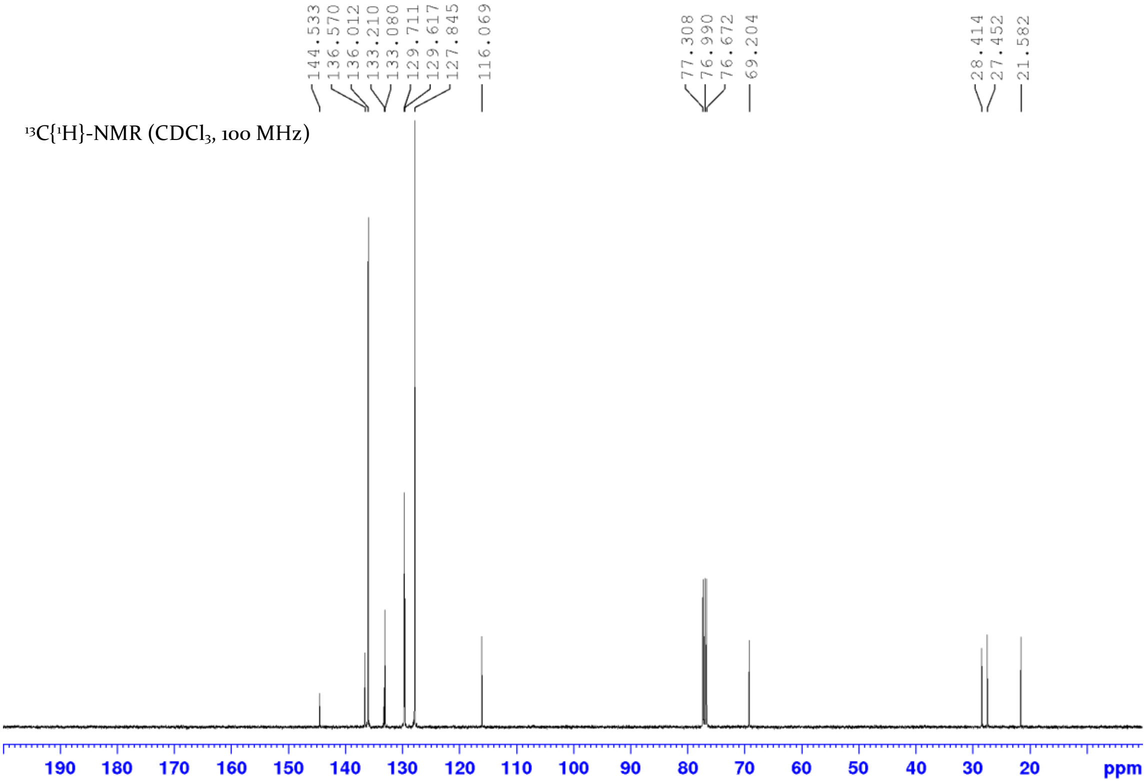

^{13}C{^{1}H}-NMR ($CDCl_3$, 100 MHz)

¹H-NMR (CDCl$_3$, 400 MHz)

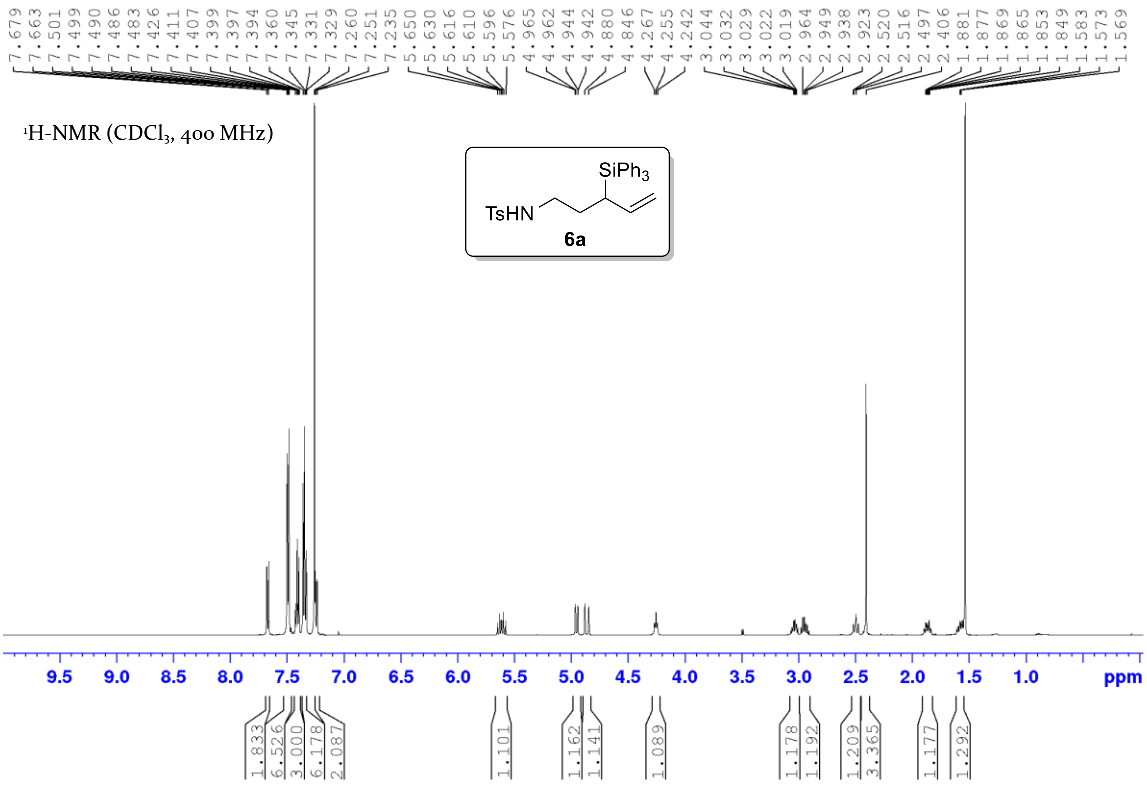

¹³C{¹H}-NMR (CDCl$_3$, 100 MHz)

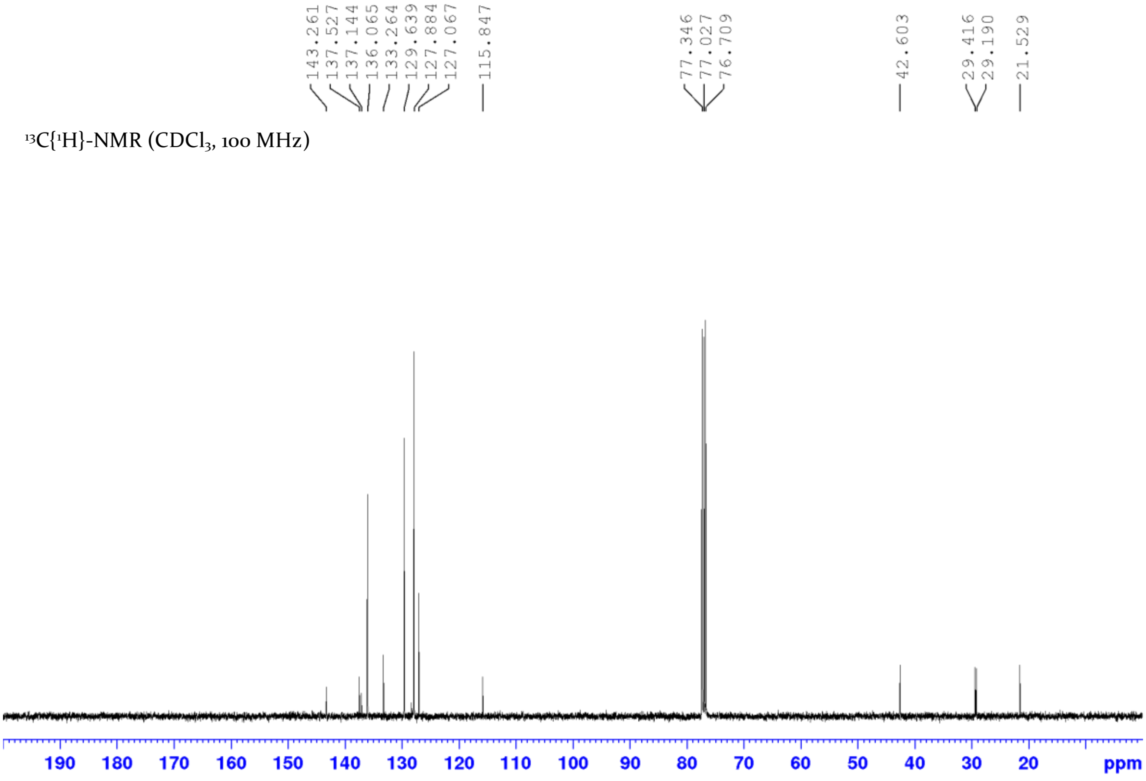

¹H-NMR (CDCl$_3$, 400 MHz)

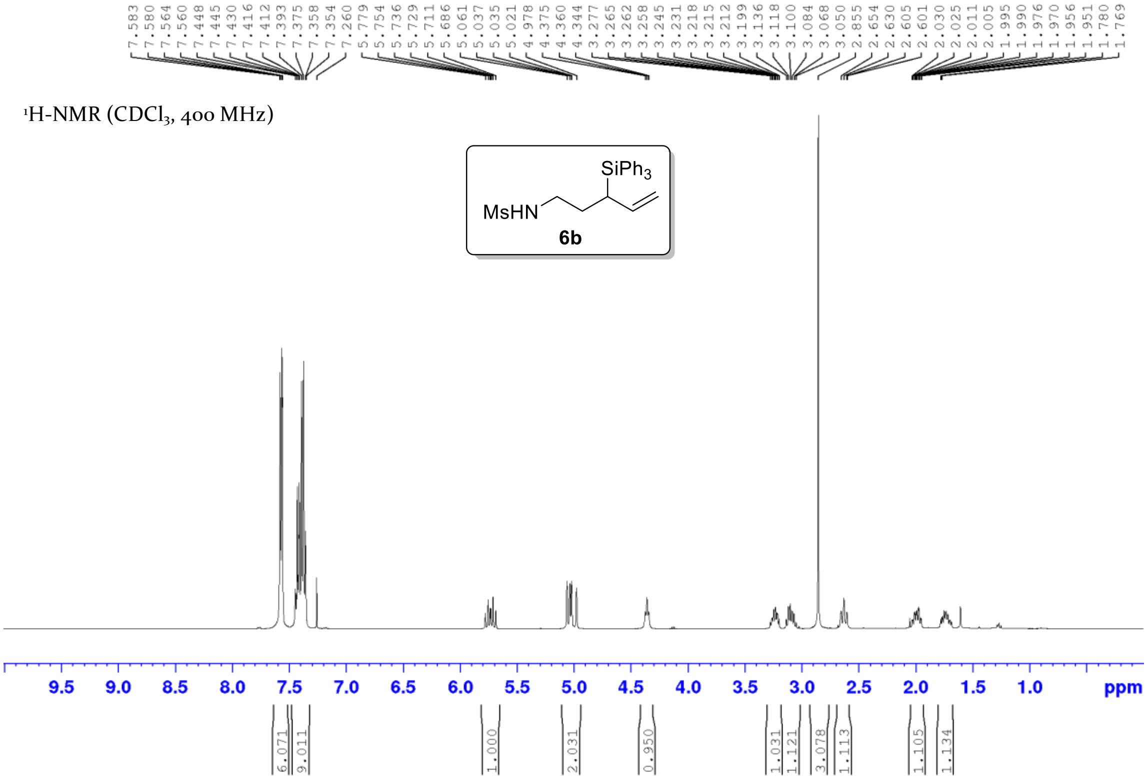

¹³C{¹H}-NMR (CDCl$_3$, 125 MHz)

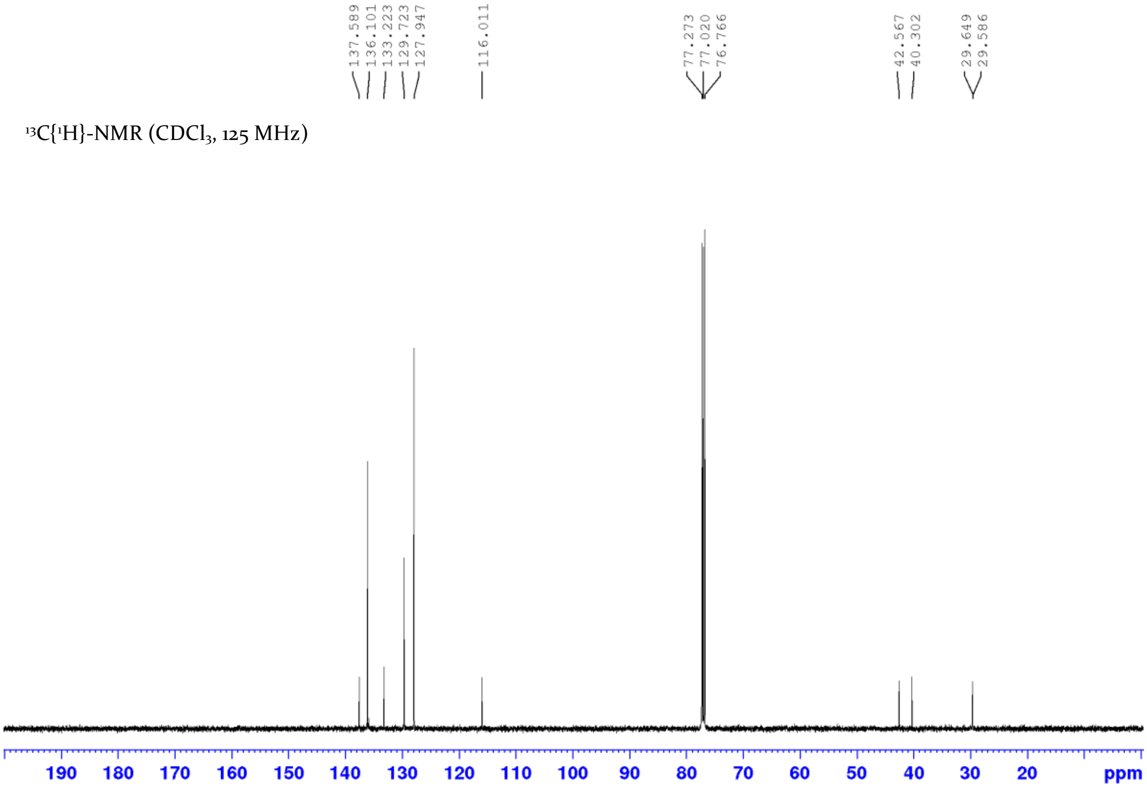

^{1}H-NMR ($CDCl_3$, 400 MHz)

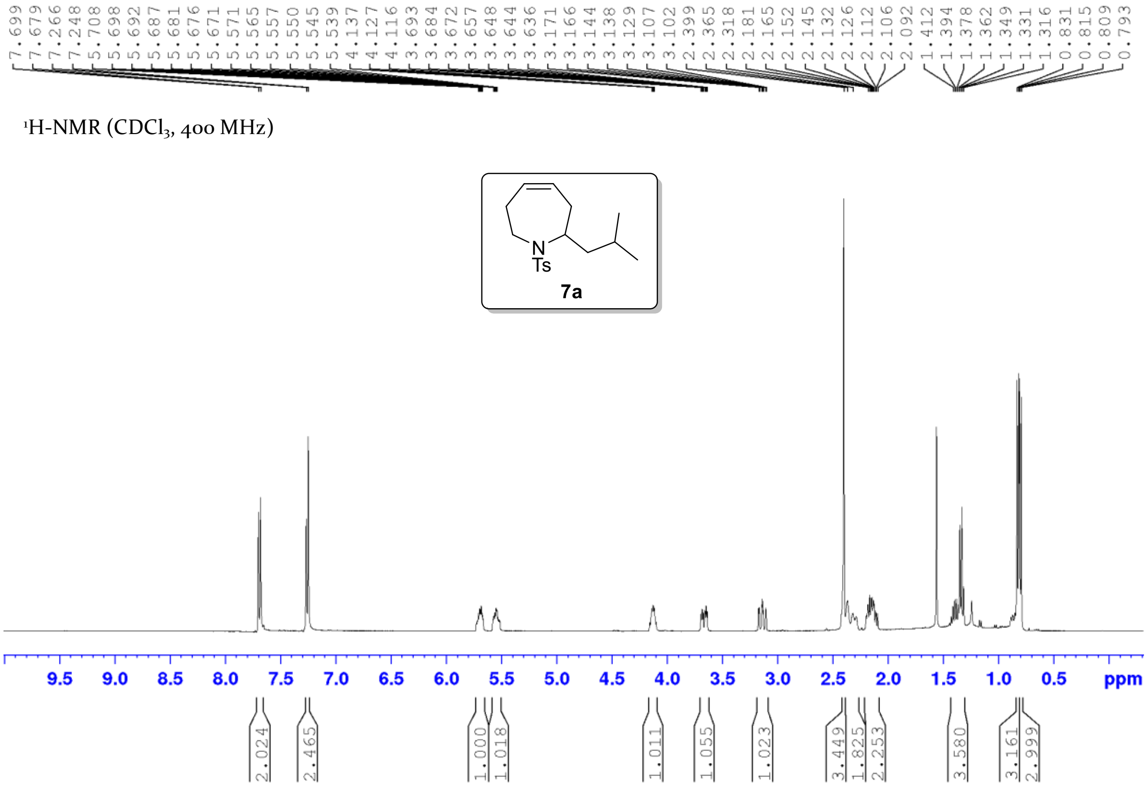

^{13}C{^{1}H}-NMR ($CDCl_3$, 125 MHz)

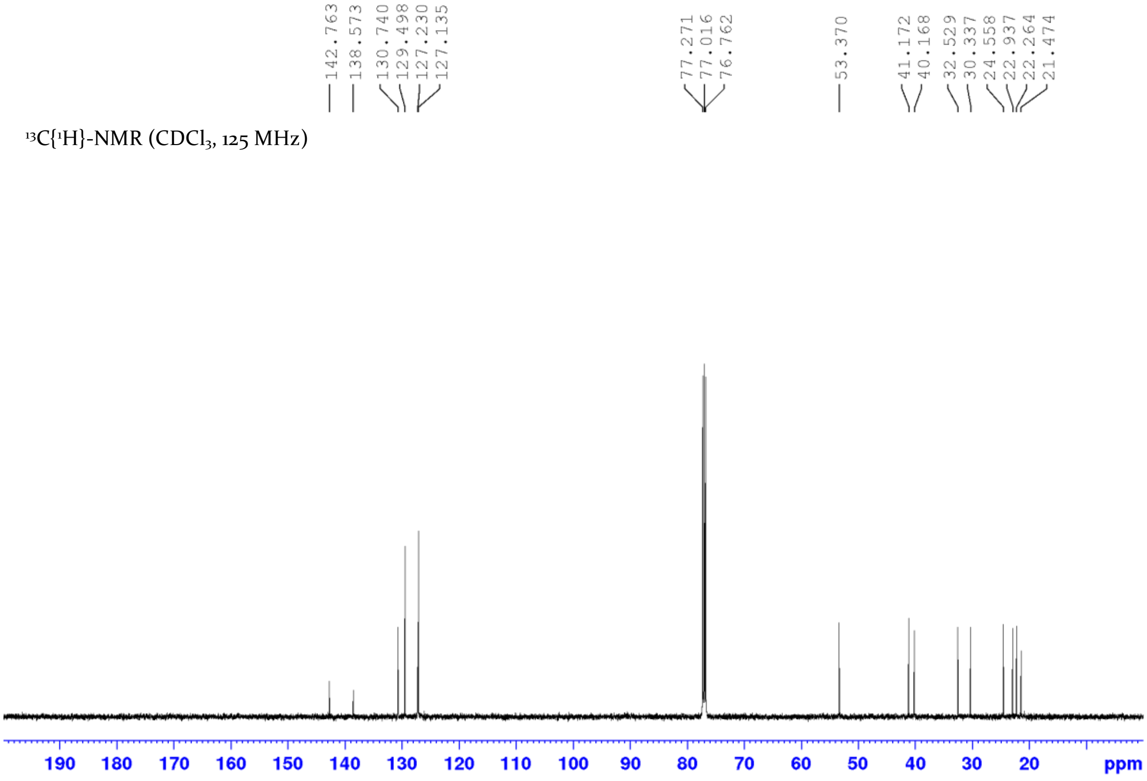

^{1}H-NMR ($CDCl_3$, 400 MHz)

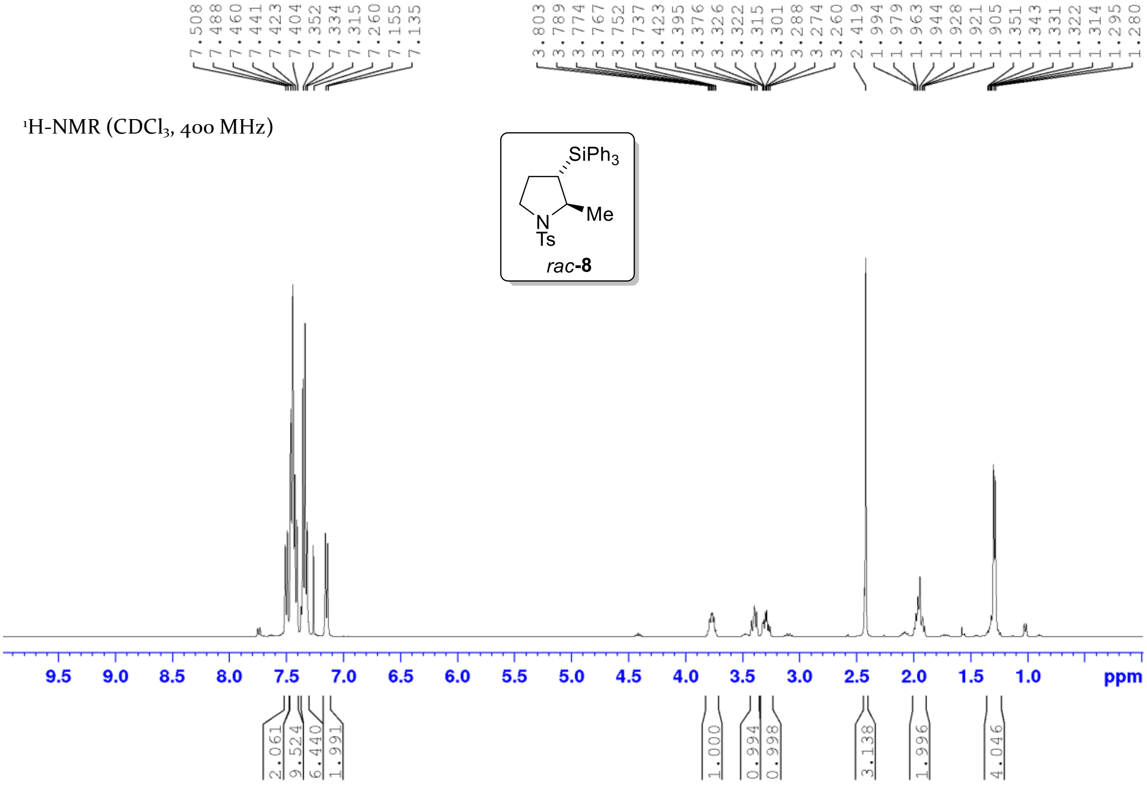

^{13}C{^{1}H}-NMR ($CDCl_3$, 100 MHz)

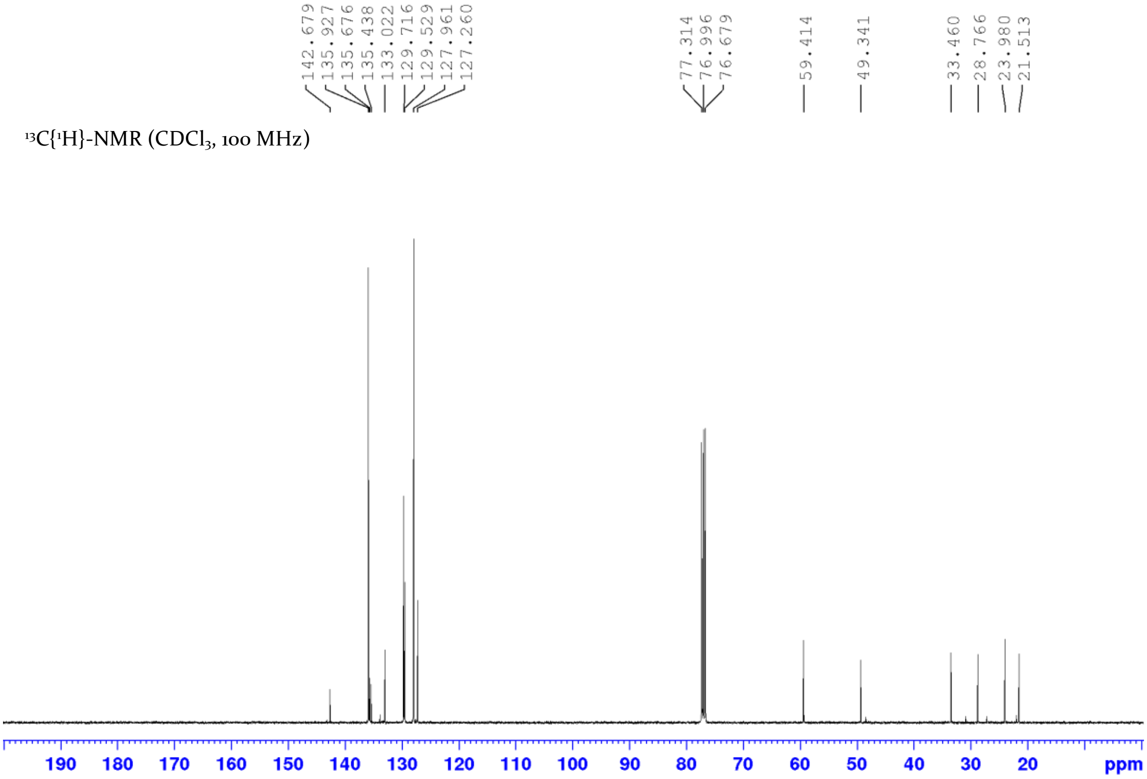

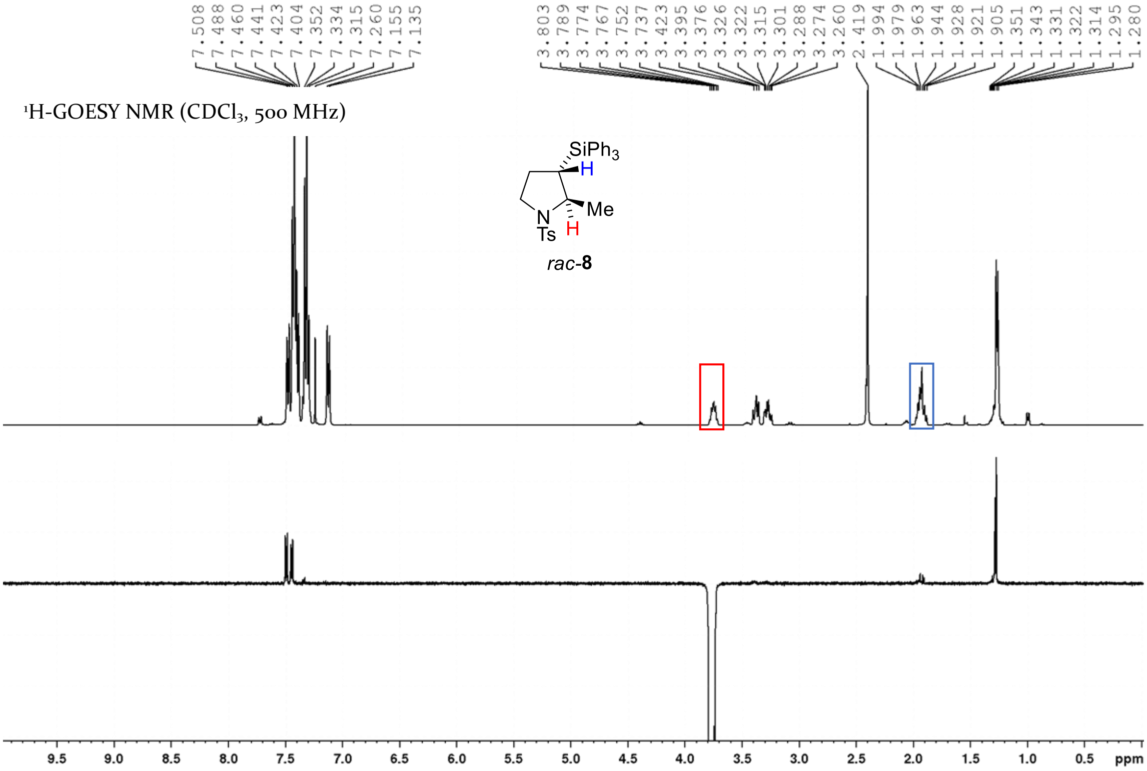

7.508 7.488 7.460 7.441 7.423 7.404 7.352 7.334 7.315 7.260 7.155 7.135
3.803 3.789 3.774 3.767 3.752 3.737 3.423 3.395 3.376 3.326 3.322 3.315 3.301 3.288 3.274 3.260 2.419 1.994 1.979 1.963 1.944 1.928 1.921 1.905 1.351 1.343 1.331 1.322 1.314 1.295 1.280
¹H-GOESY NMR (CDCl3, 500 MHz)
SiPh3
H
Me
N
Ts
H
rac-8
9.5 9.0 8.5 8.0 7.5 7.0 6.5 6.0 5.5 5.0 4.5 4.0 3.5 3.0 2.5 2.0 1.5 1.0 0.5 ppm

^{1}H-NMR ($CDCl_3$, 400 MHz)

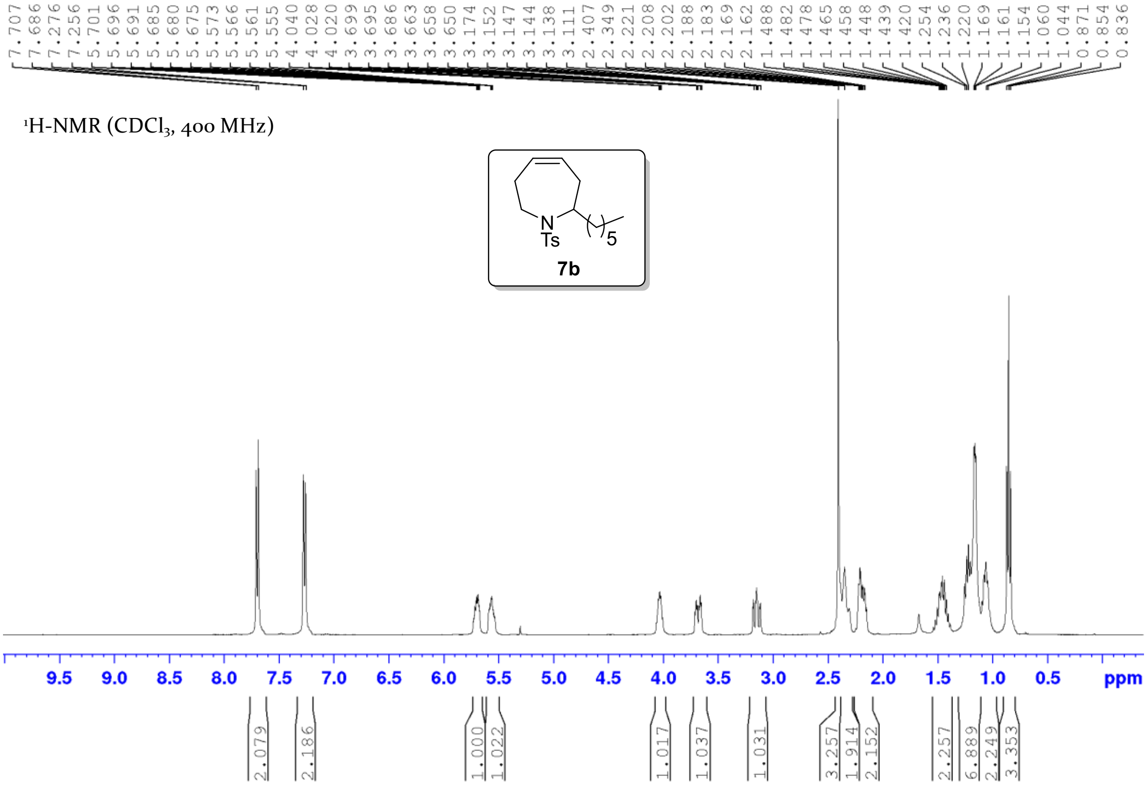

^{13}C{^{1}H}-NMR ($CDCl_3$, 100 MHz)

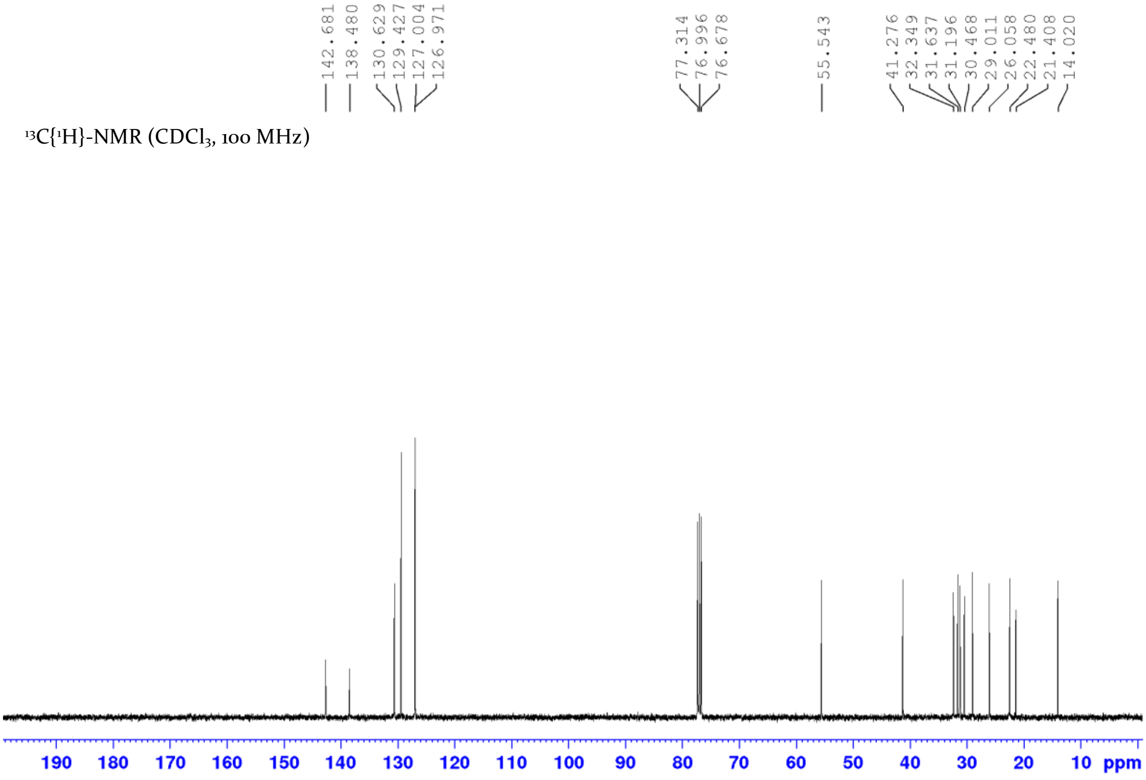

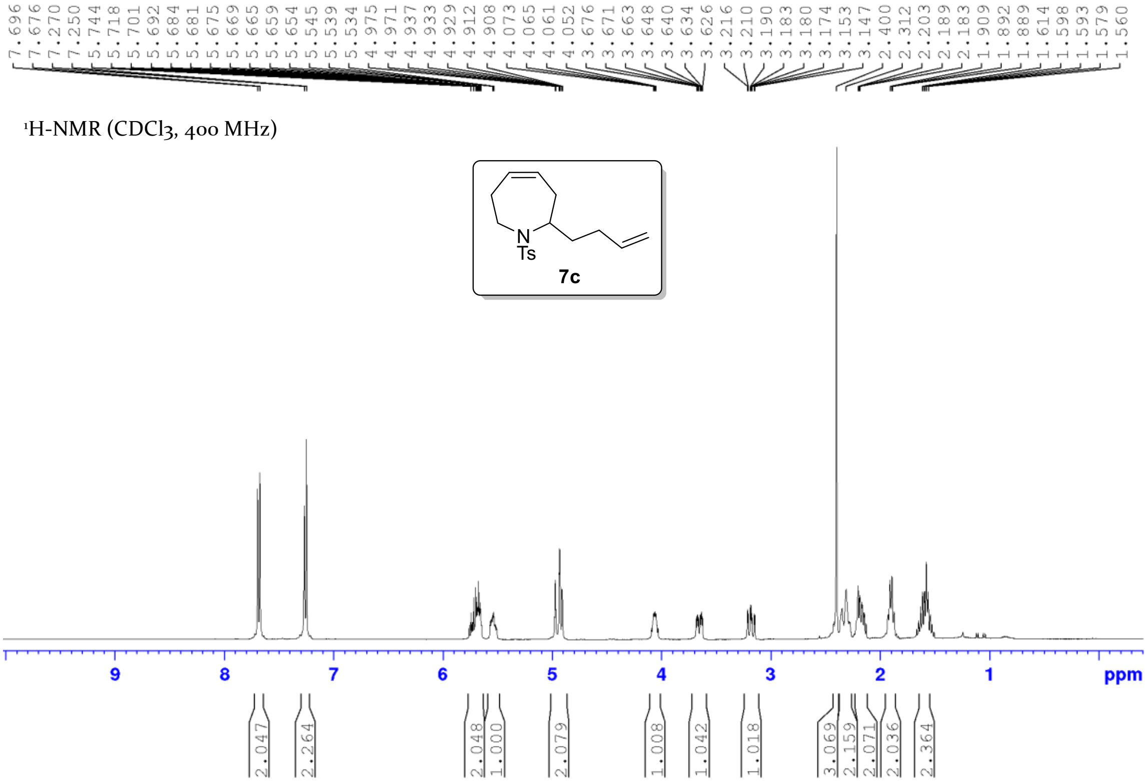

^{1}H-NMR ($CDCl_3$, 400 MHz)

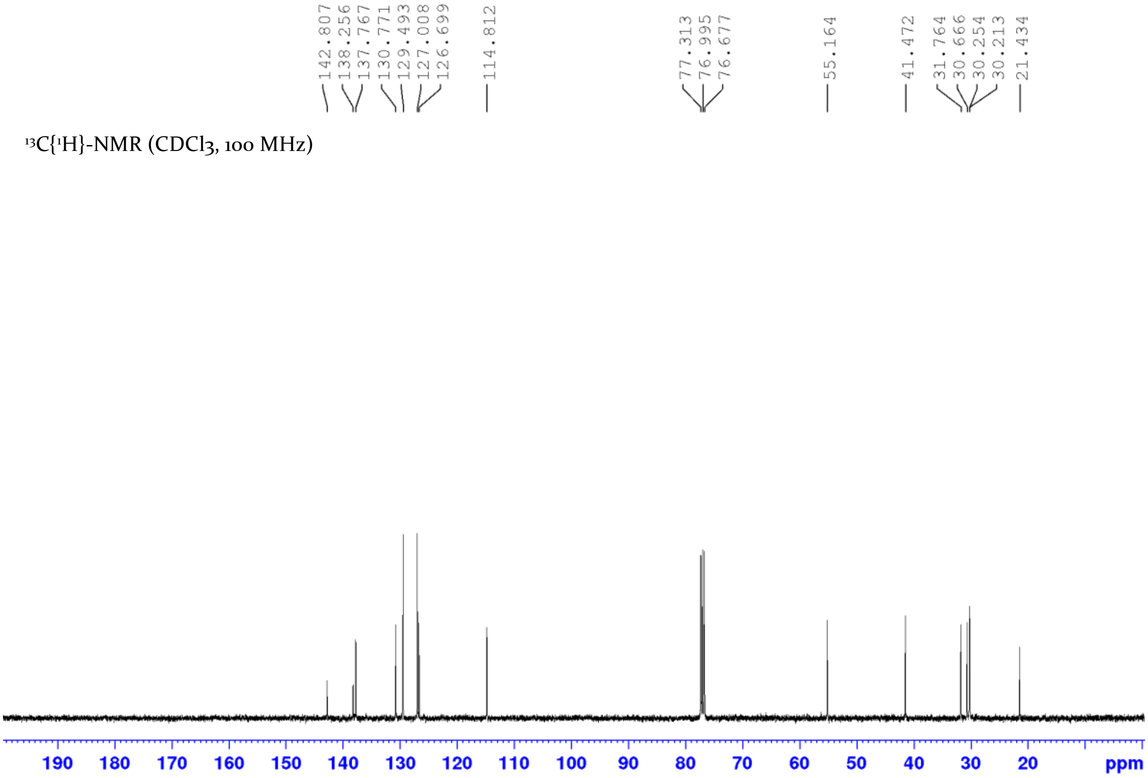

^{13}C{^{1}H}-NMR ($CDCl_3$, 100 MHz)

¹H-NMR (CDCl₃, 400 MHz)

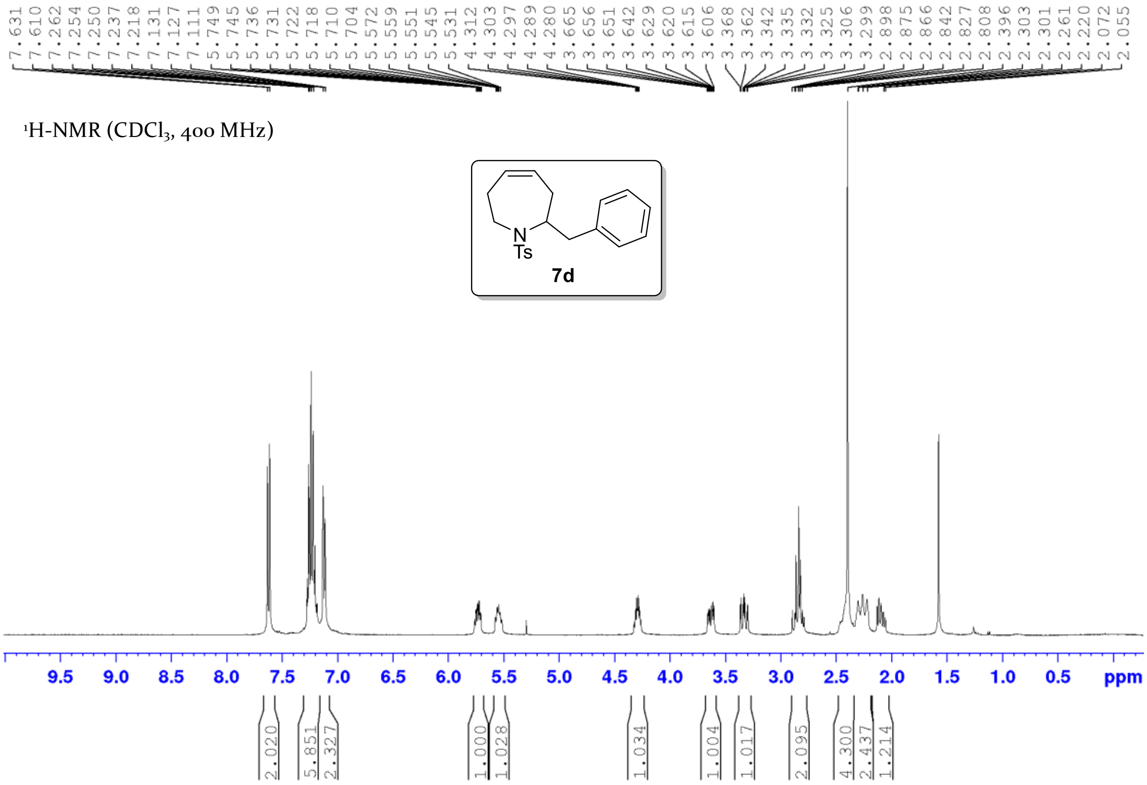

¹³C{¹H}-NMR (CDCl₃, 100 MHz)

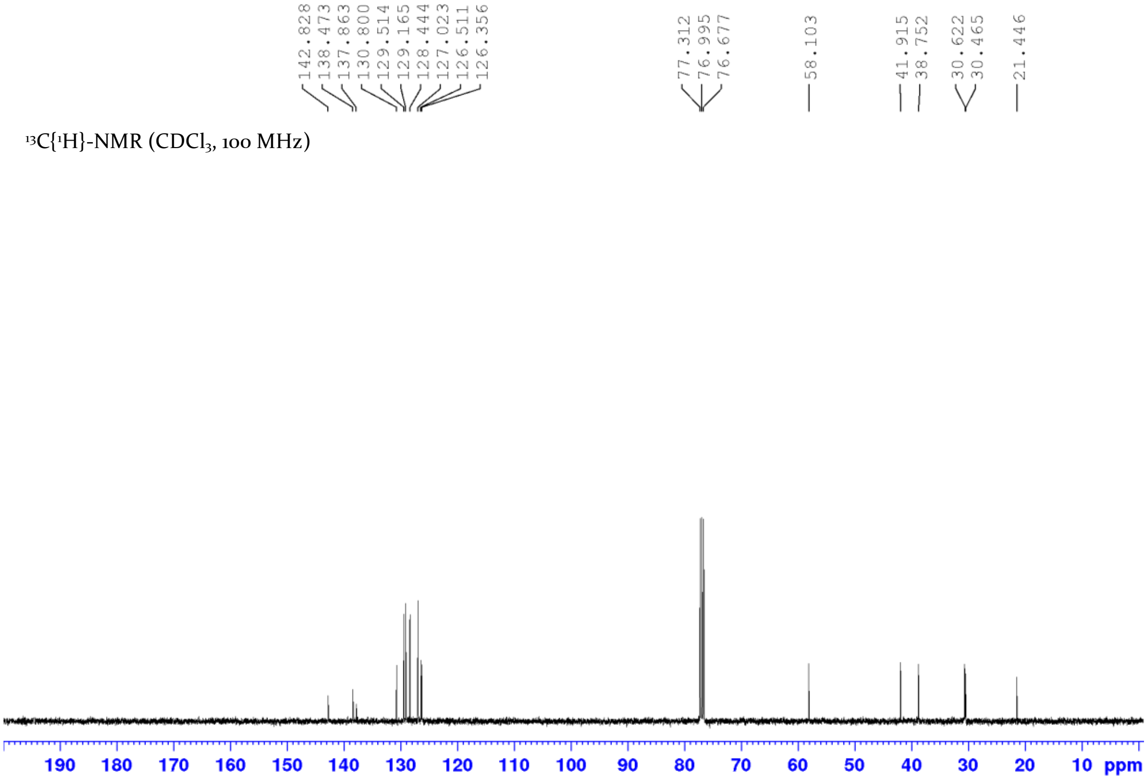

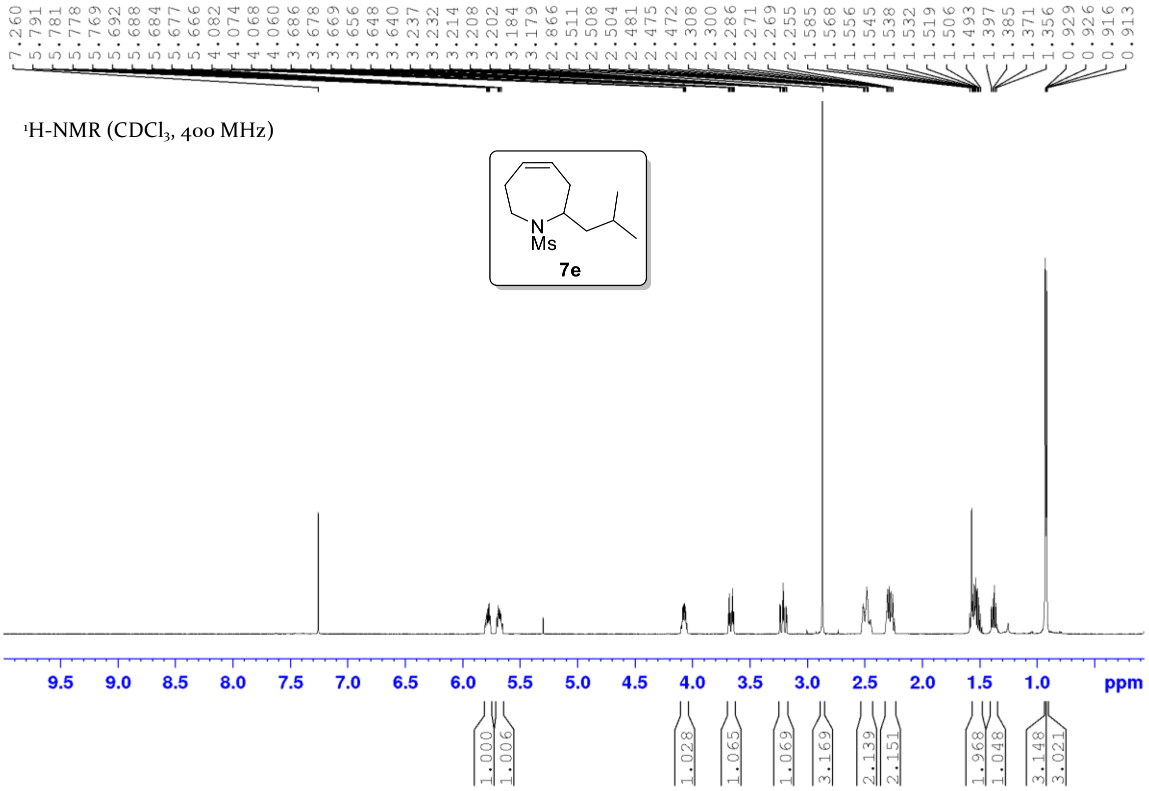

7.260
5.791
5.781
5.778
5.769
5.692
5.688
5.684
5.677
5.666
4.082
4.074
4.068
4.060
3.686
3.678
3.669
3.656
3.648
3.640
3.237
3.232
3.214
3.208
3.202
3.184
3.179
2.866
2.511
2.508
2.504
2.481
2.475
2.472
2.308
2.300
2.286
2.271
2.269
2.255
1.585
1.568
1.556
1.545
1.538
1.532
1.519
1.506
1.493
1.397
1.385
1.371
1.356
0.929
0.926
0.916
0.913
¹H-NMR (CDCl₃, 400 MHz)
N
Ms
7e
9.5
9.0
8.5
8.0
7.5
7.0
6.5
6.0
5.5
5.0
4.5
4.0
3.5
3.0
2.5
2.0
1.5
1.0
ppm
1.000
1.006
1.028
1.065
1.069
3.169
2.139
2.151
1.968
1.048
3.148
3.021

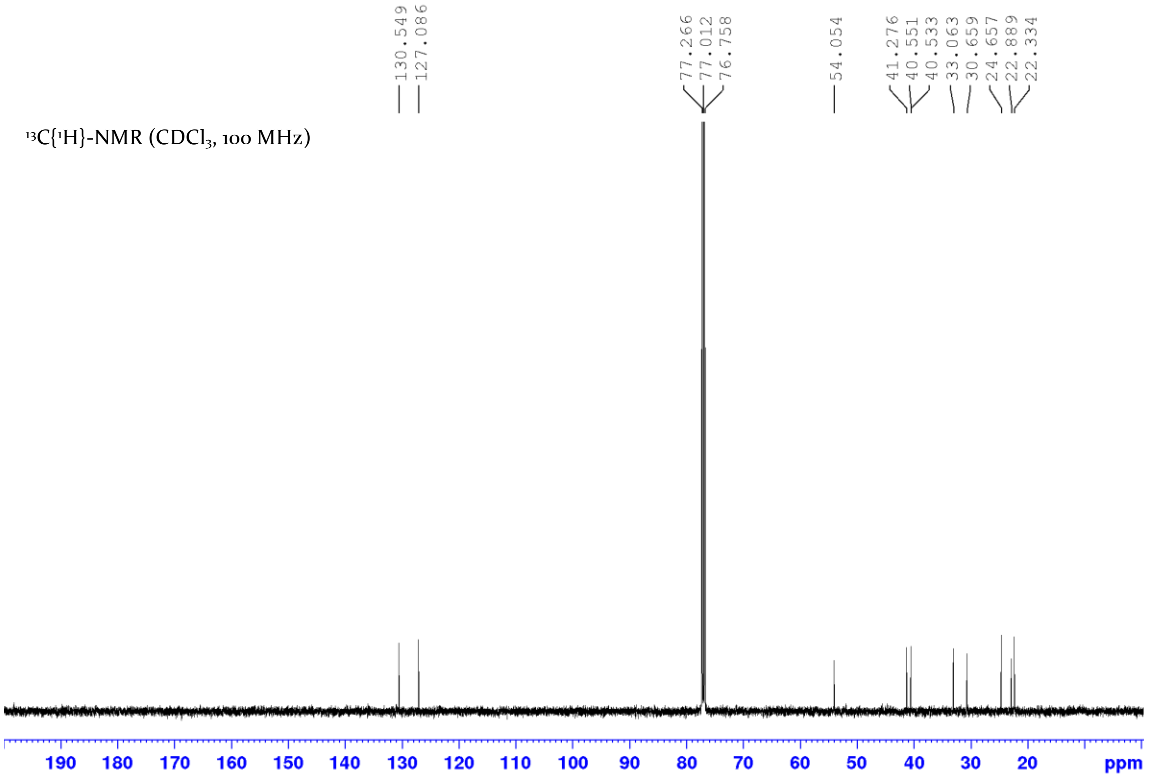

130.549
127.086
77.266
77.012
76.758
54.054
41.276
40.551
40.533
33.063
30.659
24.657
22.889
22.334
¹³C{¹H}-NMR (CDCl₃, 100 MHz)
190
180
170
160
150
140
130
120
110
100
90
80
70
60
50
40
30
20
ppm

^{1}H-NMR (CDCl$_3$, 400 MHz)

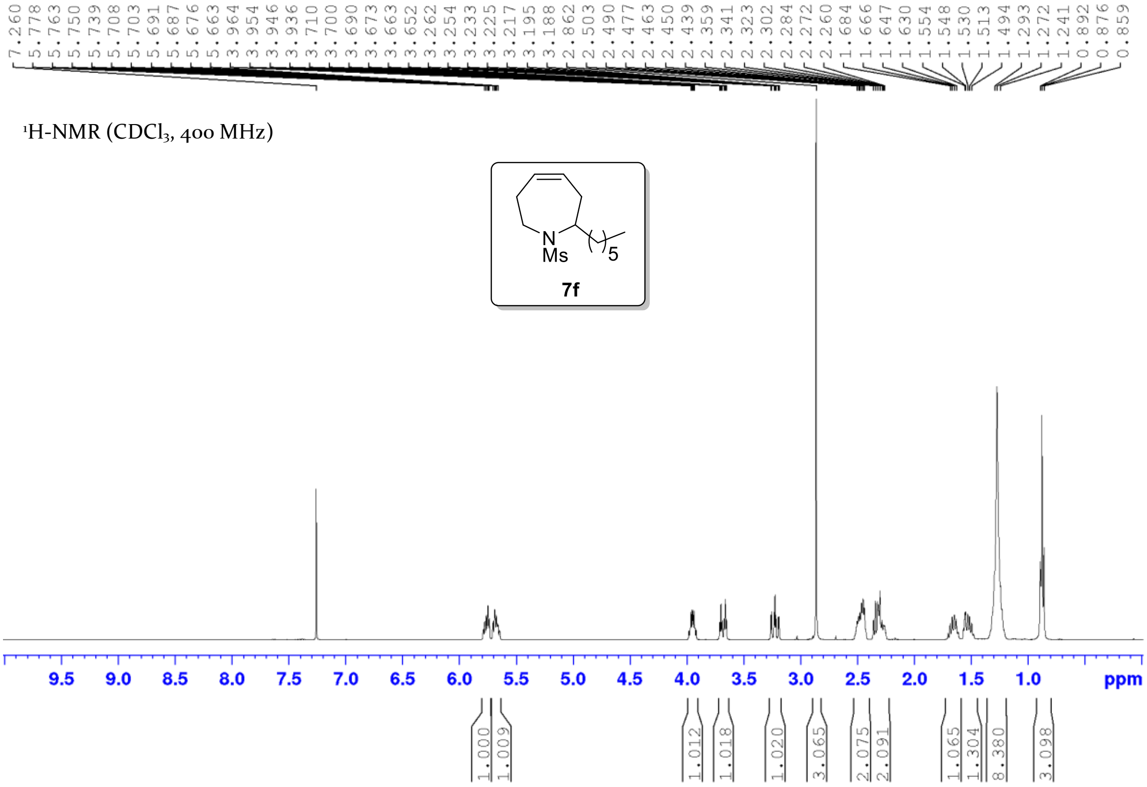

^{13}C{^{1}H}-NMR (CDCl$_3$, 125 MHz)

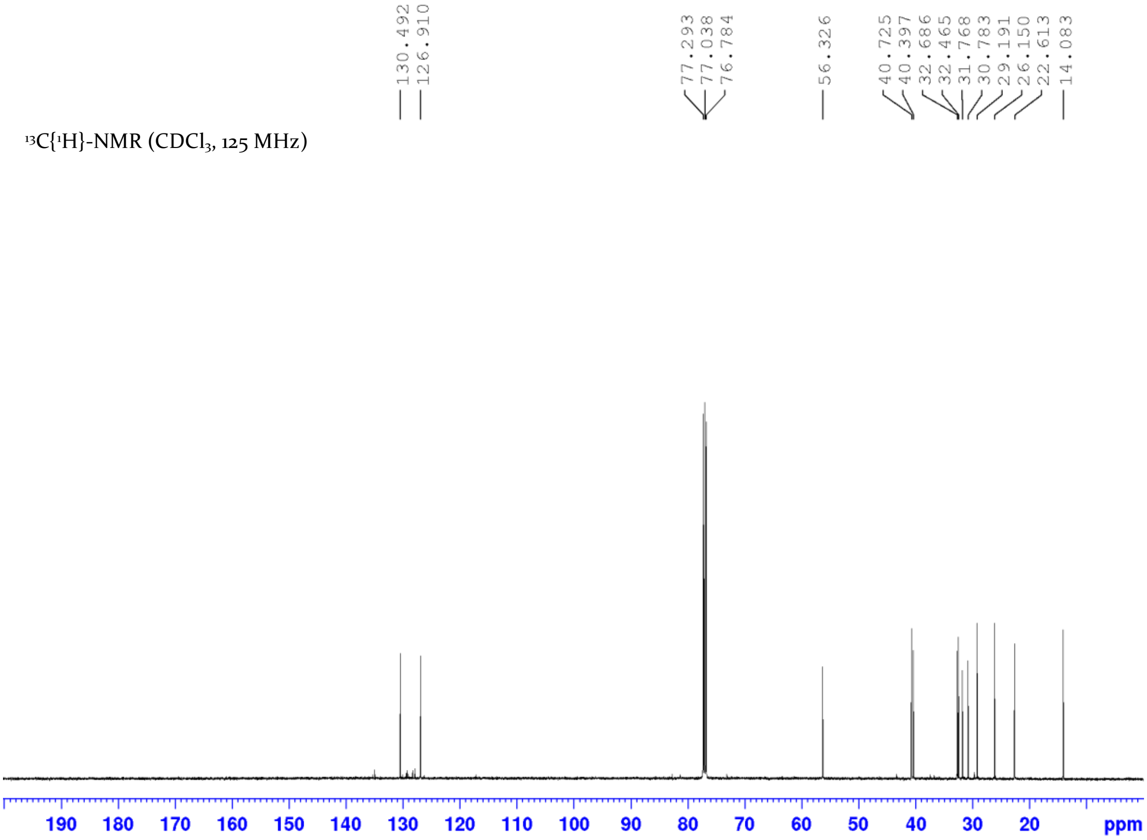

^{1}H-NMR ($CDCl_3$, 400 MHz)

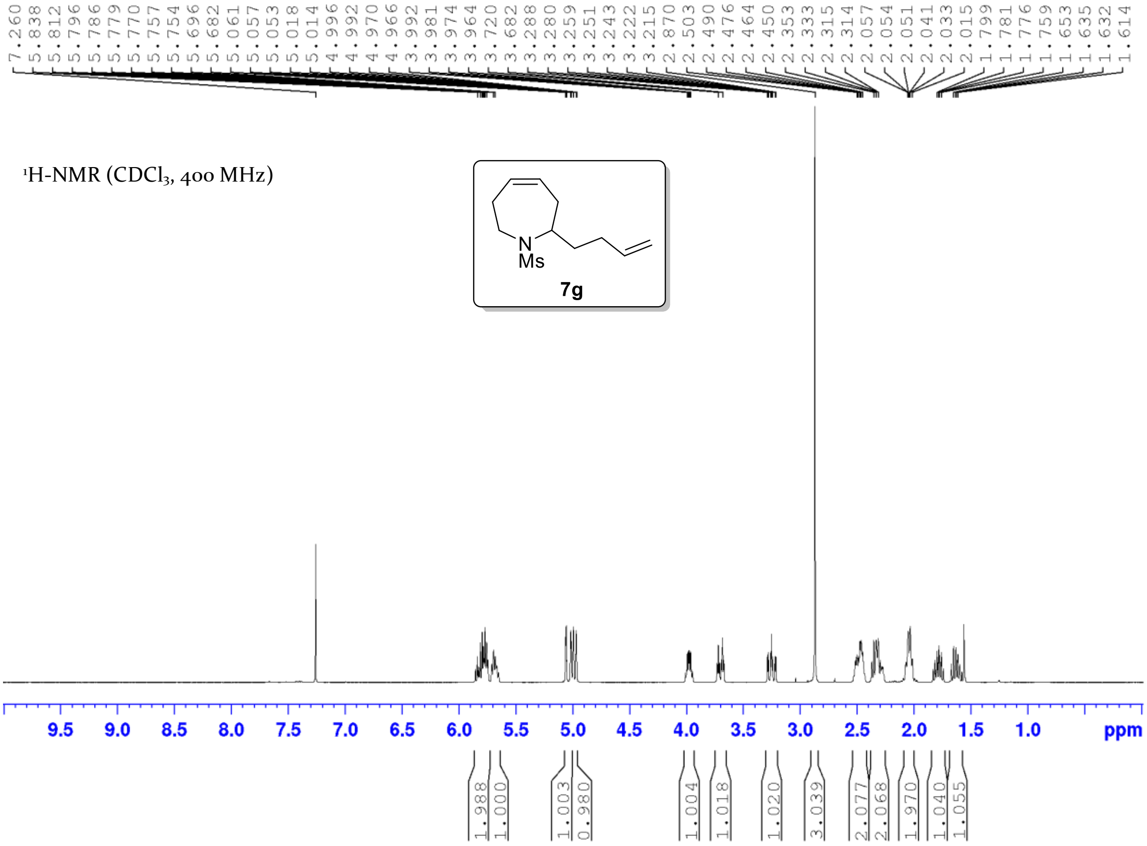

7g

^{13}C{^{1}H}-NMR ($CDCl_3$, 125 MHz)

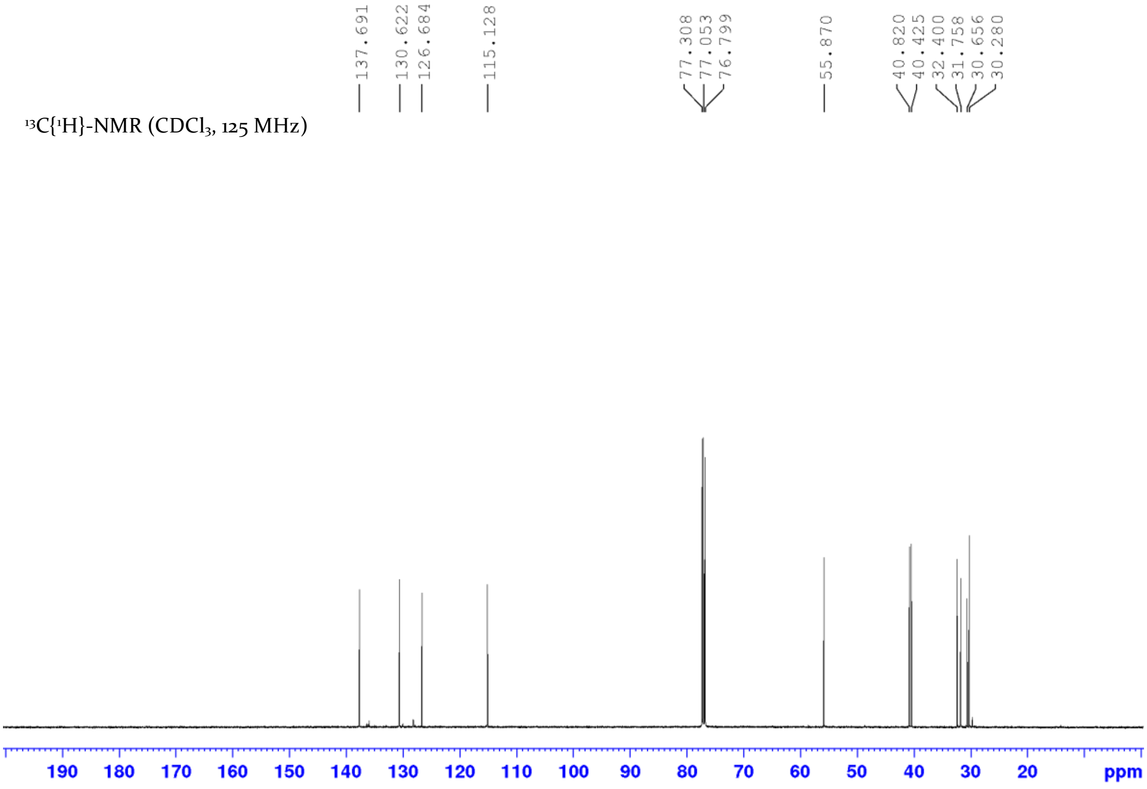

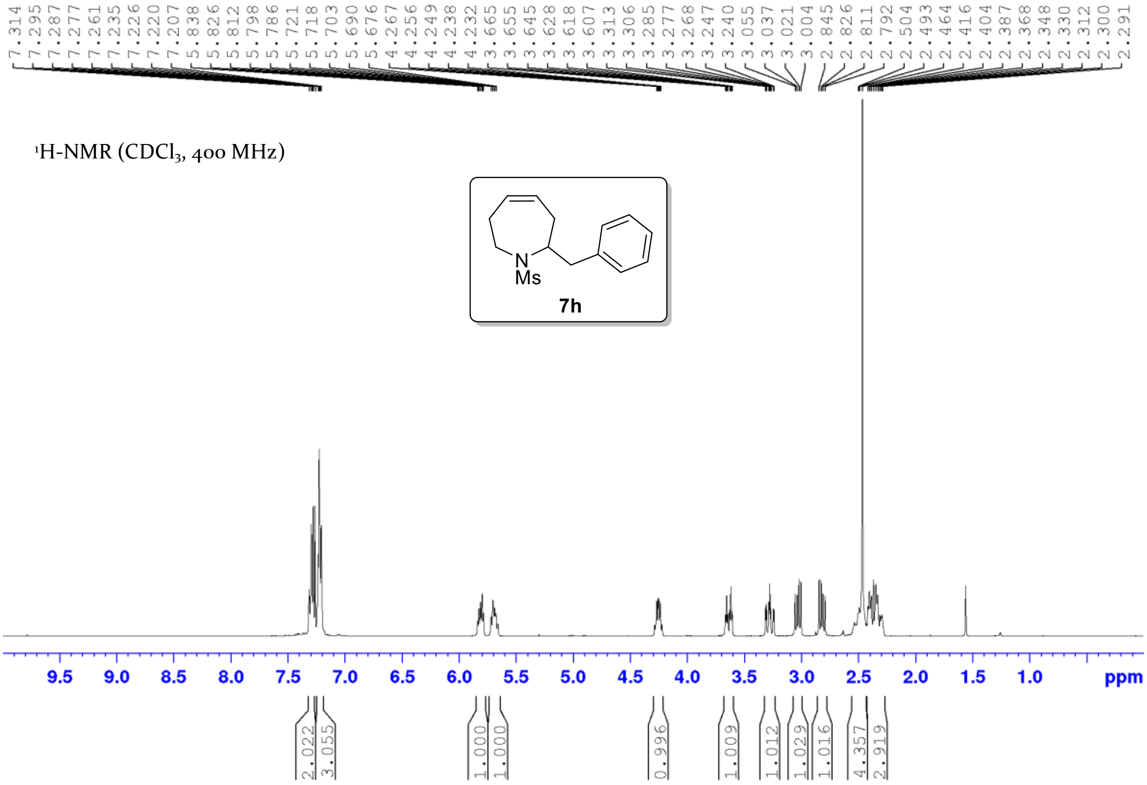
7.314 7.295 7.287 7.277 7.261 7.235 7.226 7.220 7.207 5.838 5.826 5.812 5.798 5.786 5.721 5.718 5.703 5.690 5.676 4.267 4.256 4.249 4.238 4.232 3.665 3.655 3.645 3.628 3.618 3.607 3.313 3.306 3.285 3.277 3.268 3.247 3.240 3.055 3.037 3.021 3.004 2.845 2.826 2.811 2.792 2.504 2.493 2.464 2.416 2.404 2.387 2.368 2.348 2.330 2.312 2.300 2.291
¹H-NMR (CDCl₃, 400 MHz)
N
Ms
7h
9.5 9.0 8.5 8.0 7.5 7.0 6.5 6.0 5.5 5.0 4.5 4.0 3.5 3.0 2.5 2.0 1.5 1.0 ppm
2.022 3.055 1.000 1.000 0.996 1.009 1.012 1.029 1.016 4.357 2.919

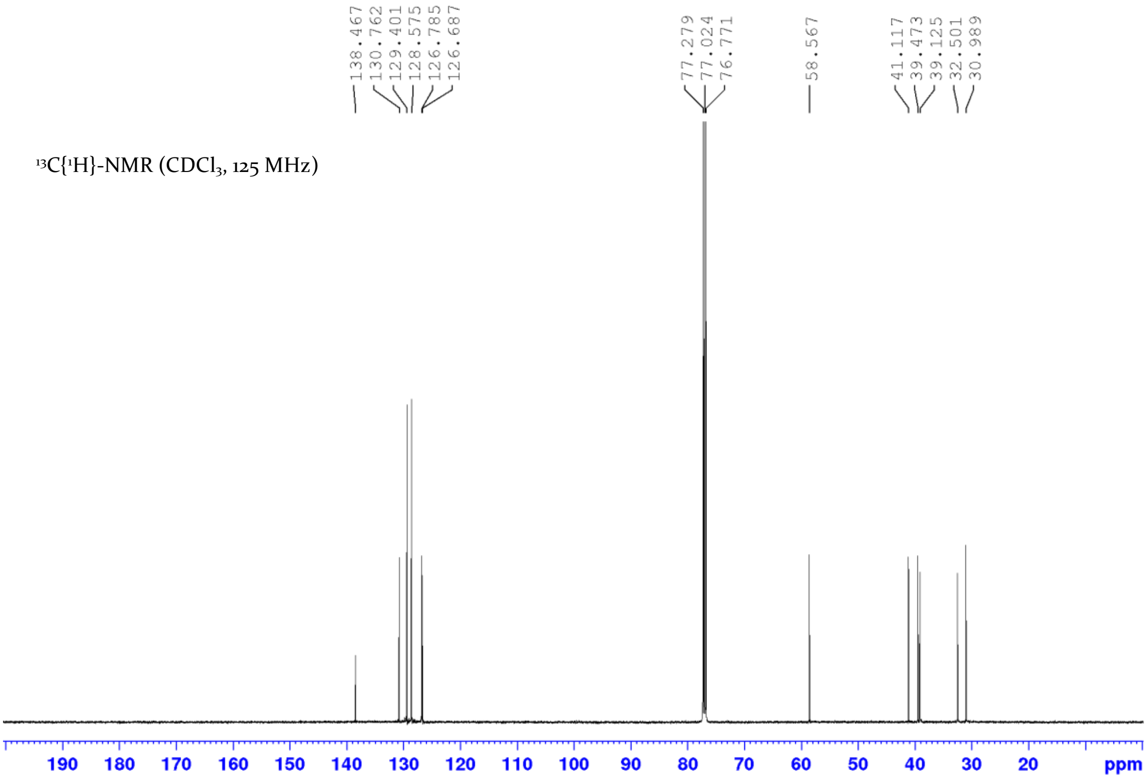
138.467 130.762 129.401 128.575 126.785 126.687 77.279 77.024 76.771 58.567 41.117 39.473 39.125 32.501 30.989
¹³C{¹H}-NMR (CDCl₃, 125 MHz)
190 180 170 160 150 140 130 120 110 100 90 80 70 60 50 40 30 20 ppm

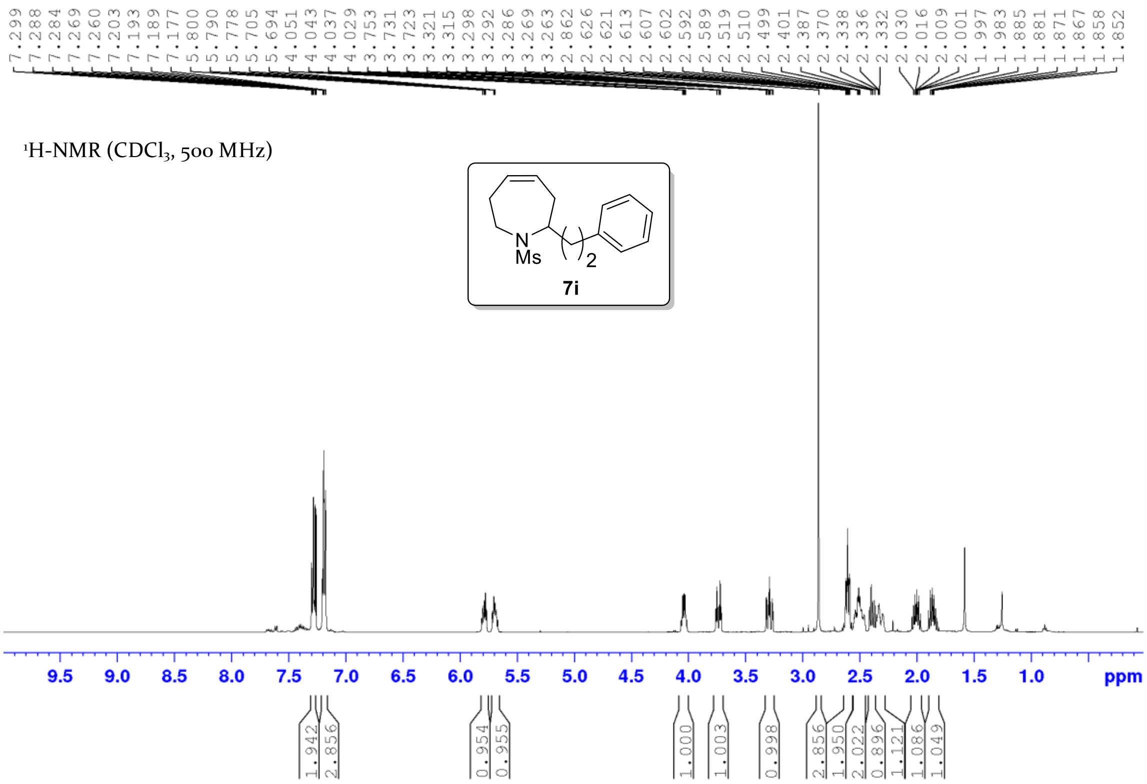
7.299
7.288
7.284
7.269
7.260
7.203
7.193
7.189
7.177
5.800
5.790
5.778
5.705
5.694
4.051
4.043
4.037
4.029
3.753
3.731
3.723
3.321
3.315
3.298
3.292
3.286
3.269
3.263
2.862
2.626
2.621
2.613
2.607
2.602
2.592
2.589
2.519
2.510
2.499
2.401
2.387
2.370
2.338
2.336
2.332
2.030
2.016
2.009
2.001
1.997
1.983
1.885
1.881
1.871
1.867
1.858
1.852
¹H-NMR (CDCl₃, 500 MHz)
N
Ms
2
7i
9.5
9.0
8.5
8.0
7.5
7.0
6.5
6.0
5.5
5.0
4.5
4.0
3.5
3.0
2.5
2.0
1.5
1.0
ppm
1.942
2.856
0.954
0.955
1.000
1.003
0.998
2.856
1.950
2.022
0.896
1.121
1.086
1.049

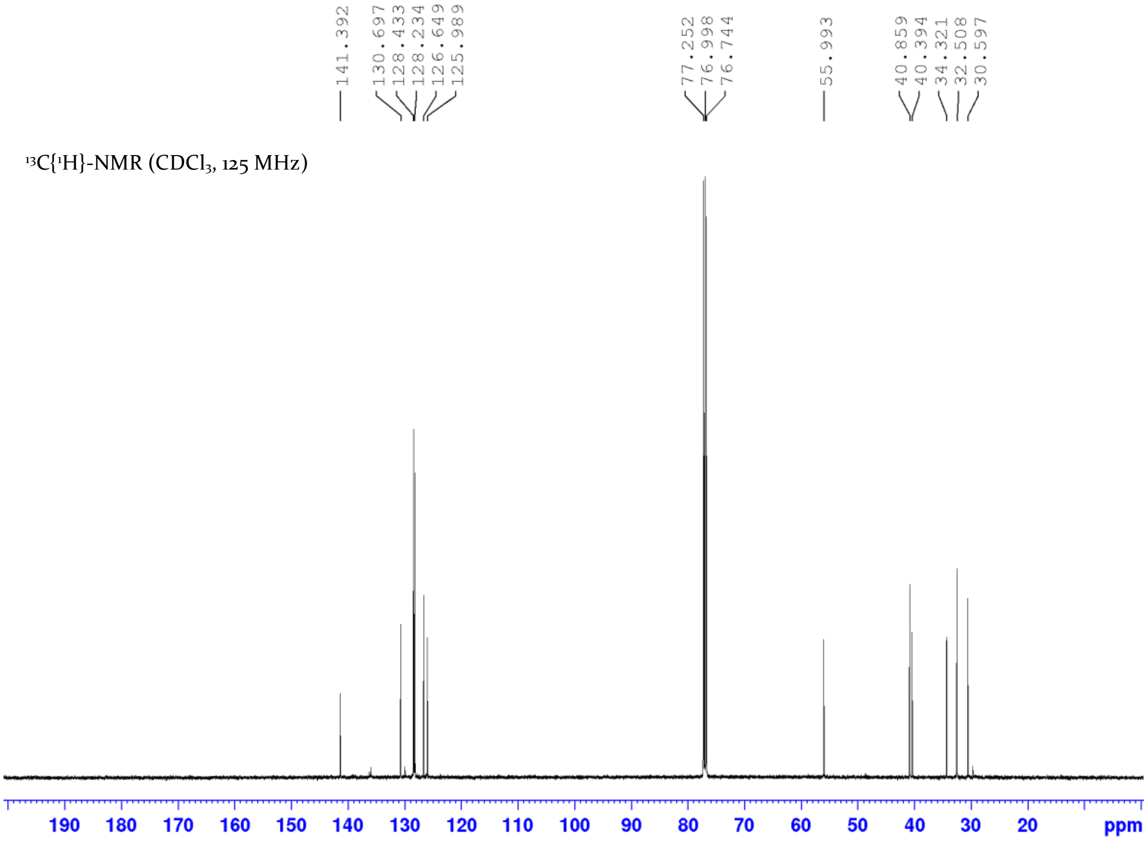
141.392
130.697
128.433
128.234
126.649
125.989
77.252
76.998
76.744
55.993
40.859
40.394
34.321
32.508
30.597
¹³C{¹H}-NMR (CDCl₃, 125 MHz)
190
180
170
160
150
140
130
120
110
100
90
80
70
60
50
40
30
20
ppm

^{1}H-NMR ($CDCl_3$, 500 MHz)

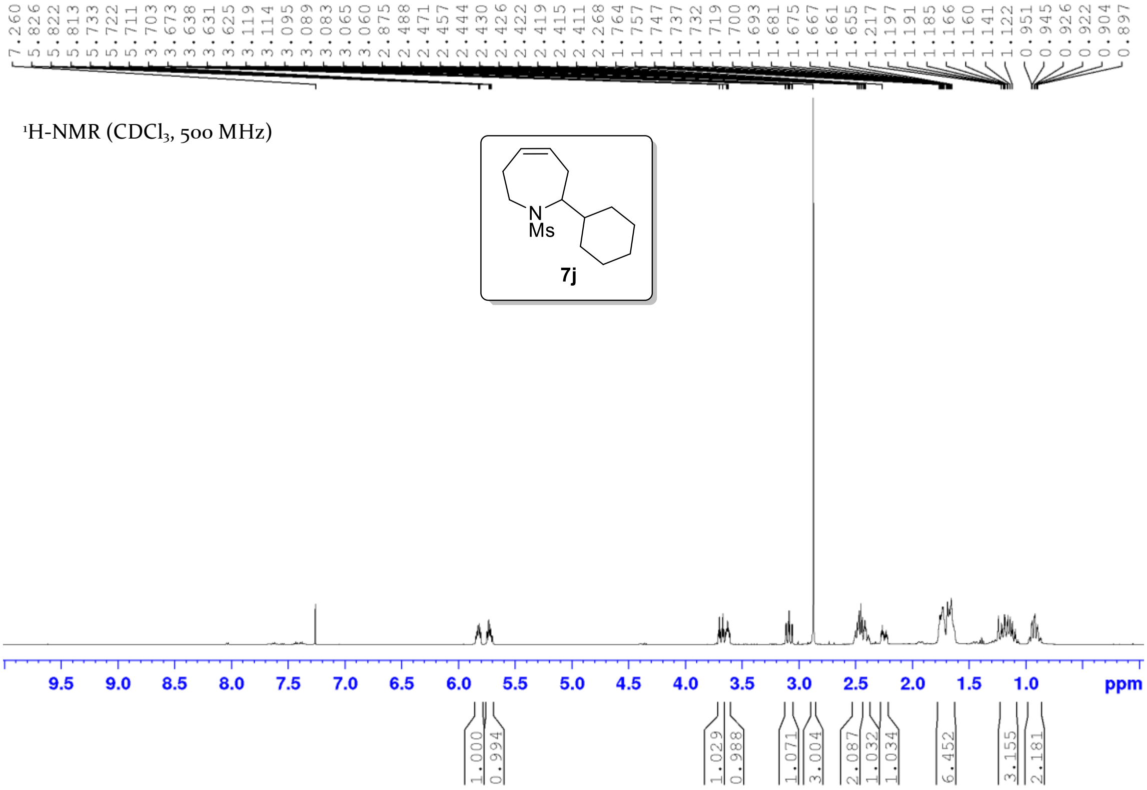

^{13}C{^{1}H}-NMR ($CDCl_3$, 125 MHz)

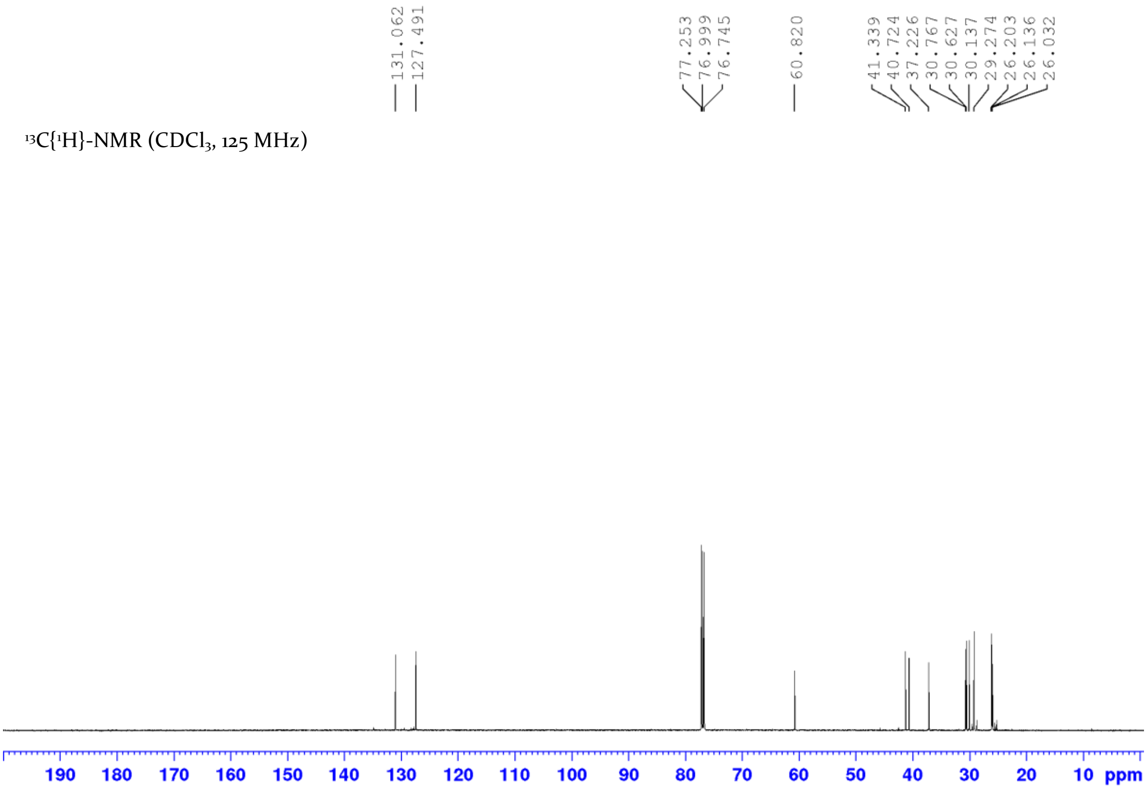

¹H-NMR (CDCl$_3$, 400 MHz)

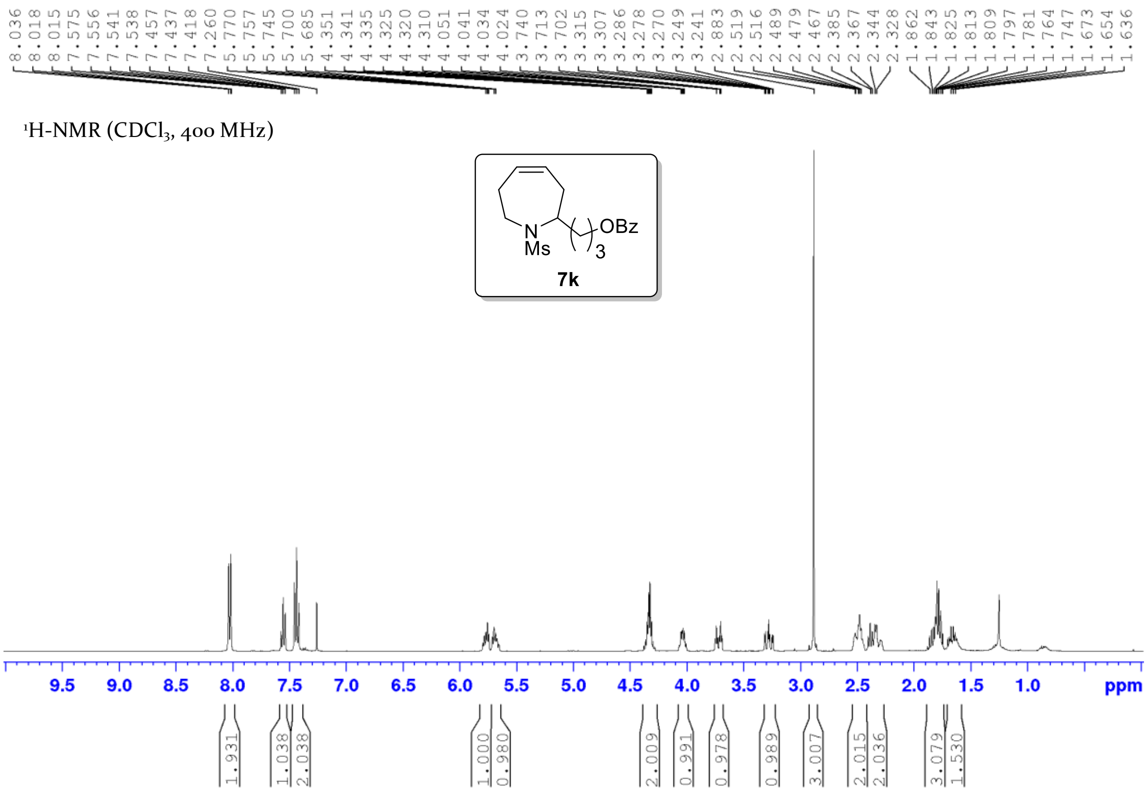

¹³C{¹H}-NMR (CDCl$_3$, 100 MHz)

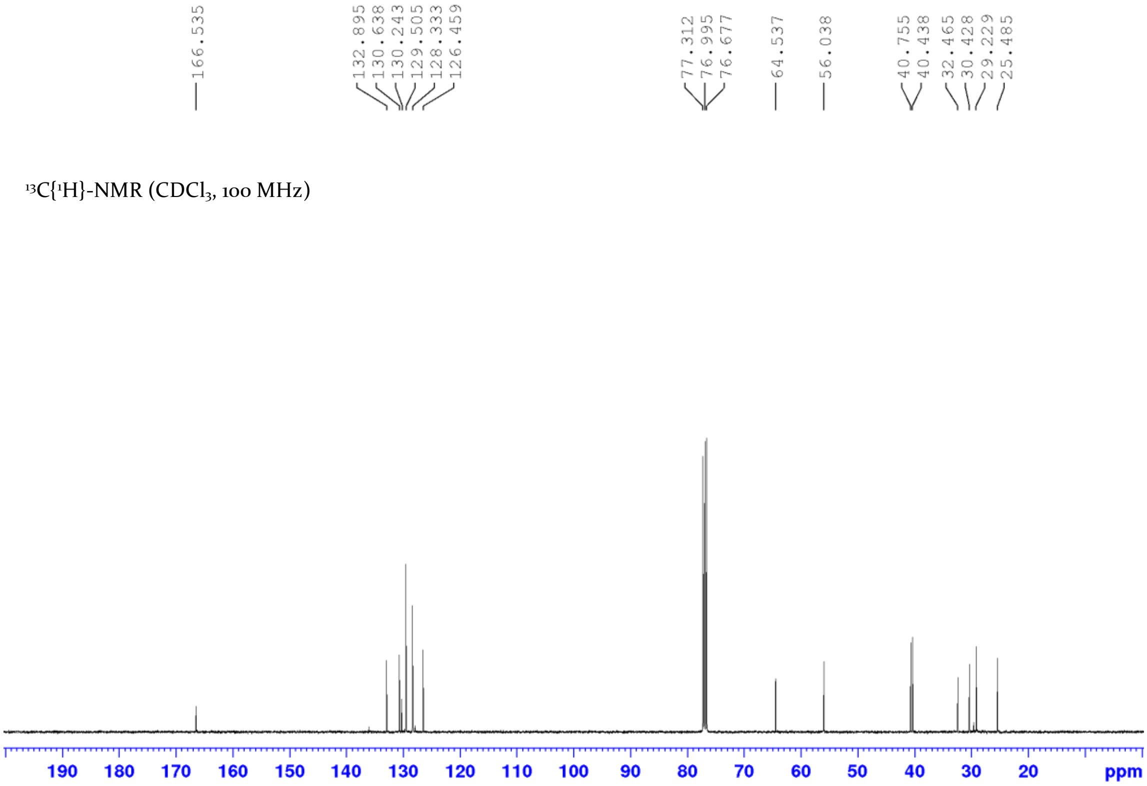

7.592, 7.589, 7.573, 7.569, 7.426, 7.412, 7.408, 7.405, 7.389, 7.371, 7.354, 7.350, 7.260, 5.907, 5.886, 5.881, 5.864, 5.860, 5.843, 5.838, 5.817, 4.981, 4.955, 4.952, 4.951, 4.947, 4.904, 4.106, 4.088, 4.070, 4.053, 3.169, 3.165, 3.162, 3.148, 3.139, 3.132, 3.118, 3.111, 2.700, 2.693, 2.661, 2.654, 2.521, 2.491, 2.482, 2.452, 1.222, 1.205, 1.187

^{1}H-NMR ($CDCl_3$, 400 MHz)

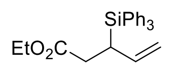

19

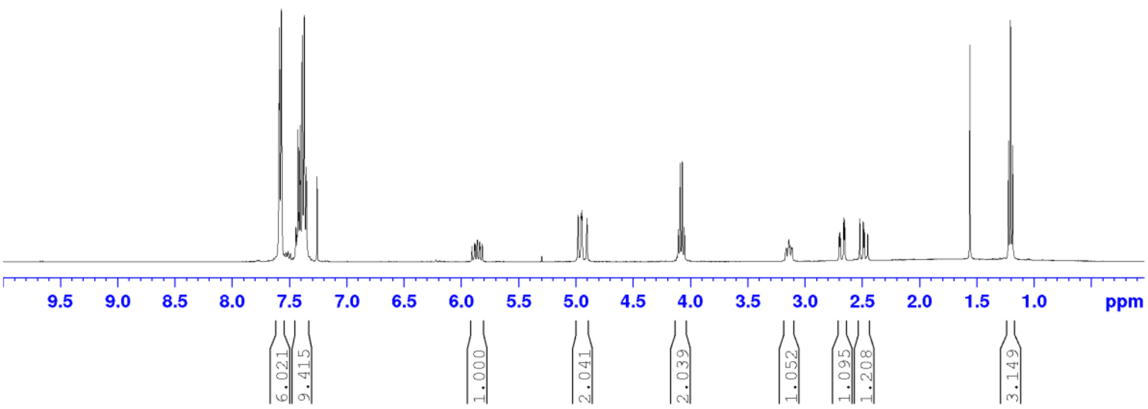

173.033, 137.222, 136.071, 133.108, 129.699, 127.933, 114.662, 77.316, 76.999, 76.681, 60.380, 34.452, 27.996, 14.249

^{13}C{^{1}H}-NMR ($CDCl_3$, 100 MHz)

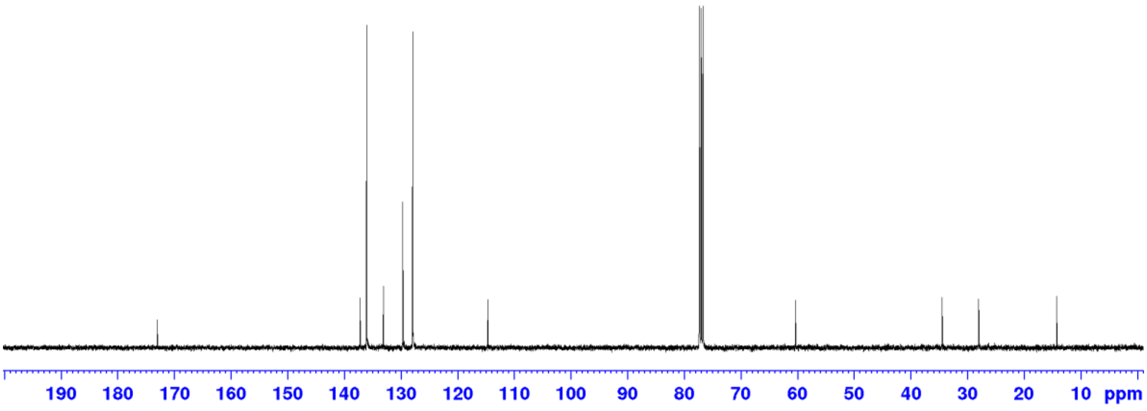

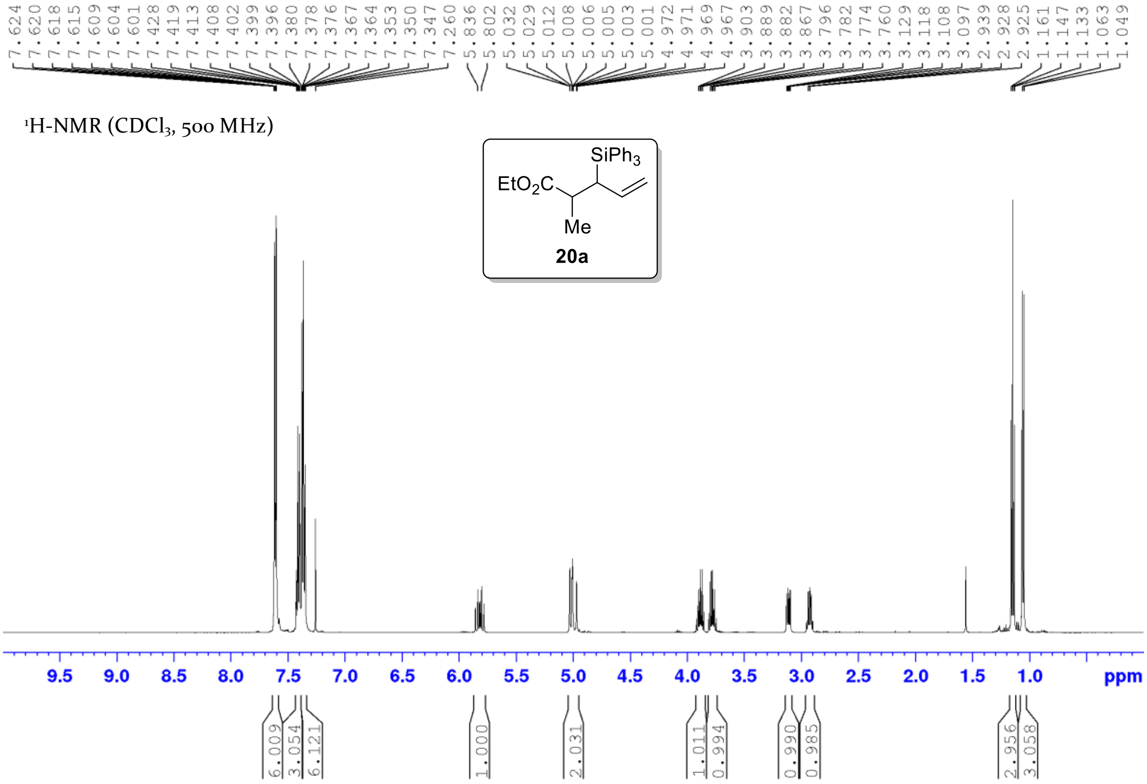

^{1}H-NMR ($CDCl_3$, 500 MHz)

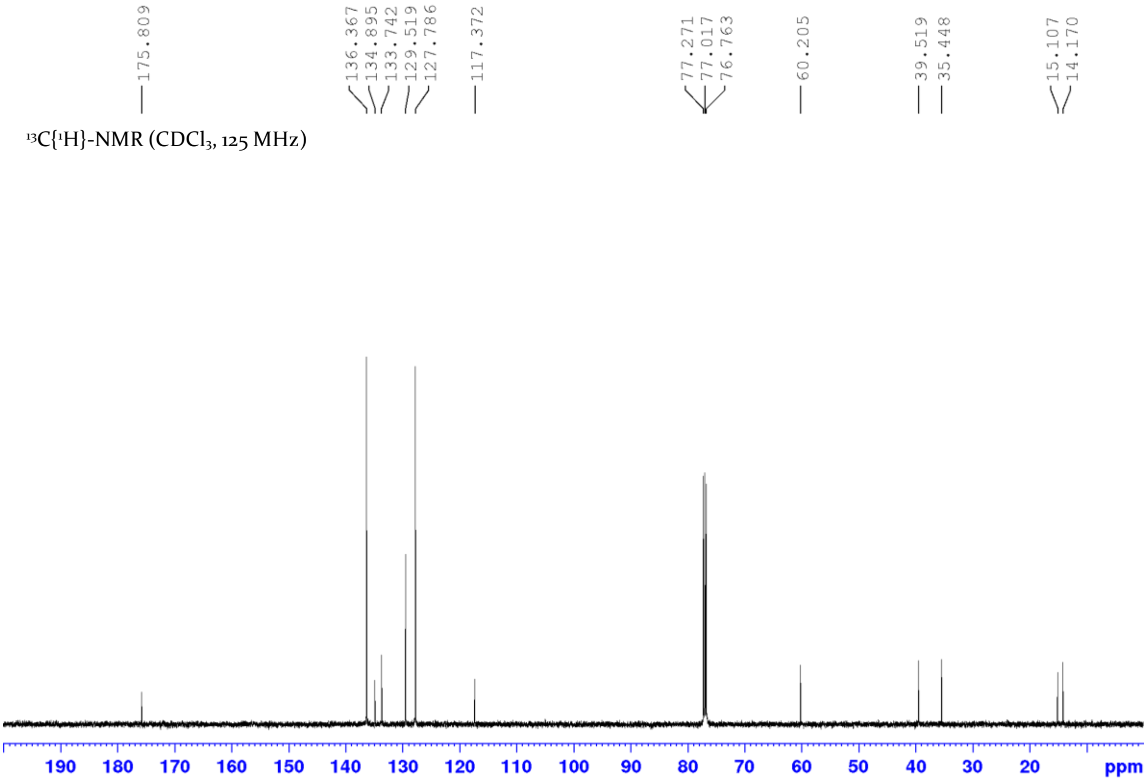

^{13}C{^{1}H}-NMR ($CDCl_3$, 125 MHz)

^{1}H-NMR ($CDCl_3$, 400 MHz)

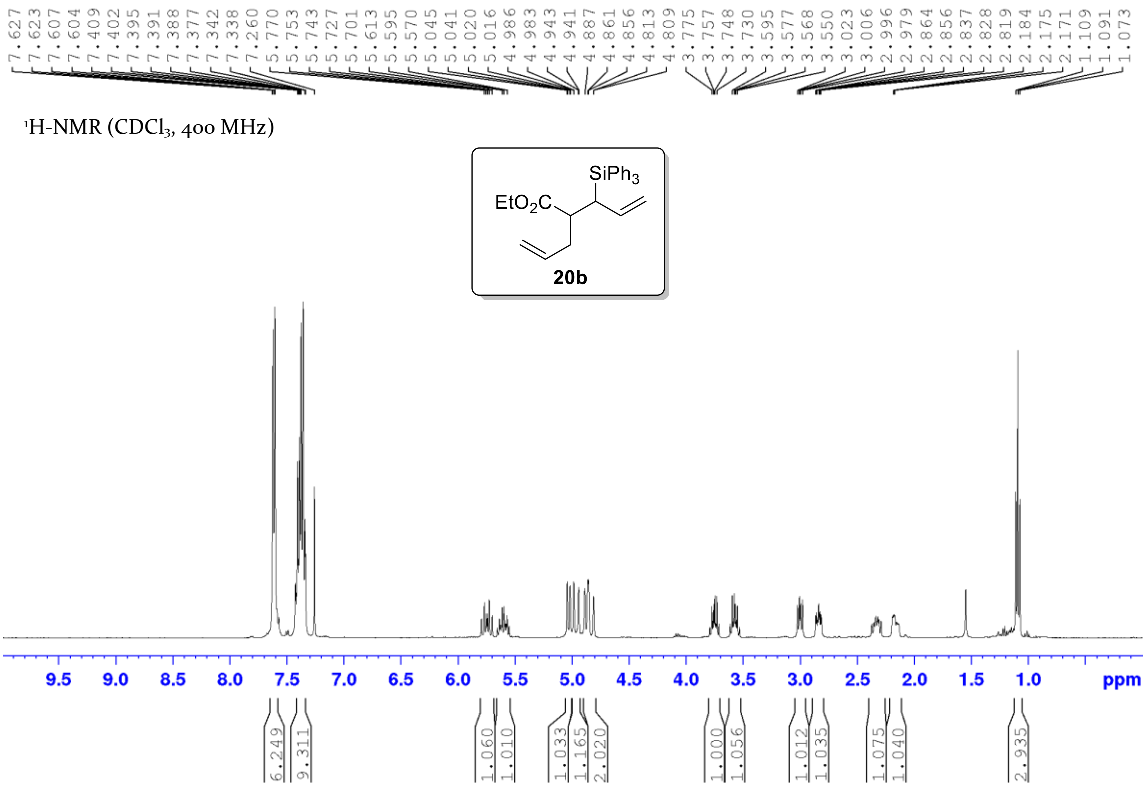

^{13}C{^{1}H}-NMR ($CDCl_3$, 100 MHz)

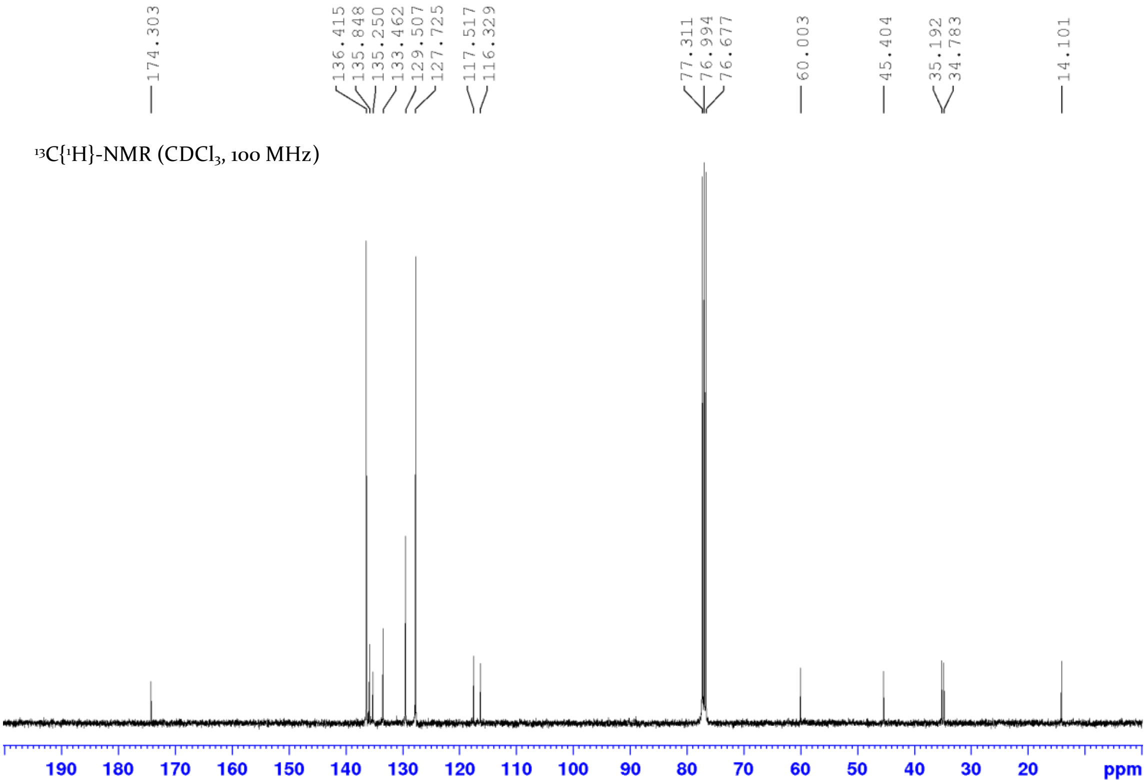

^{1}H-NMR ($CDCl_3$, 500 MHz)

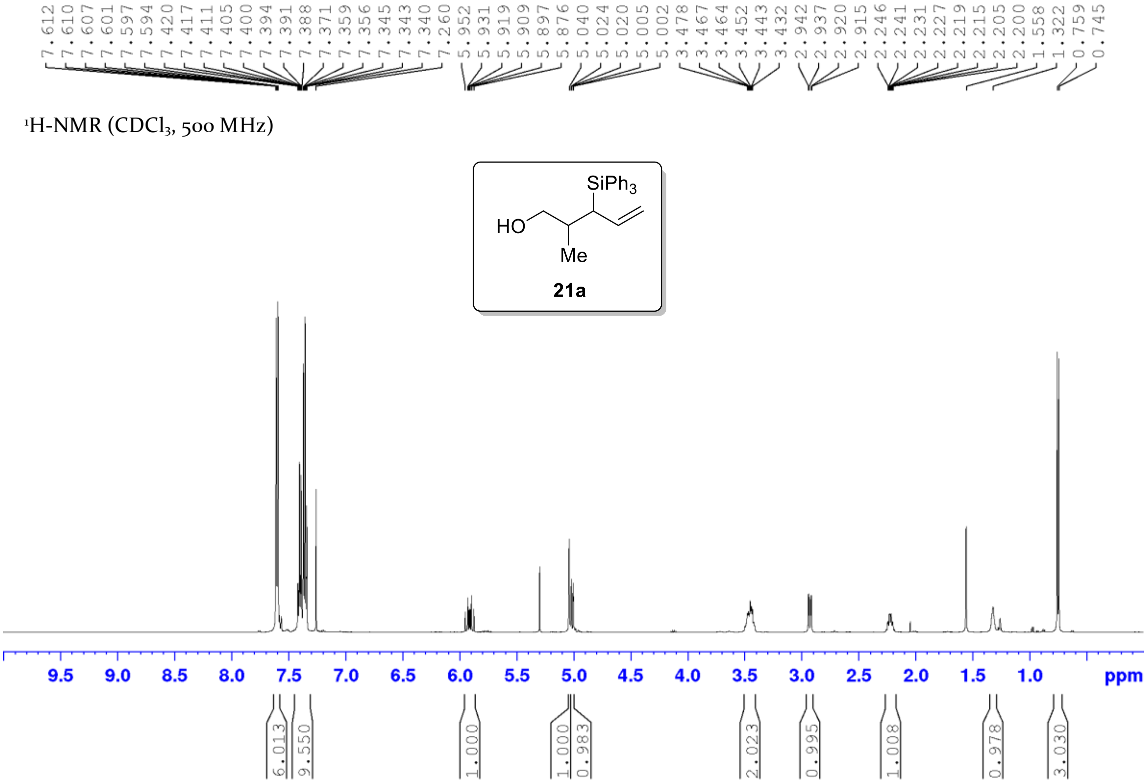

^{13}C{^{1}H}-NMR ($CDCl_3$, 125 MHz)

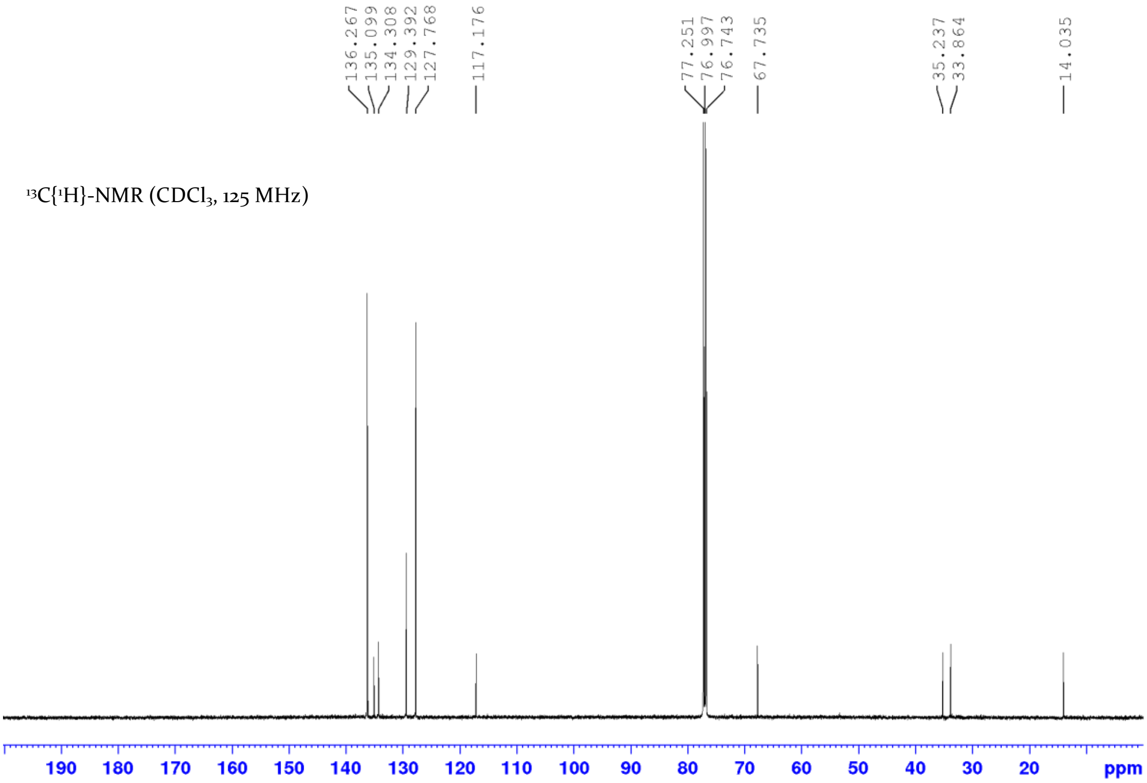

¹H-NMR (CDCl$_3$, 400 MHz)

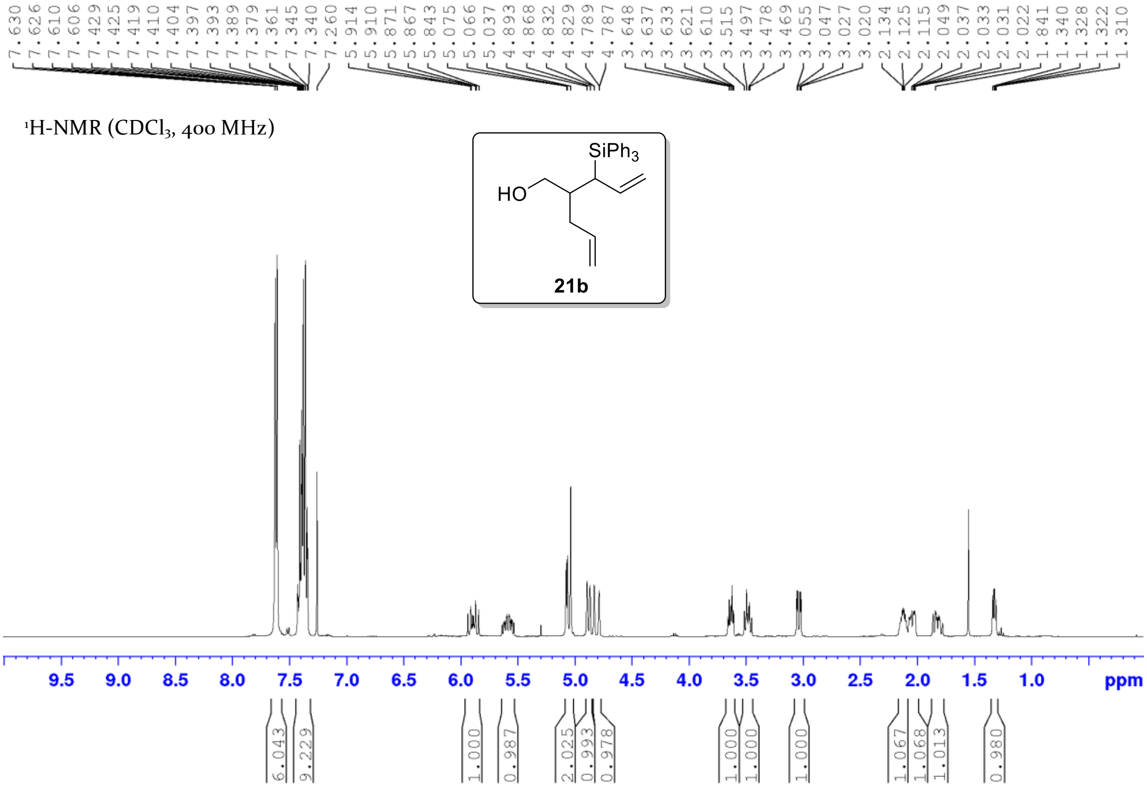

¹³C{¹H}-NMR (CDCl$_3$, 100 MHz)

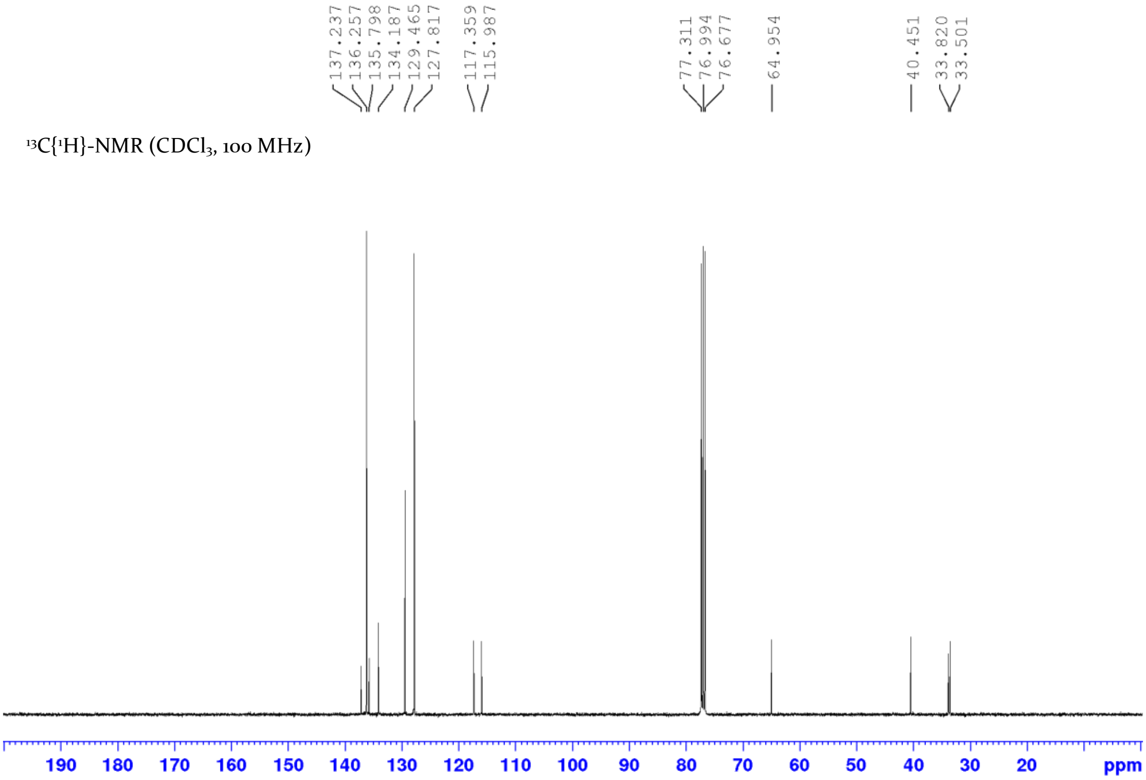

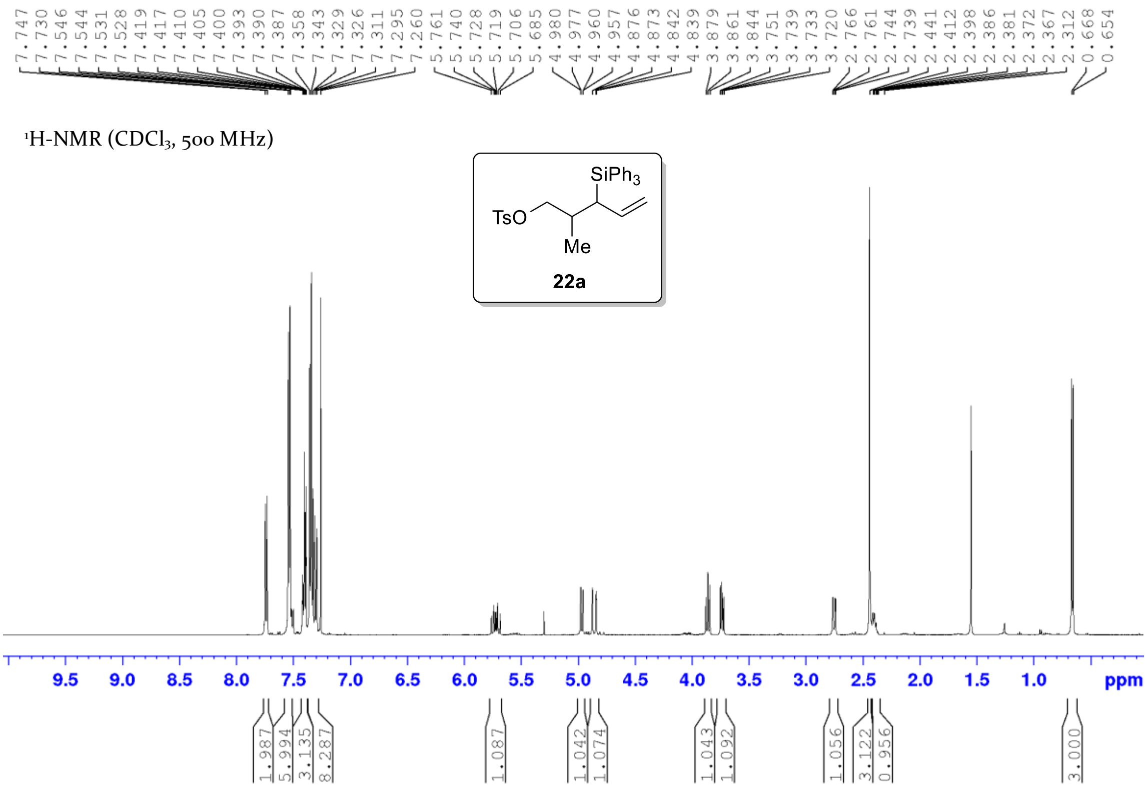

^{1}H-NMR ($CDCl_3$, 500 MHz)

22a

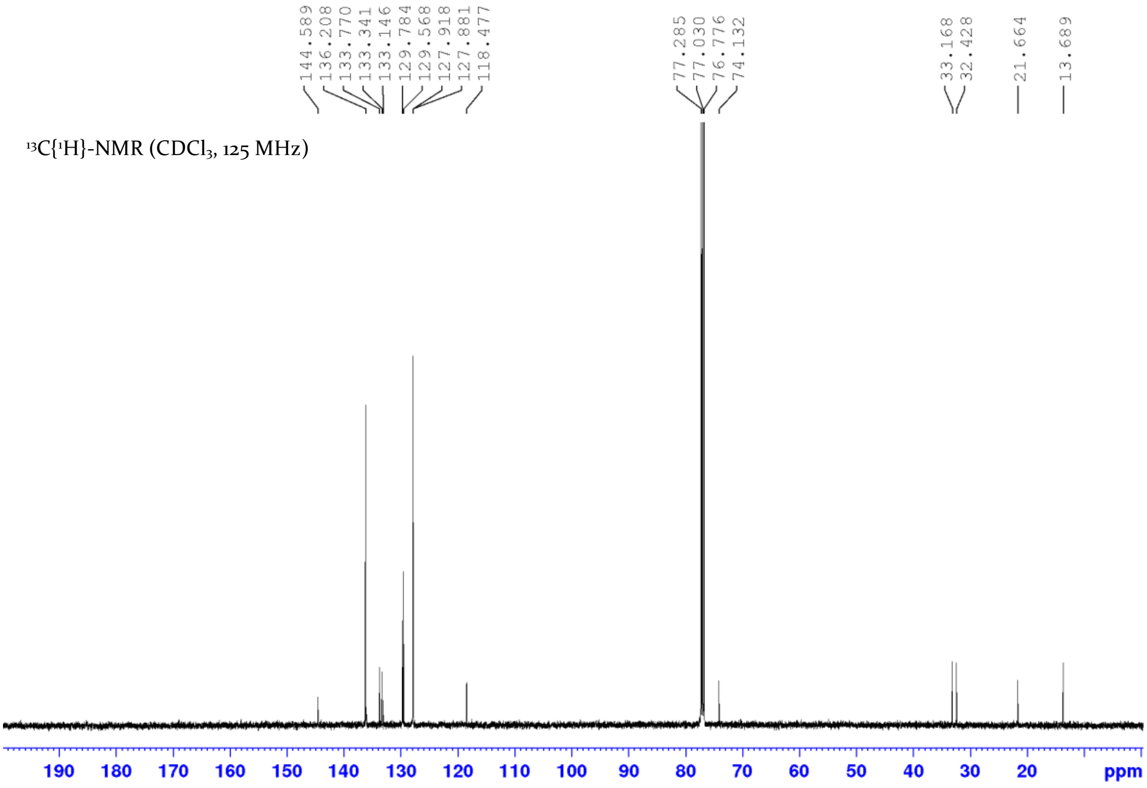

^{13}C{^{1}H}-NMR ($CDCl_3$, 125 MHz)

¹H-NMR (CDCl$_3$, 400 MHz)

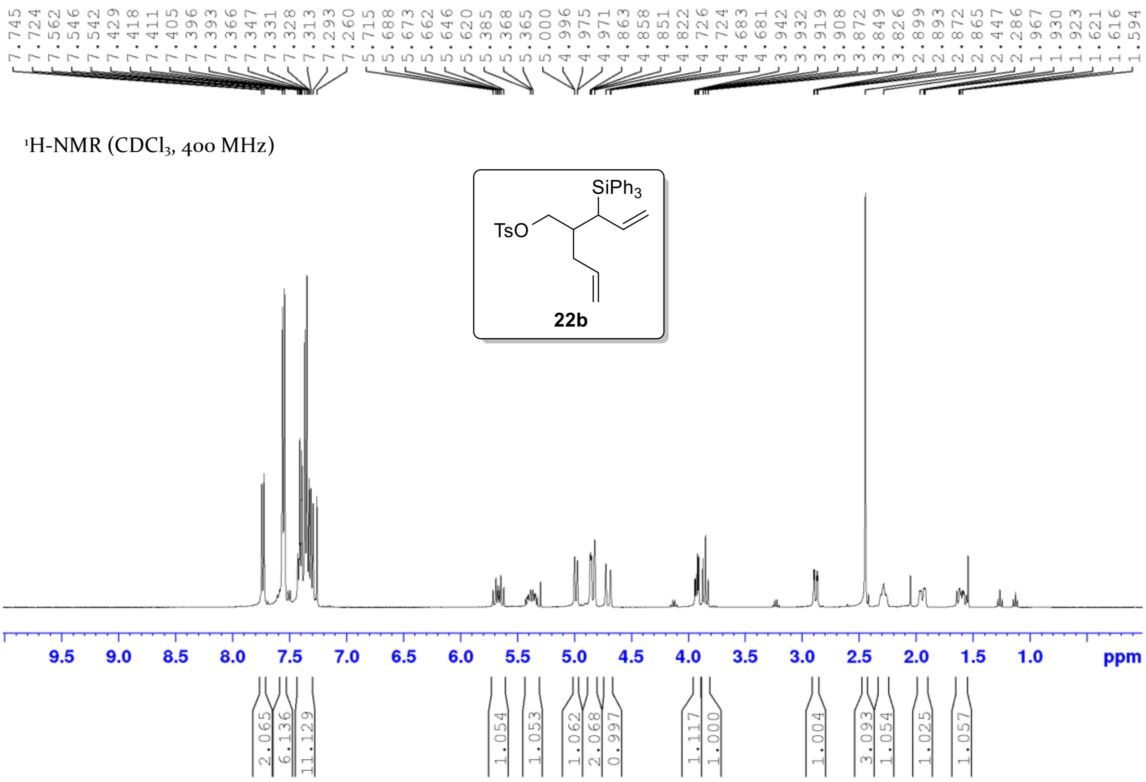

¹³C{¹H}-NMR (CDCl$_3$, 100 MHz)

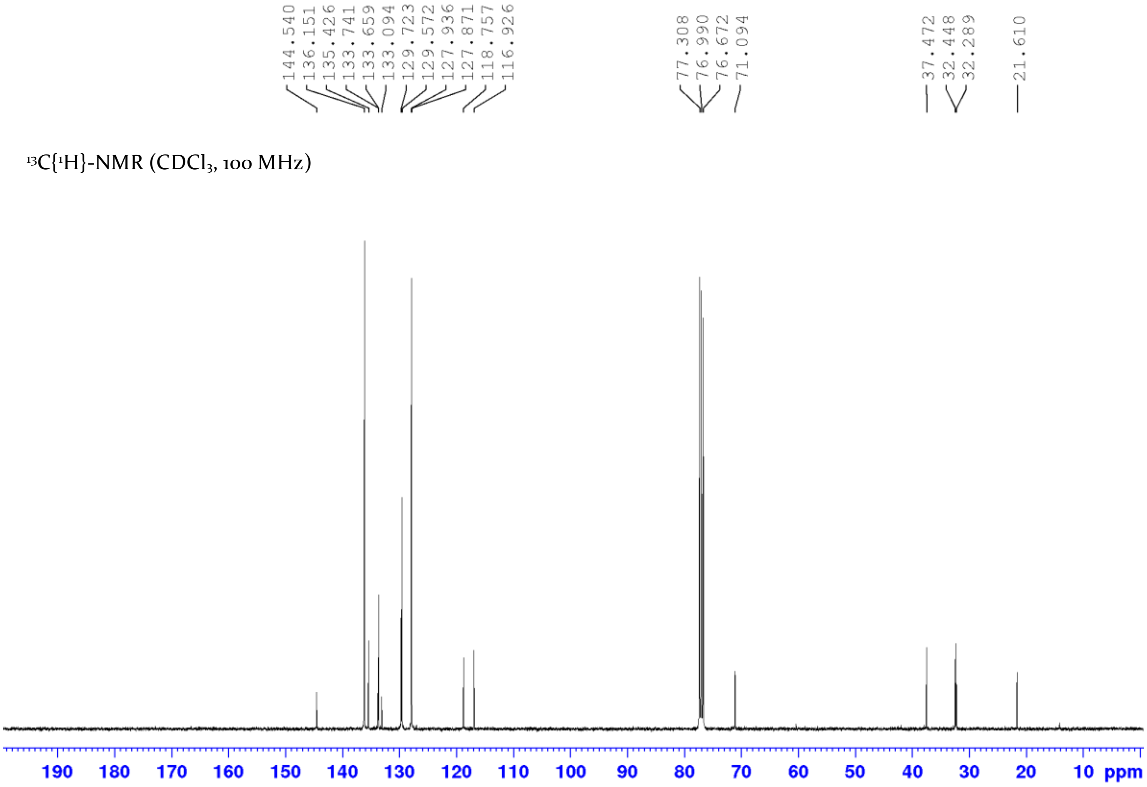

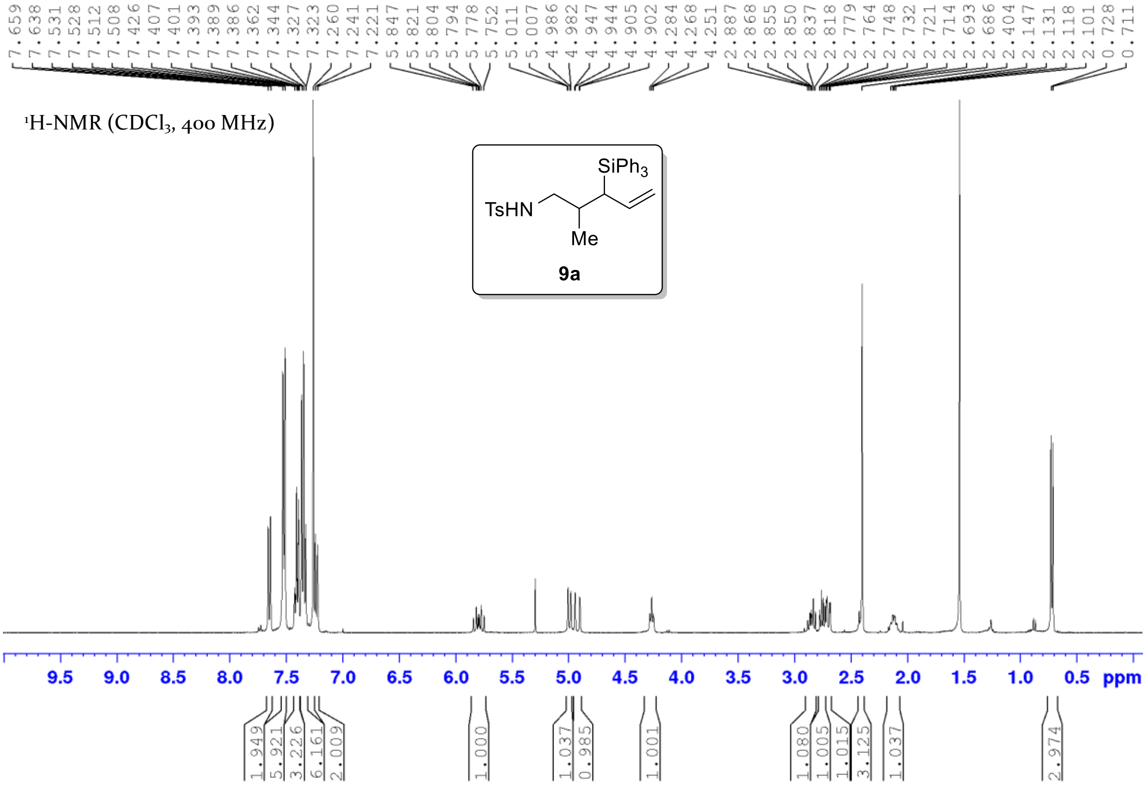
1H-NMR (CDCl3, 400 MHz)
SiPh3
TsHN
Me
9a
7.659 7.638 7.531 7.528 7.512 7.508 7.426 7.407 7.401 7.393 7.389 7.386 7.362 7.344 7.327 7.323 7.260 7.241 7.221 5.847 5.821 5.804 5.794 5.778 5.752 5.011 5.007 4.986 4.982 4.947 4.944 4.905 4.902 4.284 4.268 4.251 2.887 2.868 2.855 2.850 2.837 2.818 2.779 2.764 2.748 2.732 2.721 2.714 2.693 2.686 2.404 2.147 2.131 2.118 2.101 0.728 0.711
1.949 5.921 3.226 6.161 2.009 1.000 1.037 0.985 1.001 1.080 1.005 1.015 3.125 1.037 2.974
9.5 9.0 8.5 8.0 7.5 7.0 6.5 6.0 5.5 5.0 4.5 4.0 3.5 3.0 2.5 2.0 1.5 1.0 0.5 ppm

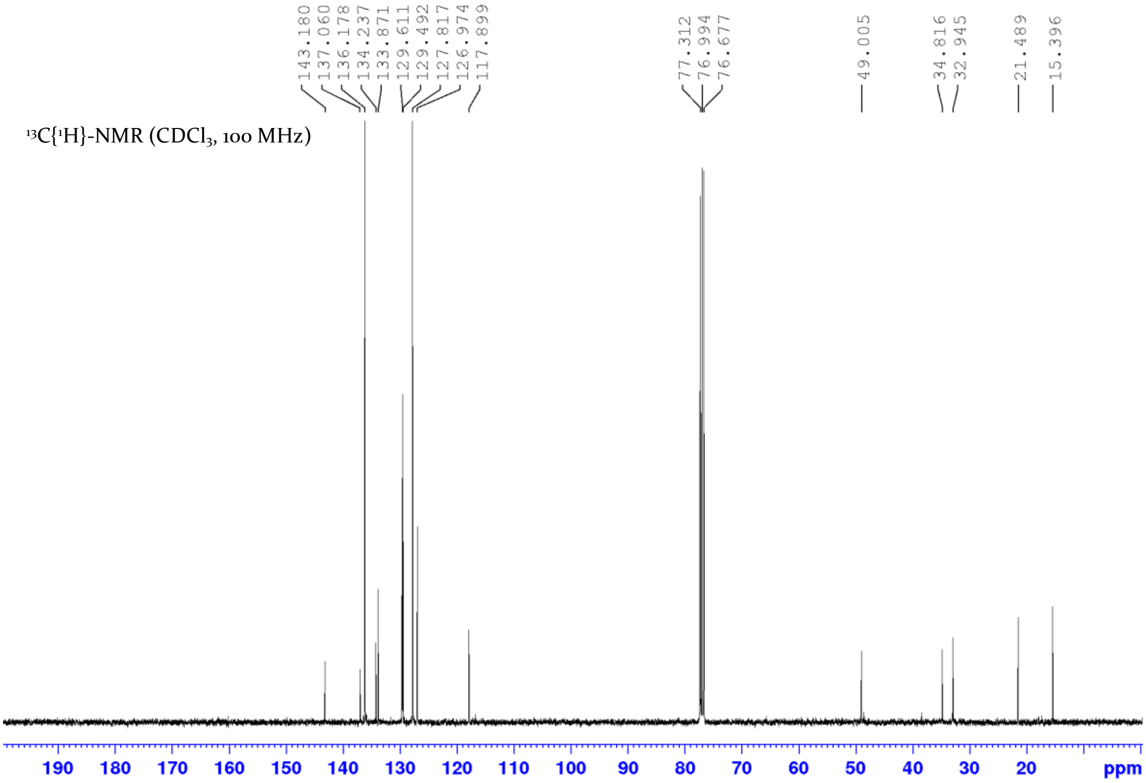
13C{1H}-NMR (CDCl3, 100 MHz)
143.180 137.060 136.178 134.237 133.871 129.611 129.492 127.817 126.974 117.899 77.312 76.994 76.677 49.005 34.816 32.945 21.489 15.396
190 180 170 160 150 140 130 120 110 100 90 80 70 60 50 40 30 20 ppm

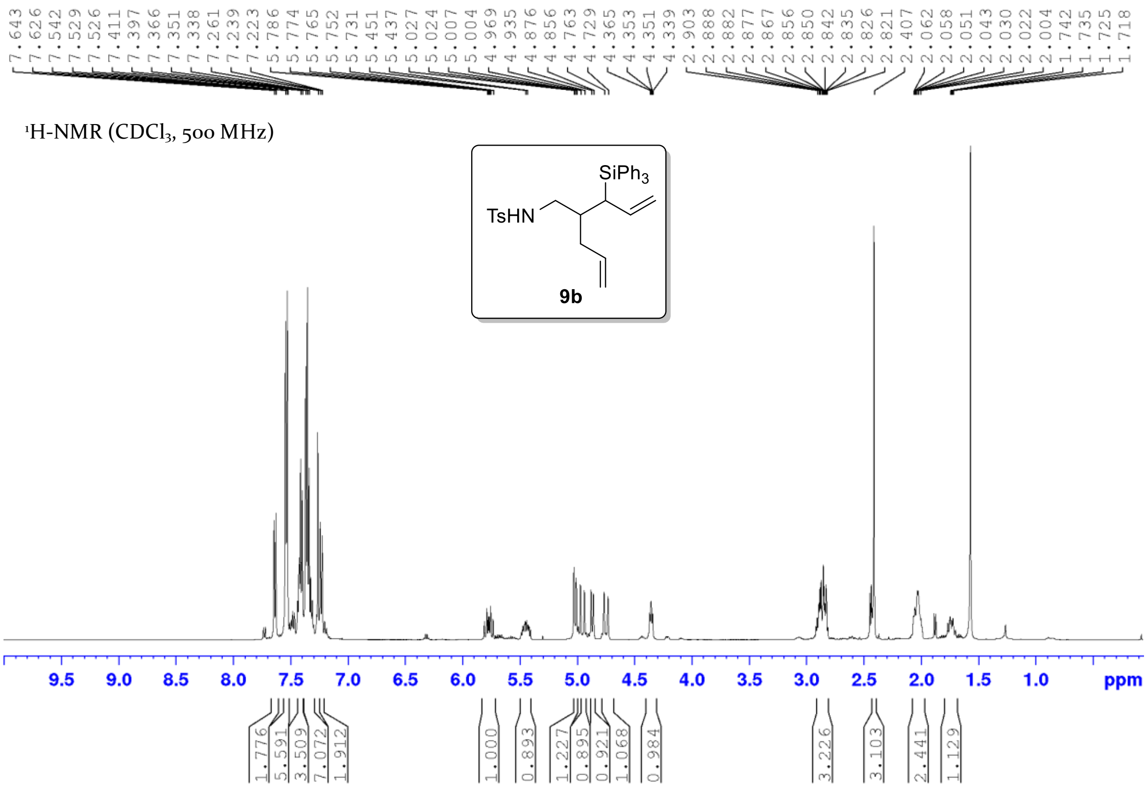

^{1}H-NMR (CDCl$_3$, 500 MHz)

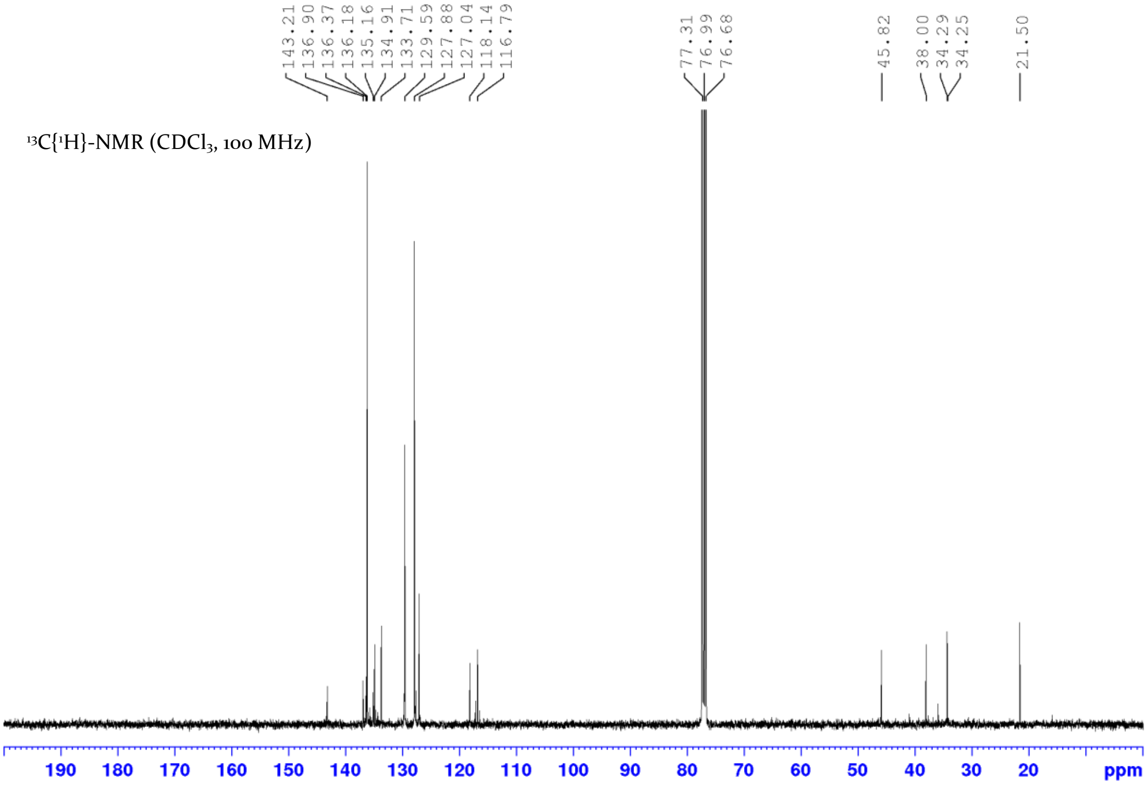

^{13}C{^{1}H}-NMR (CDCl$_3$, 100 MHz)

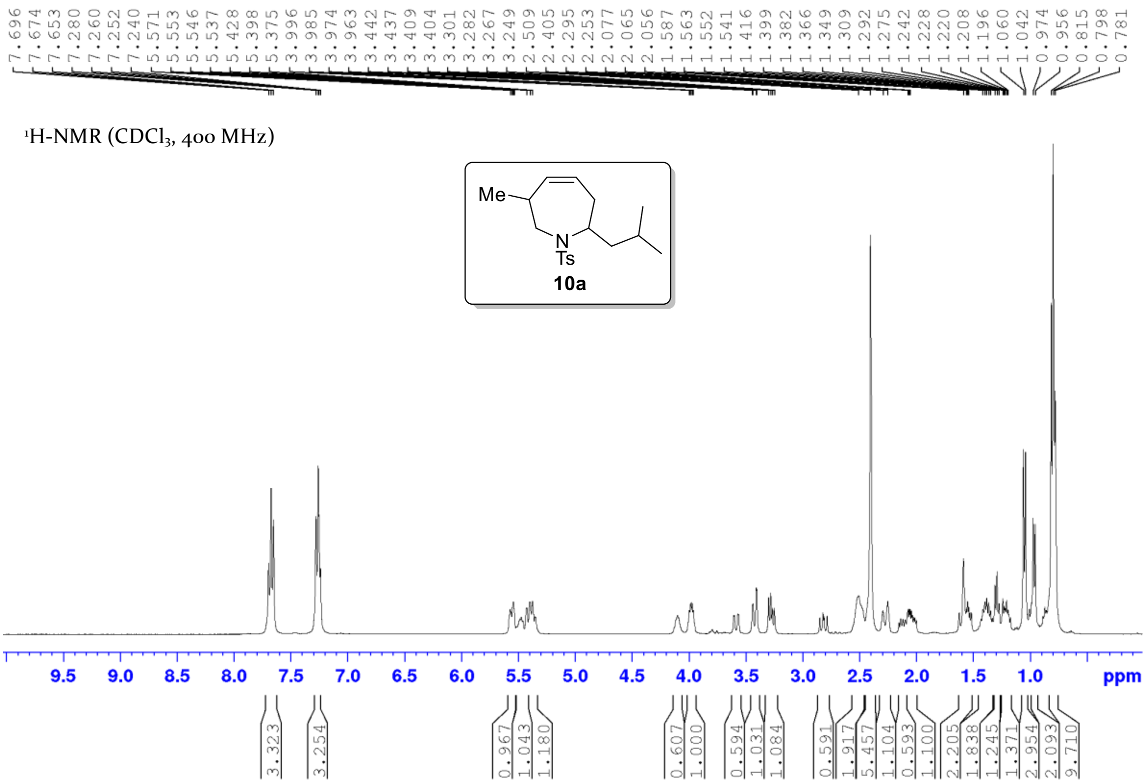

^{1}H-NMR ($CDCl_3$, 400 MHz)

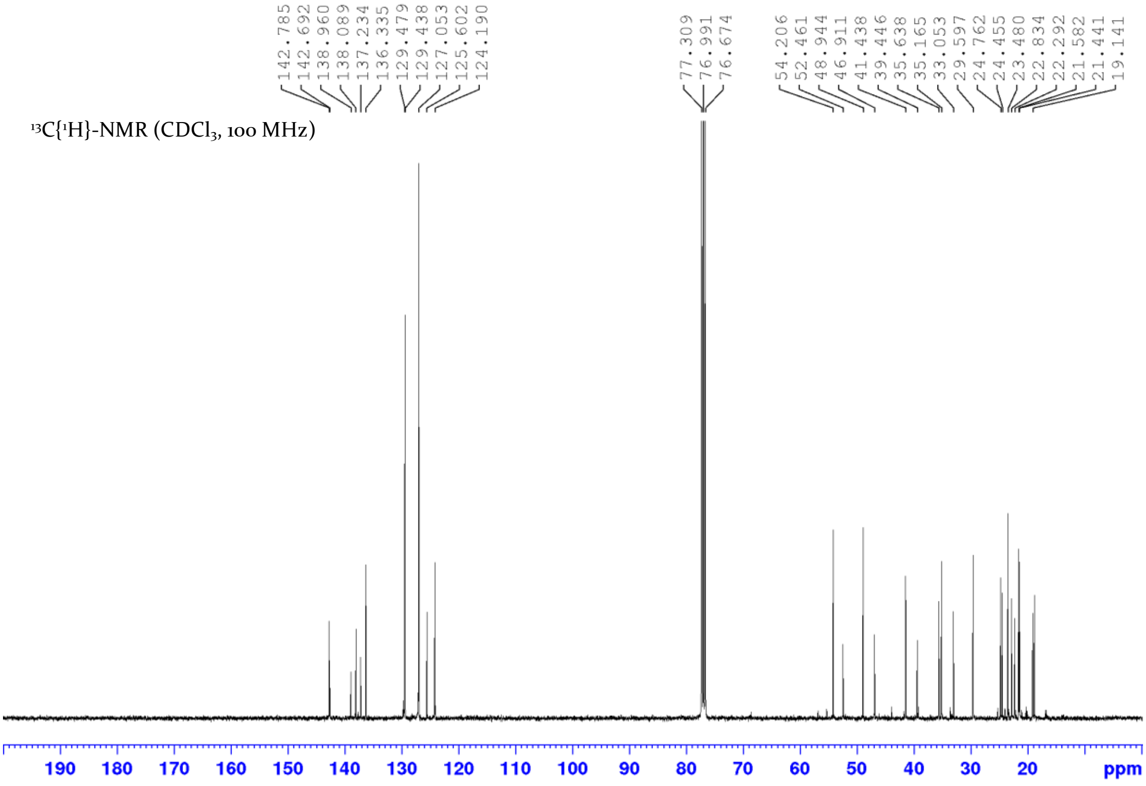

^{13}C{^{1}H}-NMR ($CDCl_3$, 100 MHz)

^{1}H-NMR ($CDCl_3$, 500 MHz)

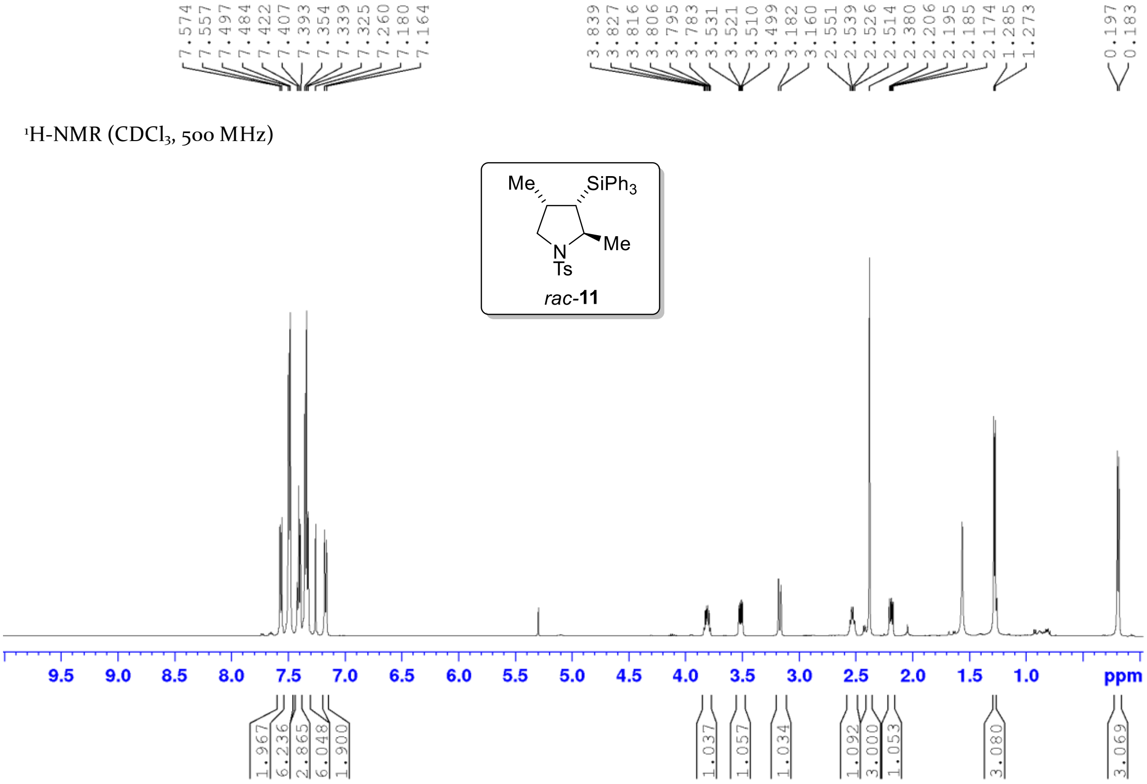

^{13}C{^{1}H}-NMR ($CDCl_3$, 100 MHz)

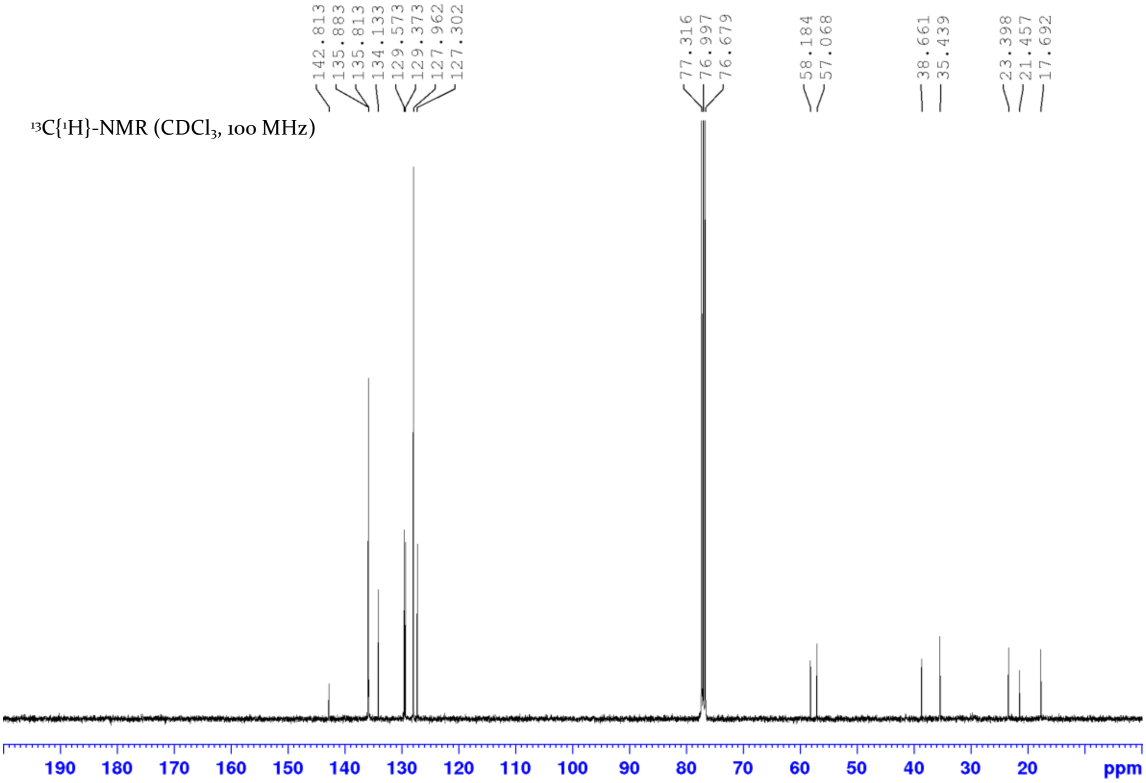

^{1}H-GOESY NMR (CDCl3, 500 MHz)

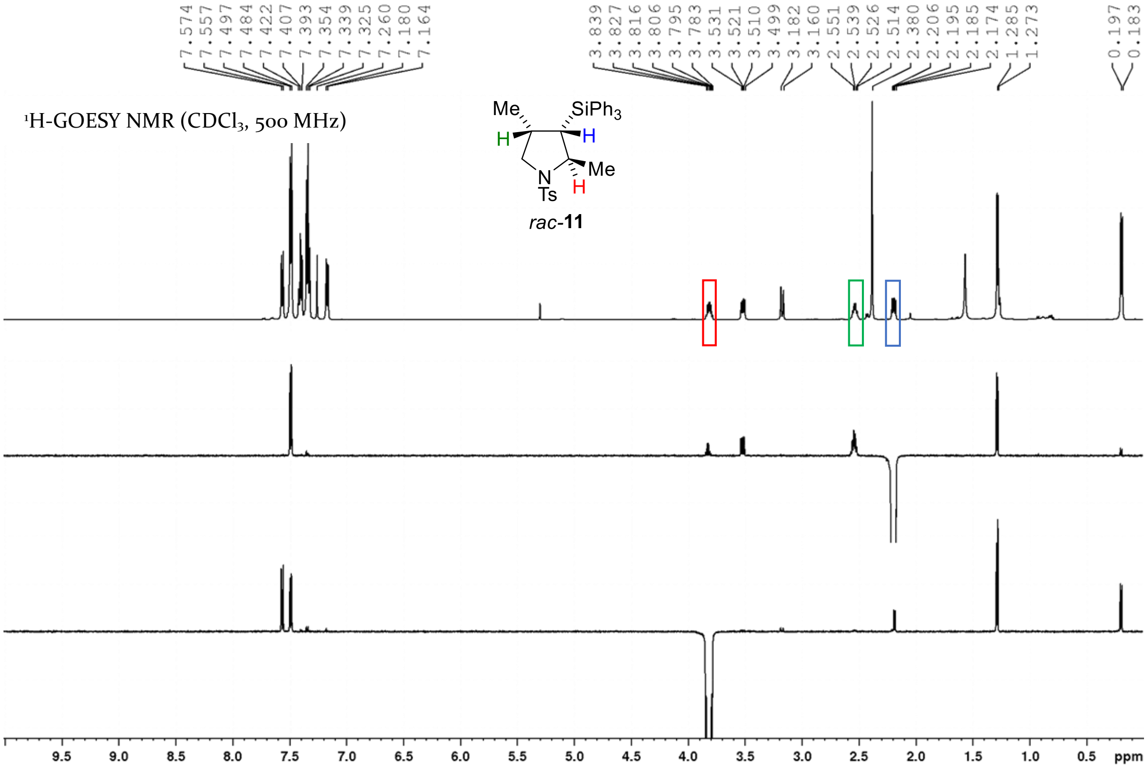

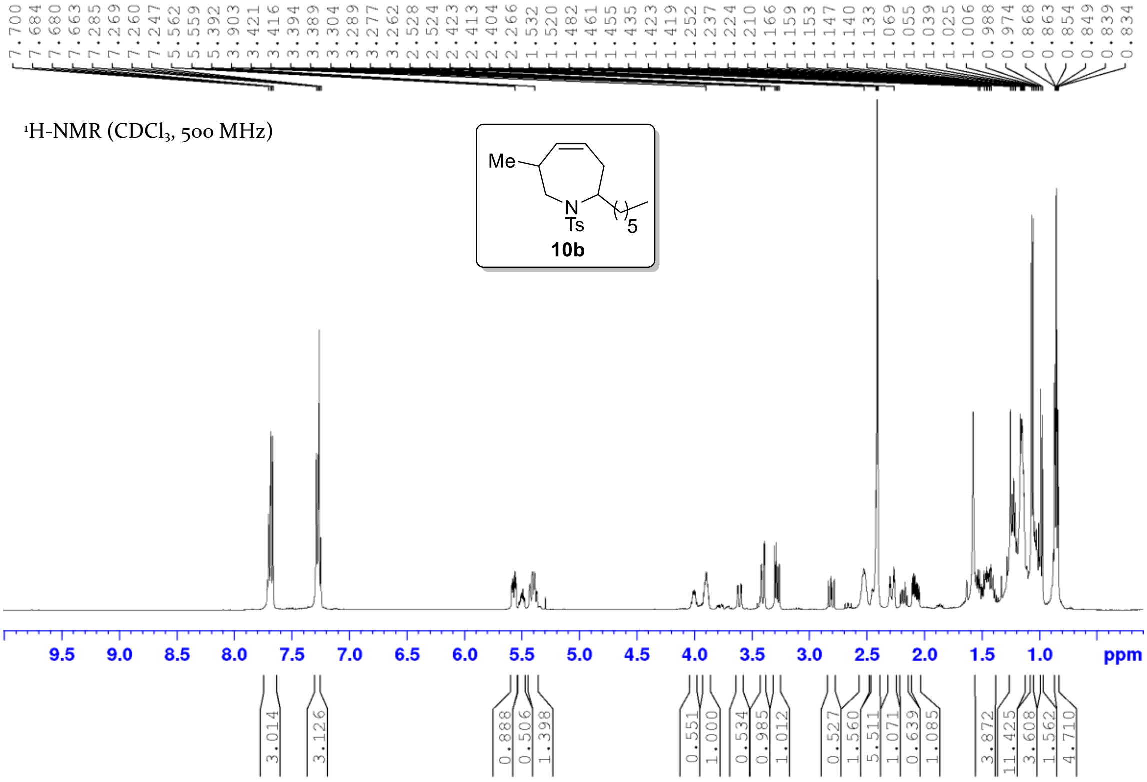

^{1}H-NMR (CDCl$_3$, 500 MHz)

10b

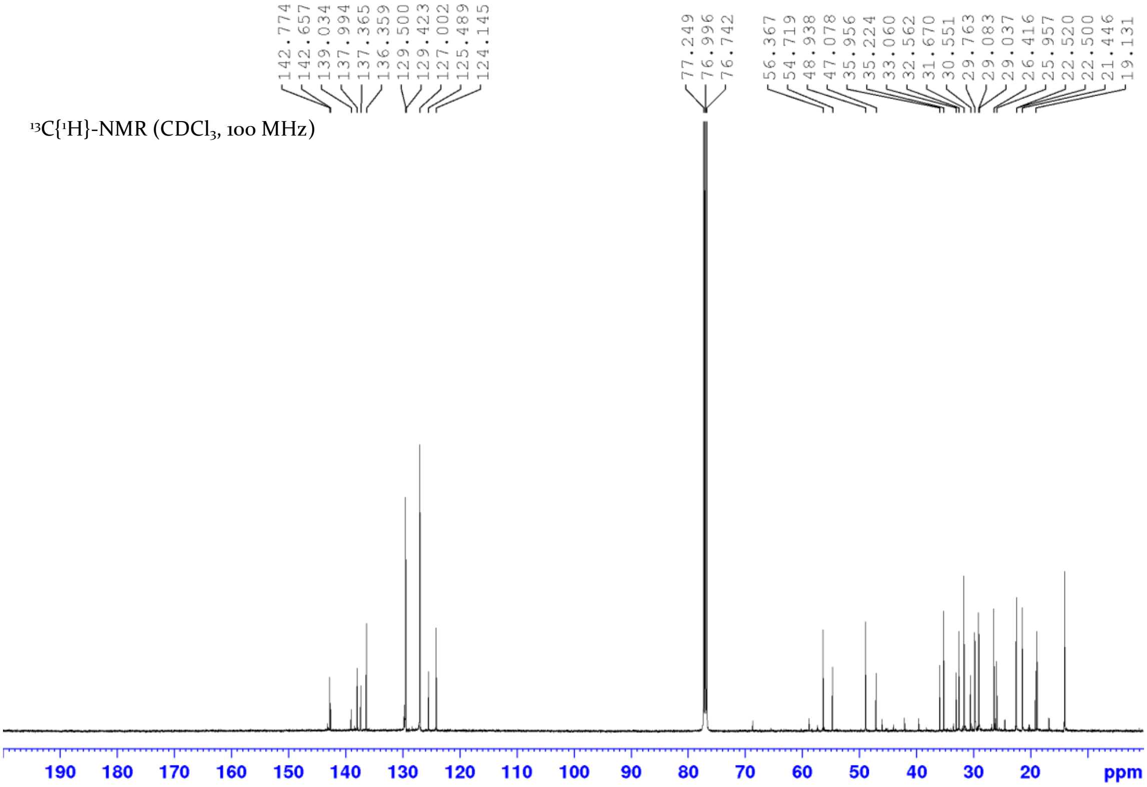

^{13}C{^{1}H}-NMR (CDCl$_3$, 100 MHz)

^{1}H-NMR ($CDCl_3$, 500 MHz)

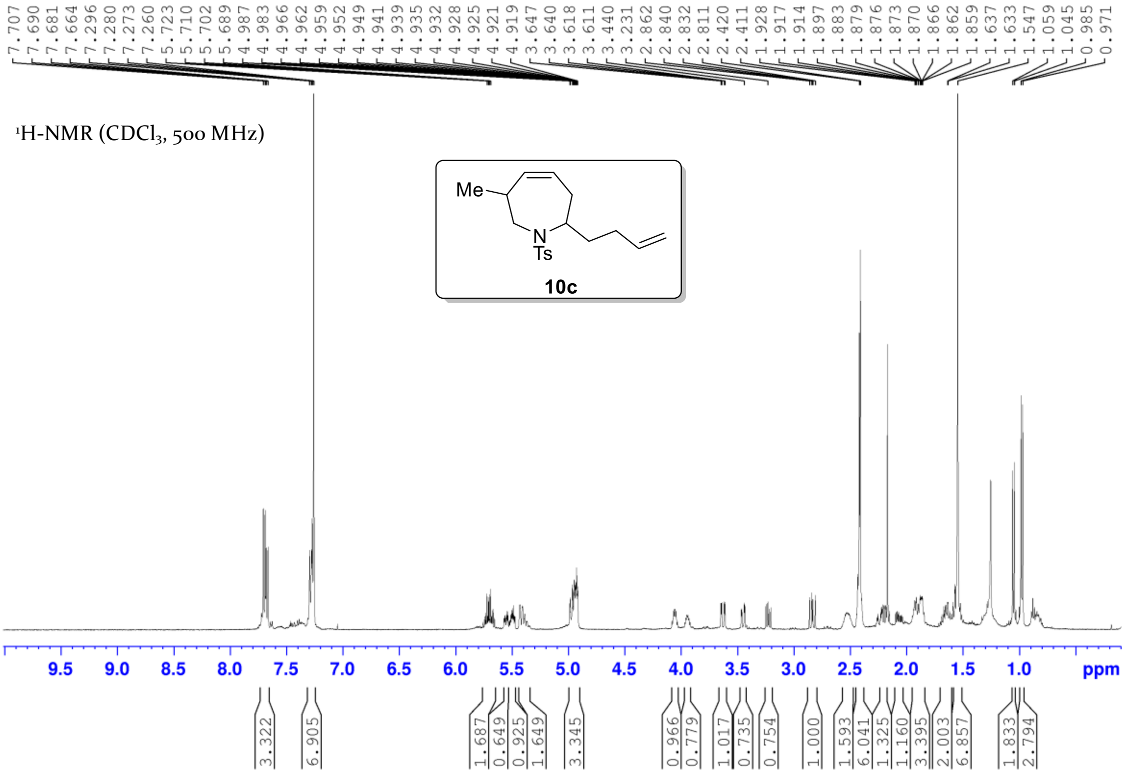

^{13}C{^{1}H}-NMR ($CDCl_3$, 125 MHz)

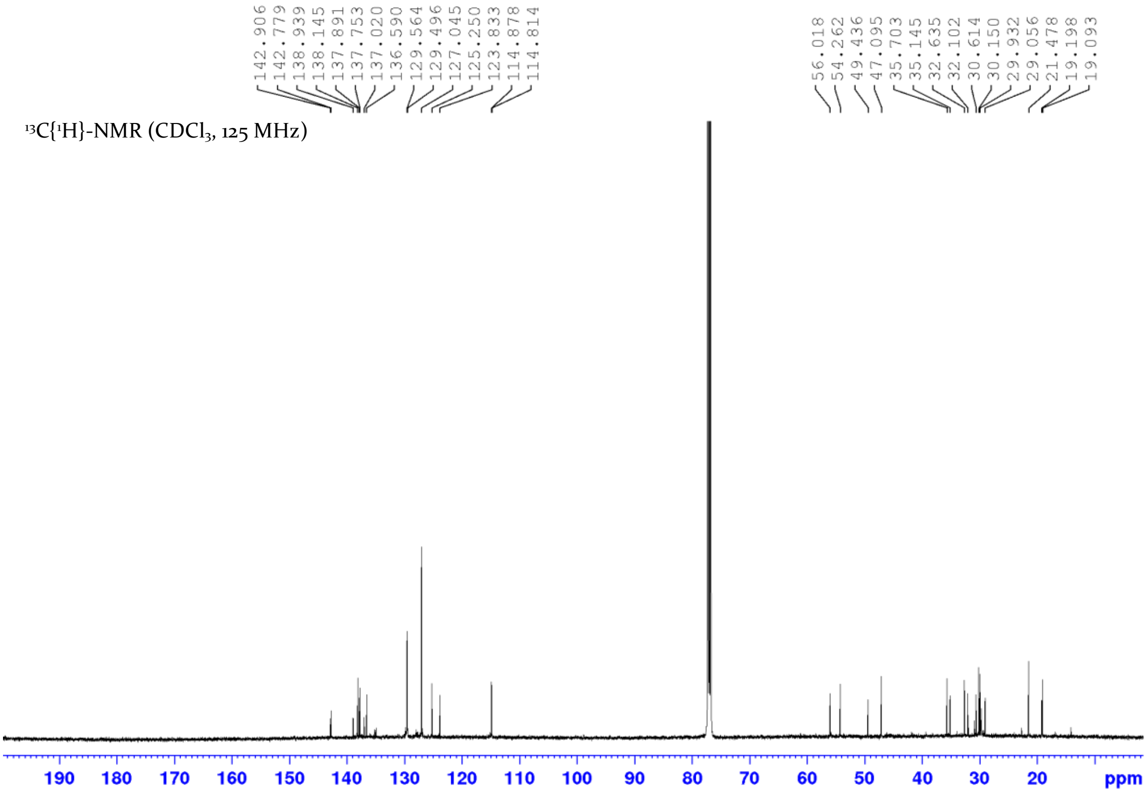

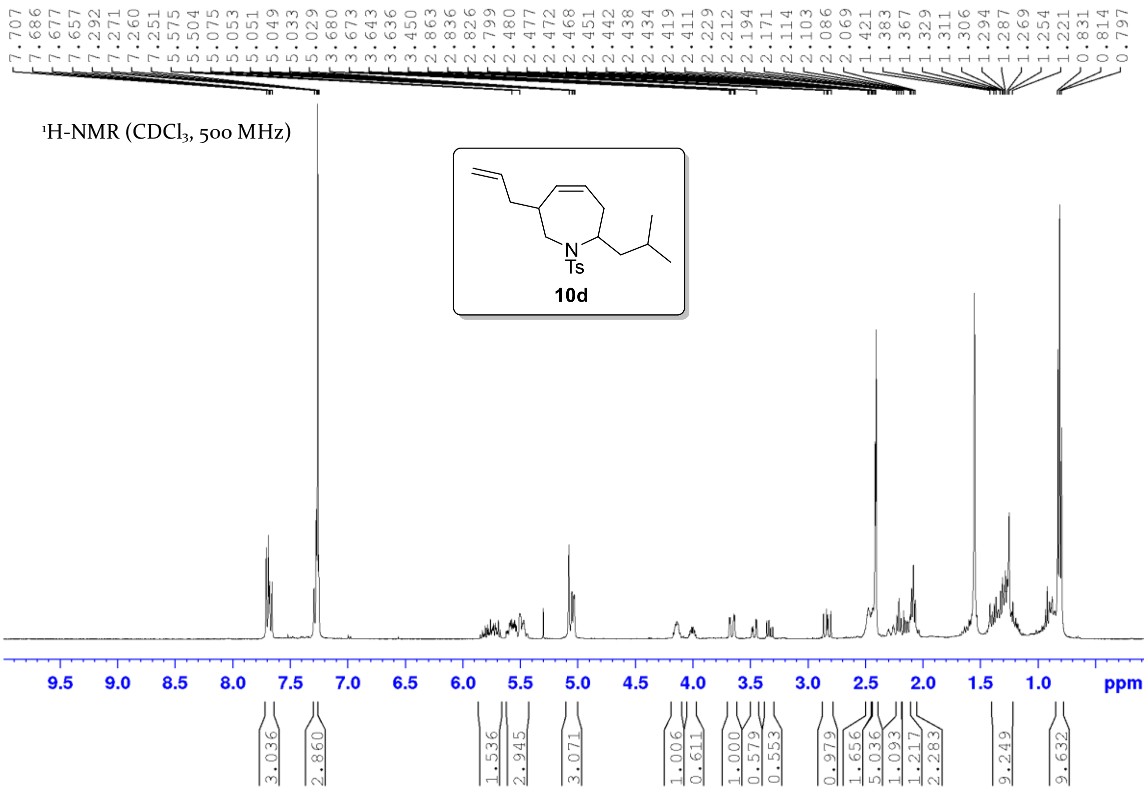
7.707 7.686 7.677 7.657 7.292 7.271 7.260 7.251 5.575 5.504 5.075 5.053 5.051 5.049 5.033 5.029 3.680 3.673 3.643 3.636 3.450 2.863 2.836 2.826 2.799 2.480 2.477 2.472 2.468 2.451 2.442 2.438 2.434 2.419 2.411 2.229 2.212 2.194 2.171 2.114 2.103 2.086 2.069 1.421 1.383 1.367 1.329 1.311 1.306 1.294 1.287 1.269 1.254 1.221 0.831 0.814 0.797
¹H-NMR (CDCl₃, 500 MHz)
N
Ts
10d
9.5 9.0 8.5 8.0 7.5 7.0 6.5 6.0 5.5 5.0 4.5 4.0 3.5 3.0 2.5 2.0 1.5 1.0 ppm
3.036 2.860 1.536 2.945 3.071 1.006 0.611 1.000 0.579 0.553 0.979 1.656 5.036 1.093 1.217 2.283 9.249 9.632

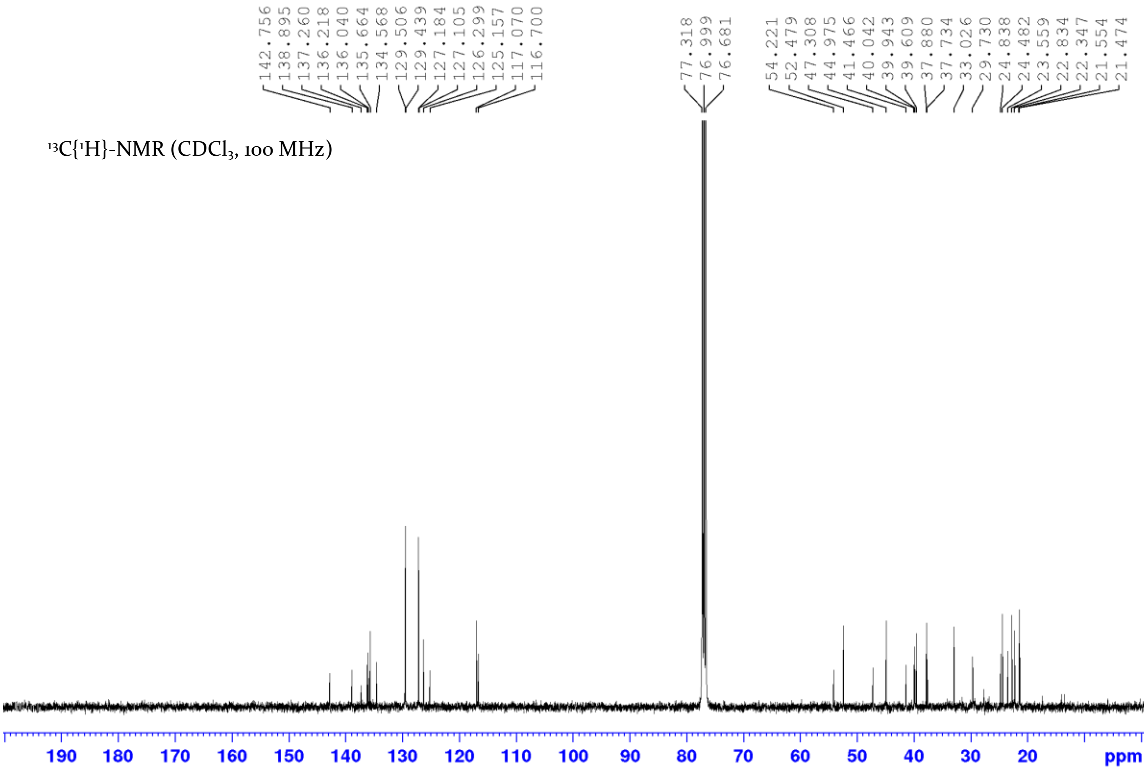
142.756 138.895 137.260 136.218 136.040 135.664 134.568 129.506 129.439 127.184 127.105 126.299 125.157 117.070 116.700
77.318 76.999 76.681
54.221 52.479 47.308 44.975 41.466 40.042 39.943 39.609 37.880 37.734 33.026 29.730 24.838 24.482 23.559 22.834 22.347 21.554 21.474
¹³C{¹H}-NMR (CDCl₃, 100 MHz)
190 180 170 160 150 140 130 120 110 100 90 80 70 60 50 40 30 20 ppm

4. Computational details

All the calculations reported in this paper were obtained with the Gaussian 09 suite of programs.[5] All species were optimized using the B3LYP functional[6] in conjunction with the D3 dispersion correction suggested by Grimme et al.[7] using the standard double-ζ quality def2-SVP[8] basis sets for all atoms. Solvents effects were taken into account during the geometry optimizations using the polarizable continuum model (PCM).[9] All stationary points were characterized by frequency calculations.[10] Reactants and products have positive definite Hessian matrices, whereas transition structures show only one negative eigenvalue in their diagonalized force constant matrices, and their associated eigenvectors were confirmed to correspond to the motion along the reaction coordinate under consideration using the intrinsic reaction coordinate (IRC) method.[11] Compounds involving iron(III) were computed using a high-spin (S = 5/2) configuration. Single-point energy refinements were carried out at the same DFT level the much larger triple-ζ quality def2-TZVPP basis sets. This level is denoted PCM(CH2Cl2)-B3LYP-D3/def2-TZVPP//PCM(CH2Cl2)-B3LYP-D3/def2-SVP.

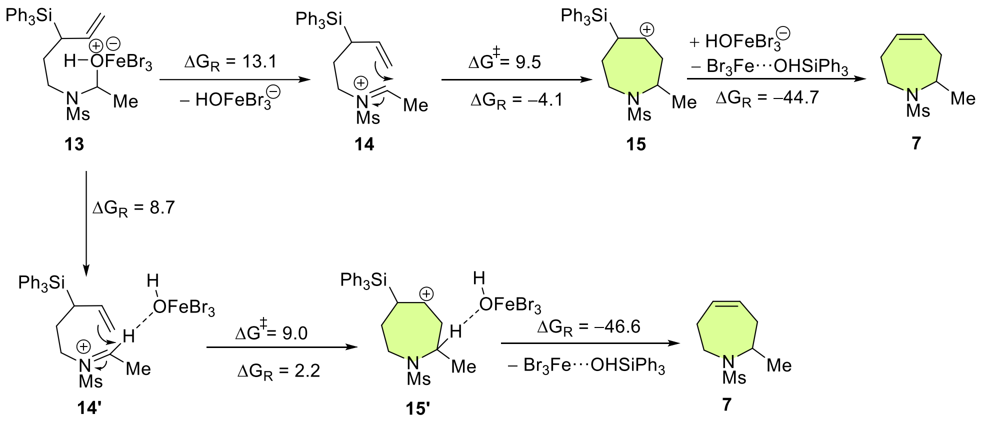

4.1. Cartesian coordinates (in Å) and electronic energies (in a.u.) of all the stationary points discussed in the text. All calculations have been performed at the PCM(CH2Cl2)-B3LYP-D3/def2-TZVPP//PCM(CH2Cl2)-B3LYP-D3/def2-SVP.

13: E= -10964.6881364

C	-0.933151000	-2.328487000	-1.661519000
C	-1.249596000	-1.684856000	-0.528912000
C	-1.808061000	-0.296116000	-0.395181000
C	1.384590000	0.683429000	0.135553000
C	-1.583098000	0.637057000	-1.597413000
C	-0.115376000	1.003567000	-1.840939000
H	-1.045065000	-1.871848000	-2.649098000
H	-1.114947000	-2.222516000	0.417015000
H	-1.344042000	0.166917000	0.494017000
H	-1.987932000	0.187656000	-2.518826000
H	1.091383000	-0.347949000	-0.104579000
H	-0.551199000	-3.352426000	-1.635938000
H	-2.136858000	1.575861000	-1.450163000
H	0.467672000	0.122117000	-2.144421000
H	-0.041771000	1.736016000	-2.655691000
N	0.543953000	1.546175000	-0.628877000
S	0.328432000	3.193337000	-0.260232000
C	1.941610000	3.930388000	-0.527343000
H	2.181007000	3.850346000	-1.595150000
H	1.852070000	4.981818000	-0.222485000
H	2.680625000	3.402983000	0.088746000

O	0.003588000	3.330446000	1.164766000
O	-0.602867000	3.707250000	-1.267802000
C	1.438293000	0.862703000	1.640309000
H	2.027453000	0.049403000	2.084372000
H	1.884562000	1.821387000	1.928211000
H	0.419888000	0.821391000	2.045587000
Si	-3.669856000	-0.346909000	0.076025000
C	-3.918124000	-1.496776000	1.555794000
C	-5.098634000	-2.248703000	1.712232000
C	-2.955545000	-1.563156000	2.583419000
C	-5.309101000	-3.039979000	2.847006000
H	-5.868105000	-2.220443000	0.935660000
C	-3.158909000	-2.353897000	3.718457000
H	-2.028022000	-0.987539000	2.507814000
C	-4.338330000	-3.095373000	3.852353000
H	-6.233271000	-3.615744000	2.945842000
H	-2.396276000	-2.390869000	4.500877000
H	-4.499730000	-3.714507000	4.738731000
C	-4.198738000	1.404624000	0.558421000
C	-5.559803000	1.770986000	0.573179000
C	-3.256044000	2.371655000	0.957371000
C	-5.960381000	3.054013000	0.960288000
H	-6.323240000	1.046614000	0.273678000
C	-3.648757000	3.658499000	1.337654000
H	-2.189584000	2.144721000	0.973859000
C	-5.003872000	4.003553000	1.339564000
H	-7.021986000	3.315655000	0.962126000
H	-2.885251000	4.386674000	1.622919000
H	-5.315707000	5.009080000	1.634477000
C	-4.644879000	-0.957277000	-1.417237000
C	-4.710248000	-2.335229000	-1.709905000
C	-5.234543000	-0.060874000	-2.330560000
C	-5.349380000	-2.800466000	-2.863418000
H	-4.256469000	-3.058344000	-1.027031000
C	-5.874607000	-0.521546000	-3.486225000
H	-5.196390000	1.014887000	-2.138953000
C	-5.934888000	-1.893341000	-3.753700000
H	-5.390531000	-3.873545000	-3.068577000
H	-6.328153000	0.192312000	-4.179055000
H	-6.437098000	-2.255399000	-4.654765000
O	2.792574000	0.821565000	-0.368849000
H	2.760051000	1.042147000	-1.316773000
Fe	4.292960000	-0.541196000	-0.017732000
Br	5.641016000	-0.189623000	-1.900541000
Br	3.124373000	-2.586280000	0.001995000
Br	5.201937000	0.117443000	2.041109000

14: E= -1902.1060825

C	0.809825000	-2.945748000	0.272647000
C	0.832875000	-1.653088000	0.638111000
C	0.538036000	-0.456565000	-0.220742000
C	3.537104000	-1.958422000	-0.730405000
C	0.864817000	-0.618258000	-1.720998000
C	2.337239000	-0.331624000	-2.076442000
H	1.004380000	-3.734607000	1.003670000
H	1.053797000	-1.423688000	1.685766000
H	1.094564000	0.402461000	0.186053000
H	0.586359000	-1.627343000	-2.056119000
H	3.116468000	-2.667679000	-1.454282000
H	0.562562000	-3.269396000	-0.742109000

H	0.265412000	0.070256000	-2.335779000
H	2.661076000	-0.870376000	-2.977952000
H	2.494098000	0.737261000	-2.255636000
N	3.269427000	-0.715328000	-0.978560000
S	3.779328000	0.662568000	0.173164000
C	5.521043000	0.788410000	-0.185969000
H	5.623196000	1.023320000	-1.252634000
H	5.877334000	1.619591000	0.439755000
H	6.019536000	-0.149199000	0.087047000
O	3.529748000	0.163105000	1.517825000
O	3.077755000	1.827263000	-0.347130000
C	4.400729000	-2.532907000	0.320448000
H	5.312694000	-2.907014000	-0.180759000
H	4.661205000	-1.849265000	1.131878000
H	3.892929000	-3.418989000	0.729538000
Si	-1.287367000	0.101041000	0.047078000
C	-1.580684000	0.290337000	1.901017000
C	-2.848830000	0.054661000	2.466785000
C	-0.552004000	0.742778000	2.752450000
C	-3.081032000	0.256023000	3.831706000
H	-3.670882000	-0.295857000	1.836295000
C	-0.778944000	0.944442000	4.117277000
H	0.447887000	0.945057000	2.356124000
C	-2.045391000	0.700046000	4.660260000
H	-4.073083000	0.064732000	4.249214000
H	0.034621000	1.293101000	4.758787000
H	-2.224237000	0.856095000	5.727282000
C	-1.502960000	1.768394000	-0.814567000
C	-2.786769000	2.215282000	-1.187773000
C	-0.411813000	2.630966000	-1.044837000
C	-2.974604000	3.469756000	-1.776379000
H	-3.657459000	1.574784000	-1.019562000
C	-0.595749000	3.886515000	-1.634209000
H	0.602305000	2.337139000	-0.760826000
C	-1.877367000	4.307777000	-2.003455000
H	-3.979636000	3.794167000	-2.058720000
H	0.265635000	4.537951000	-1.803854000
H	-2.021589000	5.288042000	-2.465177000
C	-2.408149000	-1.211383000	-0.703535000
C	-2.719874000	-2.380046000	0.021251000
C	-2.861676000	-1.119162000	-2.034703000
C	-3.464518000	-3.412410000	-0.557436000
H	-2.376854000	-2.486906000	1.053375000
C	-3.605801000	-2.150195000	-2.617950000
H	-2.635655000	-0.229303000	-2.628446000
C	-3.909866000	-3.298538000	-1.879083000
H	-3.697867000	-4.308138000	0.024134000
H	-3.950608000	-2.056018000	-3.651025000
H	-4.494133000	-4.103714000	-2.332216000

TS(14-15): E= -1902.0943715

C	1.487796000	-2.579779000	0.125817000
C	1.035929000	-1.346273000	0.571090000
C	0.584867000	-0.274538000	-0.288674000
C	3.308640000	-1.912829000	-0.547476000
C	0.858507000	-0.435319000	-1.796355000
C	2.361515000	-0.330581000	-2.154941000
H	1.733693000	-3.344723000	0.865061000
H	1.132750000	-1.114025000	1.635764000
H	0.966705000	0.680203000	0.111221000
H	0.437786000	-1.391201000	-2.140709000

H	3.226243000	-2.605303000	-1.390436000
H	1.140623000	-2.952559000	-0.839931000
H	0.321145000	0.342720000	-2.355714000
H	2.636005000	-1.037371000	-2.951308000
H	2.603997000	0.674617000	-2.514880000
N	3.255970000	-0.613974000	-1.004264000
S	3.640775000	0.748921000	0.034363000
C	5.408520000	0.909088000	-0.148012000
H	5.627309000	1.054698000	-1.213236000
H	5.680623000	1.797791000	0.438264000
H	5.897228000	0.014553000	0.254007000
O	3.310449000	0.376815000	1.414378000
O	2.999941000	1.915708000	-0.576117000
C	4.289440000	-2.353957000	0.501844000
H	5.306203000	-2.212383000	0.096159000
H	4.197375000	-1.795231000	1.439419000
H	4.162925000	-3.425670000	0.696388000
Si	-1.323271000	0.084611000	0.063975000
C	-1.544314000	0.179545000	1.927568000
C	-2.701285000	-0.325412000	2.551608000
C	-0.582719000	0.827863000	2.730339000
C	-2.888867000	-0.194158000	3.931865000
H	-3.467800000	-0.829728000	1.957202000
C	-0.765732000	0.956266000	4.109718000
H	0.326984000	1.243576000	2.284901000
C	-1.920752000	0.444702000	4.712861000
H	-3.793343000	-0.593129000	4.398020000
H	-0.006824000	1.458287000	4.715010000
H	-2.065470000	0.545372000	5.791493000
C	-1.658192000	1.747837000	-0.749116000
C	-2.994938000	2.114498000	-1.008719000
C	-0.643955000	2.682740000	-1.035675000
C	-3.307486000	3.369056000	-1.540141000
H	-3.807863000	1.414995000	-0.792971000
C	-0.955267000	3.938097000	-1.568469000
H	0.408112000	2.449841000	-0.847970000
C	-2.286561000	4.282955000	-1.822867000
H	-4.350136000	3.633565000	-1.733576000
H	-0.153450000	4.648450000	-1.785073000
H	-2.528728000	5.263686000	-2.240093000
C	-2.286072000	-1.319995000	-0.718465000
C	-2.378380000	-2.566046000	-0.064370000
C	-2.840523000	-1.201588000	-2.008195000
C	-3.013700000	-3.652101000	-0.672674000
H	-1.952268000	-2.694158000	0.934881000
C	-3.476722000	-2.287164000	-2.619141000
H	-2.777744000	-0.252254000	-2.546117000
C	-3.565719000	-3.512828000	-1.951540000
H	-3.079609000	-4.608680000	-0.148271000
H	-3.903790000	-2.175105000	-3.618894000
H	-4.064363000	-4.360966000	-2.427602000

7: E= -917.5074536

C	1.421855000	1.435761000	0.718899000
C	2.053845000	0.168455000	1.242916000
C	2.372160000	-0.939543000	0.555972000
C	0.593681000	1.300880000	-0.583634000
C	2.174453000	-1.123317000	-0.930584000
C	0.694911000	-1.081771000	-1.371626000
H	0.763863000	1.830582000	1.507288000
H	2.263640000	0.174439000	2.318917000

H	2.808827000	-1.777838000	1.109416000
H	2.736784000	-0.345216000	-1.474831000
H	1.285142000	1.316436000	-1.438579000
H	2.199700000	2.206977000	0.558449000
H	2.598066000	-2.083834000	-1.262154000
H	0.640598000	-0.942368000	-2.465434000
H	0.210945000	-2.036515000	-1.135364000
N	-0.072486000	-0.016087000	-0.713143000
S	-1.292573000	-0.451613000	0.350670000
C	-2.801610000	-0.194210000	-0.588806000
H	-2.772280000	-0.844694000	-1.472132000
H	-3.636699000	-0.467486000	0.070587000
H	-2.859850000	0.862547000	-0.876924000
O	-1.314349000	0.496875000	1.475683000
O	-1.204659000	-1.894084000	0.627115000
C	-0.361893000	2.479193000	-0.776895000
H	-0.886666000	2.402575000	-1.741488000
H	-1.103420000	2.526357000	0.033062000
H	0.207569000	3.421427000	-0.769365000

$Br_3Fe\cdots OHSiPH_3$: E= -10047.2199483

Si	1.612141000	0.059794000	0.024604000
C	2.493046000	1.616756000	-0.517331000
C	1.745025000	2.790425000	-0.749906000
C	3.890092000	1.666579000	-0.685904000
C	2.377956000	3.978659000	-1.126322000
H	0.655794000	2.778351000	-0.644142000
C	4.523983000	2.855352000	-1.060270000
H	4.494522000	0.768600000	-0.531464000
C	3.768672000	4.012787000	-1.278834000
H	1.784526000	4.879252000	-1.303222000
H	5.609394000	2.877269000	-1.186470000
H	4.263916000	4.941696000	-1.573105000
C	2.464722000	-1.539773000	-0.421705000
C	1.891817000	-2.426826000	-1.353535000
C	3.682924000	-1.902548000	0.188843000
C	2.522876000	-3.632808000	-1.675154000
H	0.935902000	-2.183753000	-1.825277000
C	4.315315000	-3.106668000	-0.132969000
H	4.143966000	-1.247470000	0.934453000
C	3.736184000	-3.972485000	-1.067882000
H	2.063132000	-4.310828000	-2.398632000
H	5.258942000	-3.372656000	0.350002000
H	4.228706000	-4.915545000	-1.318291000
C	1.033165000	0.087628000	1.794991000
C	0.600543000	1.285922000	2.396624000
C	0.971311000	-1.102452000	2.546029000
C	0.100312000	1.290651000	3.700805000
H	0.642573000	2.225967000	1.841415000
C	0.479745000	-1.098474000	3.853749000
H	1.297648000	-2.045575000	2.100549000
C	0.038803000	0.097716000	4.429839000
H	-0.243376000	2.226161000	4.148952000
H	0.431557000	-2.031125000	4.421186000
H	-0.353325000	0.101031000	5.450205000
O	0.130633000	0.097574000	-0.916488000
H	0.250695000	0.266706000	-1.867346000
Fe	-1.848552000	-0.034525000	-0.439592000
Br	-2.202282000	-1.829874000	1.015594000
Br	-2.649303000	-0.444989000	-2.628046000

Br	-2.429450000	2.072555000	0.434498000

14': E= -10964.6744878

C	0.148891000	0.380068000	1.823938000
C	-1.033582000	0.830408000	1.378633000
C	-1.623970000	0.637960000	0.008265000
C	1.097147000	2.787480000	0.137380000
C	-0.605444000	0.465307000	-1.140046000
C	-0.078759000	1.784057000	-1.731127000
H	0.463267000	0.561224000	2.854608000
H	-1.673059000	1.377526000	2.078927000
H	-2.277765000	1.498883000	-0.205272000
H	0.242567000	-0.145702000	-0.801225000
H	1.857587000	2.032012000	-0.139813000
H	0.840653000	-0.194009000	1.204993000
H	-1.045507000	-0.089590000	-1.983478000
H	0.910419000	1.634640000	-2.183604000
H	-0.766565000	2.193899000	-2.478110000
N	0.092325000	2.833247000	-0.682561000
S	-1.312625000	4.009388000	-0.471750000
C	-0.545348000	5.554865000	-0.932336000
H	-0.174049000	5.453921000	-1.959882000
H	-1.353443000	6.298039000	-0.874662000
H	0.253998000	5.792170000	-0.220628000
O	-1.666833000	3.989228000	0.942065000
O	-2.274167000	3.616665000	-1.495197000
C	1.402694000	3.667893000	1.280324000
H	2.253103000	4.306881000	0.977525000
H	0.570284000	4.276352000	1.643417000
H	1.793850000	3.025093000	2.083673000
Si	-2.879485000	-0.815605000	0.048370000
C	-4.057098000	-0.552112000	1.502445000
C	-4.598097000	-1.639759000	2.214429000
C	-4.471667000	0.745350000	1.866609000
C	-5.512867000	-1.442012000	3.254830000
H	-4.297833000	-2.659730000	1.958826000
C	-5.384228000	0.948855000	2.906121000
H	-4.079715000	1.619756000	1.337663000
C	-5.906637000	-0.146240000	3.603958000
H	-5.917025000	-2.301993000	3.795577000
H	-5.687943000	1.964506000	3.173452000
H	-6.618498000	0.011076000	4.418579000
C	-3.860755000	-0.772762000	-1.568776000
C	-4.589816000	-1.904900000	-1.986062000
C	-3.944513000	0.391623000	-2.358627000
C	-5.365646000	-1.880588000	-3.148980000
H	-4.551178000	-2.825532000	-1.396149000
C	-4.719534000	0.420434000	-3.523507000
H	-3.408096000	1.299894000	-2.070496000
C	-5.430501000	-0.716047000	-3.922302000
H	-5.920623000	-2.772128000	-3.453043000
H	-4.768466000	1.334901000	-4.120749000
H	-6.034992000	-0.694618000	-4.832938000
C	-1.933041000	-2.432290000	0.226317000
C	-1.437298000	-2.828703000	1.485792000
C	-1.602850000	-3.224080000	-0.891106000
C	-0.652412000	-3.976597000	1.624500000
H	-1.663801000	-2.231397000	2.372555000
C	-0.817364000	-4.373202000	-0.756321000
H	-1.960815000	-2.941155000	-1.884642000
C	-0.341957000	-4.752205000	0.502642000

H	-0.274887000	-4.262626000	2.609704000
H	-0.570543000	-4.970738000	-1.637816000
H	0.277813000	-5.646326000	0.608764000
O	2.943914000	0.996431000	-1.380235000
H	3.579522000	1.593360000	-1.805320000
Fe	3.663866000	-0.275654000	-0.239944000
Br	3.781452000	0.748501000	1.945674000
Br	2.287343000	-2.226588000	-0.298633000
Br	5.890471000	-0.710240000	-0.985846000

15’: E= -10964.6721859

C	-0.153262000	1.387775000	-1.273716000
C	1.267516000	1.196666000	-0.936595000
C	1.699818000	0.707052000	0.304044000
C	-0.842316000	2.467729000	-0.323902000
C	0.667974000	0.492215000	1.405161000
C	0.070891000	1.830449000	1.908467000
H	-0.270210000	1.676461000	-2.324210000
H	2.023170000	1.445772000	-1.685567000
H	2.633193000	1.217706000	0.610407000
H	-0.135750000	-0.149972000	1.016612000
H	-1.708191000	1.973833000	0.133022000
H	-0.682839000	0.427330000	-1.125020000
H	1.107423000	-0.047781000	2.254510000
H	-0.950349000	1.651165000	2.272156000
H	0.673834000	2.239975000	2.726430000
N	-0.020800000	2.849008000	0.847829000
S	1.330372000	3.837677000	0.622090000
C	0.758500000	5.492243000	0.980673000
H	0.406034000	5.505661000	2.019481000
H	1.625523000	6.153261000	0.847403000
H	-0.044048000	5.749968000	0.280523000
O	1.718476000	3.784095000	-0.806294000
O	2.346444000	3.487511000	1.630403000
C	-1.360968000	3.656495000	-1.125615000
H	-1.881667000	4.357762000	-0.455377000
H	-0.557004000	4.184636000	-1.655970000
H	-2.090940000	3.292684000	-1.861697000
Si	2.860790000	-0.931226000	-0.078136000
C	4.098100000	-0.372349000	-1.374564000
C	4.504804000	-1.259299000	-2.391142000
C	4.703958000	0.900695000	-1.319955000
C	5.478224000	-0.886278000	-3.323816000
H	4.061033000	-2.255637000	-2.459455000
C	5.670941000	1.277012000	-2.255284000
H	4.428125000	1.624165000	-0.546476000
C	6.060263000	0.383087000	-3.259448000
H	5.779840000	-1.589747000	-4.103850000
H	6.122745000	2.270324000	-2.198852000
H	6.817498000	0.677166000	-3.990516000
C	3.696594000	-1.241067000	1.575648000
C	4.279807000	-2.508327000	1.787227000
C	3.873813000	-0.254090000	2.564508000
C	5.004096000	-2.780720000	2.951330000
H	4.176711000	-3.295971000	1.035909000
C	4.596625000	-0.525991000	3.729741000
H	3.453159000	0.748488000	2.443868000
C	5.160494000	-1.790686000	3.926611000
H	5.446637000	-3.769399000	3.095762000
H	4.718466000	0.253783000	4.485409000

H	5.723057000	-2.003790000	4.838980000
C	1.761504000	-2.320415000	-0.657269000
C	1.282569000	-2.327685000	-1.983769000
C	1.365202000	-3.361216000	0.204573000
C	0.454179000	-3.354230000	-2.439964000
H	1.559206000	-1.527022000	-2.676265000
C	0.541422000	-4.392999000	-0.253881000
H	1.695151000	-3.369651000	1.245846000
C	0.088650000	-4.392538000	-1.575915000
H	0.089351000	-3.342984000	-3.469760000
H	0.242058000	-5.192308000	0.428105000
H	-0.562769000	-5.194686000	-1.931269000
O	-3.070291000	1.036952000	1.553798000
H	-3.588285000	1.843107000	1.702001000
Fe	-3.691349000	-0.185075000	0.328062000
Br	-3.881522000	0.896880000	-1.824077000
Br	-2.069756000	-1.947539000	0.114171000
Br	-5.816148000	-1.053931000	1.013956000

TS(14′-15′): E= -10964.6618651

C	0.261968000	0.785108000	1.344324000
C	-1.095987000	0.931856000	1.103935000
C	-1.735778000	0.636134000	-0.160579000
C	0.854179000	2.414589000	0.339527000
C	-0.799466000	0.372195000	-1.357540000
C	-0.002458000	1.623212000	-1.808181000
H	0.640169000	0.946530000	2.354695000
H	-1.713330000	1.408409000	1.870646000
H	-2.482266000	1.421165000	-0.368967000
H	-0.110555000	-0.446206000	-1.102632000
H	1.756652000	1.888032000	0.003905000
H	0.824578000	0.057029000	0.755940000
H	-1.389996000	0.012580000	-2.211818000
H	1.043148000	1.368091000	-2.032310000
H	-0.449093000	2.070955000	-2.701967000
N	0.058806000	2.680543000	-0.765184000
S	-1.292842000	3.772686000	-0.682296000
C	-0.553465000	5.363122000	-1.018655000
H	-0.077199000	5.310971000	-2.005382000
H	-1.385931000	6.080211000	-1.017032000
H	0.167404000	5.603842000	-0.229409000
O	-1.829570000	3.763407000	0.686155000
O	-2.175963000	3.432488000	-1.803100000
C	1.126562000	3.455958000	1.388479000
H	1.658568000	4.292740000	0.903572000
H	0.221981000	3.840310000	1.872392000
H	1.807370000	3.032899000	2.137213000
Si	-3.000014000	-0.846715000	0.089098000
C	-3.996120000	-0.501491000	1.648800000
C	-4.383573000	-1.546119000	2.509943000
C	-4.423255000	0.806965000	1.957028000
C	-5.163113000	-1.293107000	3.643957000
H	-4.070393000	-2.572129000	2.299123000
C	-5.198737000	1.063389000	3.091066000
H	-4.149647000	1.648967000	1.312889000
C	-5.569642000	0.012154000	3.937666000
H	-5.450881000	-2.117801000	4.301109000
H	-5.514259000	2.085623000	3.314764000
H	-6.175059000	0.211407000	4.825636000
C	-4.104759000	-0.814199000	-1.436017000
C	-4.883553000	-1.953328000	-1.726312000

C	-4.253956000	0.320404000	-2.257958000
C	-5.779175000	-1.960523000	-2.799549000
H	-4.793719000	-2.850814000	-1.107090000
C	-5.149754000	0.314669000	-3.332574000
H	-3.672557000	1.229107000	-2.076124000
C	-5.913416000	-0.824783000	-3.605543000
H	-6.373354000	-2.854253000	-3.006729000
H	-5.249710000	1.204792000	-3.958914000
H	-6.612319000	-0.828352000	-4.445930000
C	-1.978695000	-2.411459000	0.216971000
C	-1.320593000	-2.729484000	1.422806000
C	-1.738017000	-3.223491000	-0.908270000
C	-0.457796000	-3.825082000	1.502785000
H	-1.476589000	-2.112492000	2.312440000
C	-0.876184000	-4.321819000	-0.829684000
H	-2.223020000	-2.994631000	-1.860756000
C	-0.235022000	-4.623254000	0.375526000
H	0.049151000	-4.052088000	2.443839000
H	-0.698497000	-4.938652000	-1.714197000
H	0.447192000	-5.474909000	0.434888000
O	3.197641000	1.192741000	-1.360985000
H	3.736998000	1.992377000	-1.461770000
Fe	3.821339000	-0.158881000	-0.272769000
Br	3.770283000	0.661008000	2.007346000
Br	2.319340000	-2.017748000	-0.503912000
Br	6.062272000	-0.733099000	-0.856368000

5. References

[1] Panek, J. S.; Beresis, R.; Xu, F.; Yang, M. Diastereoselective Electrophilic Addition Reactions to Chiral .Beta.-Dimethylphenylsilyl Ester Enolates. Synthesis of 2,3-Anti-.Alpha.-Substituted-.Beta.-Silyl-(E)-Hex-4-Enoates. *J. Org. Chem.* **1991**, *56*, 7341–7344.

[2] Corriu, R.; Masse, J. Organométalliques Allyliques Siliciés: Obtention et Comportement. *J. Organomet. Chem.* **1973**, *57*, C5–C8.

[3] Corriu, R. J. P.; Masse, J.; Samate, D. Syntheses a Partir de Carbanions Allyliques Silicies: I. Carbanions Derives de Monoallylsilanes. *J. Organomet. Chem.* **1975**, *93*, 71–80.

[4] Cruz, D. A.; Sinka, V.; Martín, V. S.; Padrón, J. I. Iron-Catalyzed Prins–Peterson Reaction for the Direct Synthesis of Δ4-2,7-Disubstituted Oxepenes. *J. Org. Chem.* **2018**, *83*, 12632–12647.

[5] Gaussian 09, Revision D.01, Frisch, M. J.; Trucks, G. W.; Schlegel, H. B.; Scuseria, G. E.; Robb, M. A.; Cheeseman, J. R.; Scalmani, G.; Barone, V.; Mennucci, B.; Petersson, G. A.; Nakatsuji, H.; Caricato, M.; Li, X.; Hratchian, H. P.; Izmaylov, A. F.; Bloino, J.; Zheng, G.; Sonnenberg, J. L.; Hada, M.; Ehara, M.; Toyota, K.; Fukuda, R.; Hasegawa, J.; Ishida, M.; Nakajima, T.; Honda, Y.; Kitao, O.; Nakai, H.; Vreven, T.; Montgomery, J. A., Jr.; Peralta, J. E.; Ogliaro, F.; Bearpark, M.; Heyd, J. J.; Brothers, E.; Kudin, K. N.; Staroverov, V. N.; Kobayashi, R.; Normand, J.; Raghavachari, K.; Rendell, A.; Burant, J. C.; Iyengar, S. S.; Tomasi, J.; Cossi, M.; Rega, N.; Millam, J. M.; Klene, M.; Knox, J. E.; Cross, J. B.; Bakken, V.; Adamo, C.; Jaramillo, J.; Gomperts, R.; Stratmann, R. E.; Yazyev, O.; Austin, A. J.; Cammi, R.; Pomelli, C.; Ochterski, J. W.; Martin, R. L.; Morokuma, K.; Zakrzewski, V. G.; Voth, G. A.; Salvador, P.; Dannenberg, J. J.; Dapprich, S.; Daniels, A. D.; Farkas, Ö.; Foresman, J. B.; Ortiz, J. V.; Cioslowski, J.; Fox, D. J. Gaussian, Inc., Wallingford CT, 2009.

[6] (a) Becke, A. D. Density-functional thermochemistry. III. The role of exact exchange. *J. Chem. Phys.* **1993**, *98*, 5648. (b) Lee, C.; Yang, W.; Parr, R. G. Development of the Colle-Salvetti correlation-energy formula into a functional of the electron density. *Phys. Rev. B* **1988**, *37*,

785-789. (c) Vosko, S. H.; Wilk, L.; Nusair, M. Accurate spin-dependent electron liquid correlation energies for local spin density calculations: a critical analysis. *Can. J. Phys.* **1980**, *58*, 1200-1211.

[7] Grimme, S.; Antony, J.; Ehrlich, S.; Krieg, H. A consistent and accurate ab initio parametrization of density functional dispersion correction (DFT-D) for the 94 elements H-Pu. *J. Chem. Phys.* **2010**, *132*, 154104.

[8] Weigend, F.; Ahlrichs, R. Balanced basis sets of split valence, triple zeta valence and quadruple zeta valence quality for H to Rn: Design and assessment of accuracy. *Phys. Chem. Chem. Phys.* **2005**, *7*, 3297-3305.

[9] (a) Miertuš, S.; Scrocco, E.; Tomasi, J. Electrostatic interaction of a solute with a continuum. A direct utilizaion of AB initio molecular potentials for the prevision of solvent effects. *Chem. Phys.* **1981**, *55*, 117-129. (b) Pascual-Ahuir, J. L.; Silla, E.; Tuñón, I. GEPOL: An improved description of molecular surfaces. III. A new algorithm for the computation of a solvent-excluding Surface. *J. Comp. Chem.* **1994**, *15*, 1127-1138. (c) Barone, V.; Cossi, M. Quantum Calculation of Molecular Energies and Energy Gradients in Solution by a Conductor Solvent Model. *J. Phys. Chem. A*, **1998**, *102*, 1995-2001.

[10] McIver, J. W.; Komornicki, A. K. Structure of transition states in organic reactions. General theory and an application to the cyclobutene-butadiene isomerization using a semiempirical molecular orbital method. *J. Am. Chem. Soc.* **1972**, *94*, 2625-2633.

[11] González, C.; Schlegel, H. B. Reaction path following in mass-weighted internal coordinates. *J. Phys. Chem.* **1990**, *94*, 5523-5527.
